# Optimizing OPM-MEG Sensor Layouts Using the Sequential Selection Algorithm with Simulated Sources and Individual Anatomy

**DOI:** 10.3390/s26041292

**Published:** 2026-02-17

**Authors:** Urban Marhl, Rok Hren, Tilmann Sander, Vojko Jazbinšek

**Affiliations:** 1 Institute of Mathematics, Physics and Mechanics, 1000 Ljubljana, Slovenia; urban.marhl@imfm.si (U.M.); vojko.jazbinsek@imfm.si (V.J.); 2 Faculty of Natural Sciences and Mathematics, University of Maribor, 2000 Maribor, Slovenia; 3 Syreon Research Institute, 1142 Budapest, Hungary; 4 Physikalisch-Technische Bundesanstalt, 10587 Berlin, Germany; tilmann.sander-thoemmes@ptb.de

**Keywords:** magnetoencephalography, optically pumped magnetometers, sensor optimization, sequential selection algorithm, auditory-evoked fields, magnetic field maps

## Abstract

Magnetoencephalography (MEG) based on optically pumped magnetometers (OPMs) offers the flexibility to position sensors closer to the scalp, which improves the signal-to-noise ratio compared to conventional superconducting quantum interference device (SQUID) systems. However, the spatial resolution of OPM-MEG critically depends on sensor placement, especially when the number of sensors is limited. In this study, we present a methodology for optimizing OPM-MEG sensor layouts using each subject’s anatomical information derived from individual magnetic resonance imaging (MRI). We generated realistic forward models from reconstructed head surfaces and simulated magnetic fields produced by equivalent current dipoles (ECDs). We compared multiple simulation strategies, including ECDs randomly distributed across the cortical surface and ECDs constrained to regions of interest. For each simulated magnetic field map (MFM) database, we applied the sequential selection algorithm (SSA) to identify sensor positions that maximized information capture. Unlike previous approaches relying on large measurement databases, this simulation-driven strategy eliminates the need for extensive pre-existing recordings. We benchmarked the performance of the personalized layouts using OPM-MEG datasets of auditory evoked fields (AEFs) derived from real whole-head SQUID-MEG measurements. Our results show that simulation-based SSA optimization improves the coverage of cortical regions of interest, reduces the number of sensors required for accurate source reconstruction, and yields sensor configurations that perform comparably to layouts optimized using measured data. To evaluate the quality of estimated MFMs, we applied metrics such as the correlation coefficient (CC), root-mean-square error, and relative error. Our results show that the first 15 to 20 optimally selected sensors (CC > 0.95) capture most of the information contained in full-head MFMs. Additionally, we performed source localization for the highest auditory response (M100) by fitting equivalent current dipoles and found that localization errors were < 5 mm. The results further indicate that SSA performance is insensitive to individualized head geometry, supporting the feasibility of using representative anatomical models and highlighting the potential of this approach for clinical OPM-MEG applications.

## 1. Introduction

Magnetoencephalography (MEG) is a key noninvasive neuroimaging method that records femtotesla-level magnetic fields generated by neuronal currents with high temporal precision [1]. It is used for advancing both basic neuroscience and clinical diagnostics. Until recently, conventional commercial MEG systems used superconducting quantum interference devices (SQUIDs), which have excellent sensitivity but require cryogenic cooling, and the sensor arrays are fixed inside the rigid Dewar [2].

Optically pumped magnetometers (OPMs) have recently emerged as a viable alternative for MEG [3]. These sensors operate at room temperature and can be placed directly on the scalp, thereby increasing the signal-to-noise ratio (SNR) [4,5], improving spatial resolution [6], and enabling subject-specific sensor configurations [7,8,9].

Early OPM-MEG studies have demonstrated strong potential in both research and clinical contexts, including studies of evoked responses, pediatric neuroimaging, and epilepsy source localization [10]. However, practical limitations remain: commercial OPMs are still costly, and complete whole-head OPM-MEG systems require dozens of sensors, making optimized placement strategies essential, particularly when only a limited number of sensors are available.

Among the earlier studies, Zhang et al. [11] demonstrated that wearable OPM-based MEG could reliably detect occipital alpha rhythm modulation (photic blocking), with performance comparable to conventional SQUID-MEG, while allowing sensors to be positioned closer to the scalp. Wearable OPM-MEG could further measure cortical tracking of speech (CTS) at both lower (phrasal) and higher (word/syllabic) frequencies, with results comparable to conventional SQUID-MEG when appropriate preprocessing is applied [12], as well as whole-brain functional connectivity with fidelity comparable to conventional cryogenic MEG, including both resting-state and task-based networks [13]. High-fidelity, real-time neural decoding comparable to conventional MEG can also be achieved [14].

The capability of OPM-MEG opened the door to routine clinical use of MEG in patient populations previously difficult to assess, such as children, movement-disordered patients, or those unable to tolerate rigid scanners, while preserving clinically meaningful information about network dysfunction relevant to conditions like epilepsy [15,16,17,18,19,20,21,22,23,24,25], neurodevelopmental disorders [26,27,28,29,30], and neurodegenerative diseases [30,31,32].

A promising approach is to compute optimal sensor locations using a sequential selection algorithm (SSA) [33]. Earlier work has shown that the algorithm can be applied to MCG and SQUID-MEG [34,35,36,37,38]. Recently, we showed that the SSA can effectively optimize OPM-MEG sensor layouts, enabling a limited number of strategically placed sensors to capture critical neural activity [37]. Using the SSA for low sensor count measurements enhances spatial resolution and reconstruction accuracy [34,38]. To date, the SSA has been trained using only real, empirical measurements.

In this study, we present a framework for optimizing OPM-MEG sensor layouts based on forward simulations derived from each subject’s magnetic resonance imaging (MRI). We generated forward models and computed magnetic field maps (MFMs) for large sets of equivalent current dipoles (ECDs), either distributed across the entire cortical surface or restricted to functionally relevant regions of interest (ROIs). The SSA was applied to these simulated MFMs to identify sensor positions that yield maximal information capture for each individual, without the need for any prior measurements. This has the potential for several applications: for instance, it could support future commercial low-sensor-count OPM-MEG systems by providing optimized, subject-specific layouts without requiring preliminary recordings. We validated the resulting layouts using OPM-MEG data, which were derived from SQUID measurements of auditory evoked fields [39].

The main goal of this study was to test the hypotheses central to this approach by applying the SSA method to simulated data. First, we examined whether using a subject’s individualized head geometry is necessary/advantageous for computing an optimal sensor layout. We tested this by applying the optimized layouts to the head geometry of a different subject and then benchmarking their performance. Second, we evaluated whether restricting simulations to a specific region or by using two simultaneous ECDs would yield better sensor configurations for a particular evoked response, such as auditory evoked fields (AEFs). Lastly, we examined whether using a more sophisticated forward model, the “single-layer boundary element method (BEM)”, offers any advantage over a simple spherical volume conductor (SPH), allowing us to assess the impact of forward model complexity on the quality of the optimized sensor layouts.

Overall, our findings demonstrate that the SSA applied to simulated MFMs yields effective, generalizable OPM-MEG sensor layouts. These optimized configurations improve cortical coverage, reduce the number of sensors required for accurate source reconstruction, and outperform generic arrangements, offering a practical path toward high-performance OPM-MEG systems even when sensor count is limited.

## 2. Materials and Methods

In this section, we describe the methodology for creating and evaluating optimized OPM-MEG sensor layouts using the SSA based on simulated data. The resulting sensor configurations were subsequently validated using auditory evoked field measurements, which were transformed into hypothetical OPM-MEG sensor layouts for performance benchmarking. We used the same dataset as presented in our previous publication, comprising 16 measurements from 9 subjects [37]. For all subjects, structural MRI scans of the head were available and used to reconstruct individual head geometry. All recordings were conducted in healthy adult participants under protocols approved by the local ethics committee, and written informed consent was obtained from all subjects.

### 2.1. Construction of a Hypothetical Unitary Sensor Holder

For each participant, we conducted structural MRI, which was used for surface reconstruction, co-registration, and forward modeling. The T1-weighted images were reconstructed using the FreeSurfer software package (Version 7.3.2) with the standard “recon-all” pipeline, which performs automated cortical and subcortical segmentation and yields individually segmented head tissues, such as gray matter and white matter [40,41].

To define a common hypothetical whole-head OPM-MEG sensor geometry, the reconstructed outer head surfaces of all nine subjects were aligned to determine the biggest sensor holder geometry needed. Based on this combined outer surface, we defined five circles at different vertical levels, ranging from approximately eye height to the top of the head. Sensors were then placed equidistantly along each circle. Each sensor was assigned two orthogonal sensing directions, corresponding to radial and tangential components. The radial orientation was defined to point toward the center of the lowest ellipse. Next, the sensor positions were translated inward along the radial direction to minimize the distance to the combined outer head surface while avoiding intersection with it. Using this approach, the sensors in the lowest ring were positioned at distances of 8.9–12.1 cm from the center, with an average of 10.0 cm.

**Figure 1:**
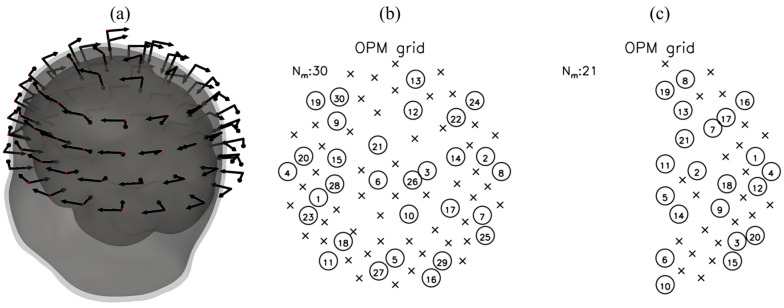
(**a**) Whole-head OPM-MEG system with 80 measuring sites, where each arrow represents a sensing direction of two axial OPM sensors. The gray surfaces represent reconstructed BEM surfaces for one subject. (**b**) Whole-head OPM grid with selected (circle) and unselected (cross) measuring sites for the first 30 optimally selected measuring sited using all–bases SPH simulated data from the whole-head OPM-MEG system. (**c**) Right-hemisphere OPM grid for 21 optimally selected measuring sites using all–bases SPH simulated data from 43 measuring sites on the right hemisphere only. Encircled numbers show the order of selected sites.

Using this procedure, a total of 80 sensor locations were defined across the five rings, forming a whole-head OPM-MEG array. Each location comprised two orthogonal sensing directions (radial and tangential), resulting in 160 measurement channels in the forward simulations. A visualization of the resulting sensor array relative to the reconstructed head surfaces is shown in Figure 1a. A more detailed description of the sensor holder design and construction procedure is provided in our previous work [37].

### 2.2. Measurements of Auditory Evoked Fields (AEFs)

The AEF data used in this study were previously acquired and described in detail in our earlier works [37,39]. Briefly, AEFs were recorded inside a magnetically shielded room at the Physikalisch-Technische Bundesanstalt (PTB) in Berlin (AK3b, VAC, Hanau, Germany). We used a whole-head SQUID-MEG system with 125 first-order gradiometers, produced by KIT (Kanazawa Institute of Technology, later marketed by Yokogawa, Musashino, Japan) [42]. Participants were presented with 500 tones at 1 kHz and 500 ms in duration, with an inter-stimulus interval of 1.2 s. A total of nine healthy participants were included. For a subset of participants, repeated measurements were acquired in separate sessions. The measurement details are provided in Appendix A, Table A1.

Our goal was to benchmark the layout selection with the SSA for a unitary OPM-MEG layout; therefore, we transformed the measurements to the hypothetical unitary sensor holder layout presented in Section 2.1. Previous work has demonstrated that such transformations between SQUID-MEG and OPM-MEG systems are feasible. The approach involves reconstructing neural sources from SQUID-MEG data by solving the inverse problem and then computing the corresponding magnetic fields for the OPM-MEG geometry using forward modeling. Source reconstruction was performed using the MNE algorithm and BEM framework implemented in MNE-Python (Version 0.24.1) [43,44]. For full methodological details on the dataset transformation, see [37].

### 2.3. Simulating the Magnetic Field Maps

Magnetic field maps (MFMs) were generated using MNE-Python to create subject-specific forward models and to simulate large sets of equivalent current dipoles (ECDs) [43,44]. For each subject, a cortical source space was reconstructed from individual anatomical MRI. The source grid consisted of dipole locations positioned on the outer white-matter surface, with dipole orientations constrained to be normal to the cortical surface. Each dipole was assigned a fixed strength of 10 nAm.

A total of 10,000 samples were simulated for each subject. Depending on the simulation protocol (described below), either one or two ECDs were activated per sample. The source space was defined using an octahedrally subdivided mesh, yielding approximately 8000–10,000 sources on both hemispheres combined. We implemented four simulation protocols, differing in how dipole locations were selected:

- Protocol 1 (single–all): For each sample, one ECD was selected at random from the full cortical source space.
- Protocol 2 (single–3cm): Identical to Protocol 1 but restricted to ECDs with a cortical depth of <3 cm, where depth was defined as the minimal Euclidean distance from the dipole location to the outer scalp surface.
- Protocol 3 (double–3cm): For each sample, two ECDs were selected: one from the left hemisphere and one from the right hemisphere. Only dipoles with a depth < 3 cm were allowed.
- Protocol 4 (double–auditory): For each sample, two ECDs were simulated: one in the left auditory cortex and one in the right auditory cortex. These regions were defined using the Destrieux atlas [45], specifically the labels G_temp_sup-G_T_transv-lh and G_temp_sup-G_T_transv-rh.

For each subject, the simulated ECDs were projected into the sensor space using two different forward models. First was the single-shell BEM model with a conductivity of 0.3 S/m for the inner skull surface. The individual BEM surfaces were generated from MRI using the Freesurfer watershed algorithm [46]. The second model was the homogeneous spherical conductor model (SPH). For each subject, we optimally fitted a sphere to the BEM scalp surface. Only vertices from the upper 2/3 of the scalp were used for fitting. Each set of simulated dipoles was projected through both forward models onto two sensor geometries:

- A dense grid corresponding to a hypothetical unitary OPM sensor holder (Section 2.1);
- The geometry of the SQUID-MEG system used for empirical data recording (Section 2.2).

This procedure yielded large sets of subject-specific MFMs that served as the training data for subsequent optimization of OPM sensor layouts.

### 2.4. Estimating the Optimal Layout Using the SSA

To calculate the optimal sensor layout, we use the sequential selection algorithm (SSA), developed initially for selecting optimal leads in ECG [33,47,48,49]. In our previous work, we have shown that this can be applied to SQUID-MEG and OPM-MEG [35,37]. The SSA iteratively identifies the most informative measurement channels based on their statistical relationships. At each step, channels are ranked using an information index derived from the covariance matrix. The most informative channel is selected, and the remaining covariance is updated to account for the explained variance. This process continues until the desired number of channels is chosen, enabling the estimation of unselected channels from selected ones via a linear transformation. The algorithm also tracks reconstruction error and relative statistical power, quantifying how much variance is preserved by the selected channel subset.

#### Overview of the SSA Sensor Selection Procedure

The SSA sensor selection procedure starts by constructing a data matrix X, which represents the training dataset and is composed of m magnetic field maps (MFMs), where each map contains measurements from n channels at a given time point. Accordingly, each matrix element represents a magnetic field measurement at a specific time $X_{i,k}=B_{i}(t_{k})$, with i=1,…,n denoting the channel index and k=1,…,m indexing the individual MFMs. Based on this data matrix, the covariance matrix K, and the standard deviation of the channels $\sigma_{i}$ are computed to characterize statistical dependencies between channels, and ${\bar{X}}_{i}$ denotes the average value for a specific channel over time:

(1) $$K_{ij}=\frac{1}{m}\sum_{k=1}^{m}\left(X_{i,k}-{\bar{X}}_{i}\right)\left(X_{j,k}-{\bar{X}}_{j}\right),$$

(2) $$\sigma_{i}^{2}=\frac{1}{m}\sum_{k=1}^{m}{\left(X_{i,k}-{\bar{X}}_{i}\right)}^{2}.$$

The trace of the covariance matrix ($tr\left(K\right)$) denotes the total statistical power across the whole *N*-dimensional measurement space.

Optimal channel selection is then performed using an iterative procedure in which an information index is computed for each channel j to assess its contribution to the overall data variance [33]:

(3) $$I_{j}=\sum_{i=1}^{n}K_{ij}^{2}/\sigma_{j}^{2}.$$

A channel (or a group of channels) with the highest information index is taken, and then we reorganize (interchange rows and columns) such that the values of the selected channel (or channels) are repositioned to the top left corner ($K_{ss}$):

(4) $$K=\left(\begin{array}{cc} K_{ss} & K_{su}\\ K_{us} & K_{uu} \end{array}\right).$$

From the covariance matrix of the remaining unselected channels ($K_{uu}$), we subtract the contribution from the already selected channels to calculate the covariance of the estimated error:

(5) $$K_{e}=K_{uu}-{K_{su}}^{T}{K_{ss}}^{-1}K_{su}$$

In each subsequent iteration, the information indices are recomputed for the remaining unselected channels using the updated covariance matrix $K=K_{e}$ (Equation (3)). The channel with the highest $I_{j}$ is selected, after which the original covariance matrix is reorganized as in Equation (4) to compute a new $K_{e}$. This iterative procedure is repeated until the desired number of channels is selected.

At each iteration step, we can calculate the root-mean-square error as

(6) $${\mathrm{RMS}}_{\mathrm{err}}=\sqrt{\frac{tr\left(K_{e}\right)}{n_{u}-1}}.$$

where $n_{u}$ is the number of unselected channels. The total statistic power (TSP) at each step of the SSA is defined as:

(7) $$\mathrm{TSP}=tr\left(K\right)-tr\left(K_{e}\right),$$

and the relative statistical power (RSP) as

(8) $$\mathrm{RSP}=\frac{tr\left(K\right)-tr\left(K_{e}\right)}{tr\left(K\right)}.$$

Using the repositioned K (Equation (4)), we can calculate the transformation matrix T, which we can use to estimate the magnetic values ($Y^{e}$) on unselected channels based on magnetic values for the selected channels ($Y^{s}$):

(9) $$Y^{e}=K_{\mathrm{us}}K_{ss}^{-1}Y^{s}=TY^{s}$$

**Y** denotes the evaluation (measurement) dataset rather than the training dataset. Further details on the SSA are provided in the original work by Lux et al. [33] and in our previous work [37].

### 2.5. Applying the SSA to the Simulated Data

In the related paper [37], we applied the SSA to 16 AEF datasets derived from measurements of 9 subjects. For the training dataset, we selected MFMs within the time interval [42, 240] ms, i.e., 1600 MFMs in total or 100 MFMs per measurement. In this paper, we tested several combinations of 3600 MFMs simulated data:

- Combining simulated data from all 9 subjects and all 4 protocols, i.e., 100 MFMs per subject and protocol (all–bases);
- Combining simulated data from all subjects and one protocol, i.e., 400 MFMs per subject;
- Combining simulated data from a single subject and all 4 protocols, i.e., 900 MFMs per protocol;
- Using 3600 simulated MFMs for a single subject and one protocol.

We tested all those combinations of simulated data using BEM and SPH forward models. The spatial distribution and selection order of the optimally chosen measuring sites for representative simulation configurations are illustrated in Figure 1b,c. Figure 1b shows the first 30 selected sites for the whole-head OPM grid using the all–bases SPH simulated data, while Figure 1c depicts the corresponding selection for the right-hemisphere-only configuration.

### 2.6. Evaluation Metrics

#### 2.6.1. Root Mean Square (RMS) of a Single Map

We calculated the root mean square (RMS) of a single map with a time index ${MFM}_{k}$ to obtain a value for the overall magnetic field strength across sensors:

(10) $$RMS\left(map\right)=\sqrt{\frac{1}{n}\sum_{i=1}^{n}{X_{i,k}}^{2}}.$$

#### 2.6.2. Average RMS, Average Relative Difference (RD), and Average Correlation Coefficient (CC) to Assess the SSA Optimized Layout

To evaluate the performance of the SSA-optimized sensor layouts on the evaluation dataset, three quantitative metrics were used: average RMS, average RD, and average CC. These metrics were computed by comparing the expected magnetic field values ($Y_{i,j}^{e})$ estimated using the SSA reconstruction (Equation (9)) with the corresponding measured or simulated magnetic field values ($X_{i,j}^{m}$) on the unselected channels. The average RMS is calculated as

(11) $$\mathrm{RMS}=\frac{1}{m}\sum_{j=1}^{m}\sqrt{\frac{\sum_{i=1}^{n_{u}}{\left(Y_{i,j}^{e}-Y_{i,j}^{m}\right)}^{2}}{n_{u}}}$$

and quantifies the overall magnitude of reconstruction error. $N_{u}$ denotes the number of unselected measuring sites. The average RD reflects relative amplitude differences between reconstructed and reference fields and is defined as

(12) $$RD=\frac{1}{m}\sum_{j=1}^{m}\sqrt{\frac{\sum_{i=1}^{n_{u}}{\left(Y_{i,j}^{e}-Y_{i,j}^{m}\right)}^{2}}{\sum_{i=1}^{n_{u}}{\left(Y_{i,j}^{m}\right)}^{2}}}.$$

The average CC assesses the similarity of spatial field patterns independently of absolute scaling and is defined as

(13) $$CC=\frac{1}{m}\sum_{j=1}^{m}\frac{\sum_{i=1}^{n_{u}}Y_{i,j}^{e}Y_{i,j}^{m}}{\sqrt{\sum_{i=1}^{n_{u}}{\left(Y_{i,j}^{e}\right)}^{2}{\left(Y_{i,j}^{m}\right)}^{2}}},$$

#### 2.6.3. ECD Fit and the Localization Error

To assess the effect of the optimally selected layouts on source localization accuracy, one or two equivalent current dipoles (ECDs) were fitted, using the Levenberg–Marquard non-linear least-squares method [50,51,52]. As a result, we obtained the ECD locations ${\vec{r}}_{p_{1}}$, ${\vec{r}}_{p_{2}}$ and the dipole moments $\vec{p_{1}},$ $\vec{p_{2}}$. For the forward model, we used an analytical model assuming an ECD in a homogeneous, spherically symmetric volume conductor [53]. Despite the simplifying assumptions of this model, the resulting forward solution is well suited for MEG source localization. Since the aim of this study is not to determine precise neural source locations but rather to evaluate how localization error changes as a function of the selected sensor layout relative to the whole layout, this model is sufficient. The solution is independent of the conductivity and radius of the sphere, and the radial component of the current dipole does not contribute to the magnetic field outside the sphere. The position of the sphere’s center is, however, important, as it influences the computed magnetic field. The center of the sphere was the same as for the simulations (see Section 2.2).

We fitted the dipoles to both the transformed measurements and the SSA-optimized layouts. To assess the performance of the SSA layout, we calculated the localization error (∆r), which is defined as the Euclidean distance between the source location obtained using the full set of measurement sites and that obtained using the SSA-selected subset, as well as the orientation error (Δϕ), defined as the angle between the orientations of the two equivalent current dipoles (ECDs).

## 3. Results

### 3.1. Overview of the Simulations

**Figure 2:**
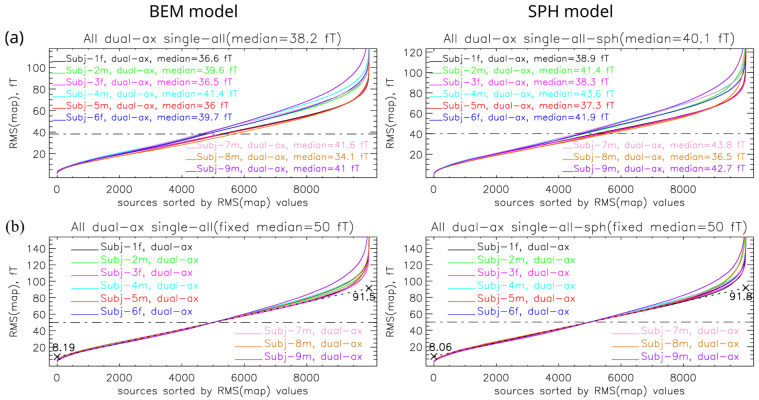
(**a**) RMS(map) values of simulated magnetic field maps for all sources in the selected database, with sources sorted in ascending order of RMS(map) for the more complex BEM and the simpler sphere forward model. For each subject, the median RMS(map) value is indicated, while the title of each plot reports the median value across all subjects, which is marked in the figure by a “−·−·” line. (**b**) Fitting of RMS(map) values using a fixed median value for all subjects. For all data points within the interval 1–6 thousand, a best-fit line was computed and shown as a dashed line. The numbers at the beginning and end of this line indicate the range of RMS values within which the majority of maps are located.

When analyzing the simulations, we observed that several simulated magnetic field maps exhibited unusually high RMS(map) values (Equation (10)), primarily because sources were located close to the sensors. Since the SSA minimizes reconstruction error, maps with higher RMS(map) values implicitly carry greater weight in optimization. To reduce this bias, the RMS(map) values of all simulated maps were computed and sorted by magnitude, as shown in Figure 2a. Based on this analysis, the median RMS(map) value for each subject was calculated and rescaled to a median of 50 fT (Figure 2b). During the SSA selection, the rescaled base is used; we selected MFMs equidistantly within the interval [30, 70] fT. In Figure 2, we show results only for simulation protocol 1 (single–all). Results for other protocols are shown in Supplementary Materials S1.

### 3.2. Different Simulation Protocols for the SSA Selection

**Figure 3:**
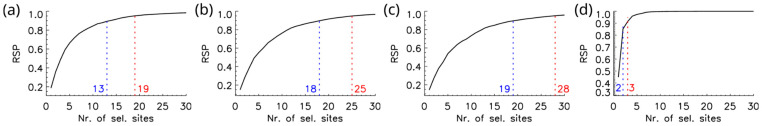
Relative statistical power (RSP) (8) vs. number of selected sites during SSA when combining all subjects and different protocols for training: (**a**) single–all, (**b**) single–3cm, (**c**) double–3cm, and (**d**) double–auditory. Blue and red dashed lines and numbers indicate at which site RSP exceeds 0.9 and 0.95 m, respectively.

Figure 3 shows the RSP (Equation (8)) as a function of the number of selected measurement sites during the SSA procedure, combining data from all subjects and using different simulation protocols. The blue and red dashed lines indicate the number of sites chosen required to exceed RSP thresholds of 0.9 and 0.95, respectively, illustrating the number of sensors needed to retain most of the statistical power under each training condition. For all four protocols, the RSP increases with the number of selected sites. The rate of increase, however, differs between training strategies; it increases most rapidly for the double–auditory protocol.

**Figure 4:**
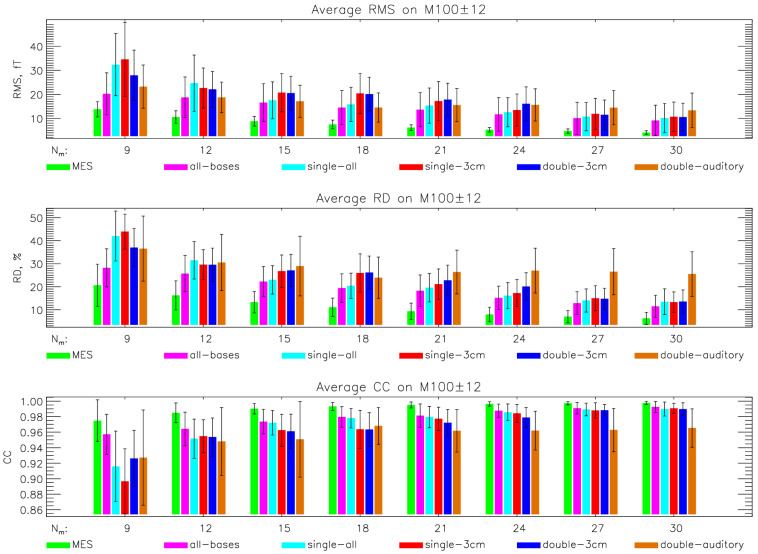
Comparison of evaluation results (RMS, RD, and CC) in the M100 ± 12 ms time interval vs. selected sites using different training bases: measured (MES), combination of all protocols and subjects (all–bases), and combination of all subjects and single protocols (single–all, single–3cm, double–3cm, and double–auditory). Data for this graph can be found in Table S1.

During SSA optimization, we calculated the average RMS (Equation (11)), RD (Equation (12)), and CC (Equation (13)) measures to evaluate how the measured dataset on different time intervals can be estimated using (9). We compared evaluation results across different SSA strategies using a measured training dataset (MES), individual simulation protocols, and a combined simulation protocol (all–bases). Figure 4 shows the results for SPH-simulated data and the time interval of M100 ± 12 ms. Results for other time intervals and the BEM forward model are in Supplementary Materials S2. These results show that the best choice for the simulated database for training is the combination of all protocols and all subjects.

**Figure 5:**
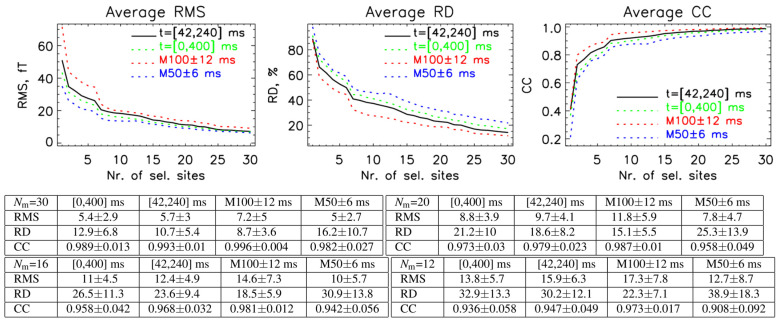
Average evaluation results on different time intervals M100 ± 12 ms, M50 ± 6 ms, [42, 240] ms, and [0, 400] ms when using a combination of all protocols and subjects (all–bases) for training. Tables report the corresponding RMS, RD, and CC values for selected sensor counts of *N_m_* = 30, 20, 16, and 12.

Figure 5 summarizes the evaluation results for four different time intervals. The metrics were computed using the training database, which combines all protocols and subjects (all–bases), and include the M100 ± 12 ms, M50 ± 6 ms, [42, 240] ms, and [0, 400] ms intervals. Across all intervals, the evaluation measures show consistent trends with increasing numbers of selected sites, indicating that the SSA-optimized layouts generalize well beyond narrow evoked-response windows. Below the figure, tables report the corresponding RMS, RD, and CC values for selected sensor counts of *N_m_* = 30, 20, 16, and 12, allowing for a direct quantitative comparison of performance across time intervals and sensor configurations.

### 3.3. Effect of Individualized Geometry on Simulation-Driven SSA

**Figure 6:**
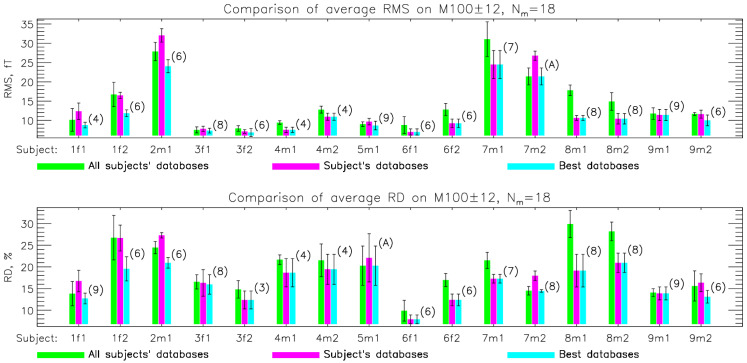

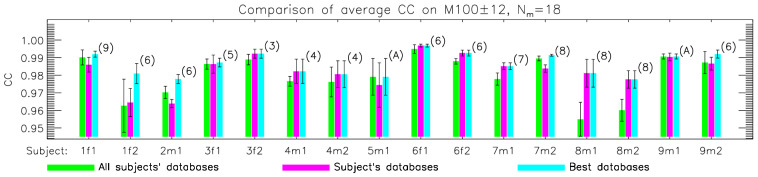
Comparison of evaluation results (RMS/RD/CC) for individual measurements in the M100 ± 12 ms time interval using 18 selected measurement sites. Results are shown as bar charts and were obtained using the SSA based on MFM simulations with the SPH forward model. Labels (A for all subjects and the number of the individual subject’s databases) inside the parentheses at the best databases’ bar indicate which database gives the best results for a given measurement, denoted by subjects’ codes on the *x*-axis (1f1, 1f2, …, 9m2). Data for this graph can be found in Table S5.

We assessed whether using individualized source geometries to generate simulated maps affects the performance of simulation-driven SSA. As shown in Figure 6, the choice of subject-specific training databases had no systematic effect on the final evaluation metrics. For individual measurements within the M100 ± 12 ms interval at 18 selected measurement sites, the RMS, RD, and CC values obtained with subject-specific simulations were comparable to those obtained with simulations pooled across all subjects. Results for other time intervals and the BEM forward model are in Supplementary Materials S3. Although for some measurements, a specific subject’s database yielded marginally better results, as indicated by the labels inside the bars, these improvements were not consistent for the subject whose data were used for training. Overall, no clear advantage was observed when matching the simulated training database to the evaluated subject, indicating that SSA performance is not dependent on individualized source geometry.

### 3.4. Localization Error for the Simulation-Driven SSA

To evaluate the impact of the simulation-driven SSA on source localization accuracy, ECD fitting was performed on measured MFMs, simulation-based SSA-estimated MFMs using the all–bases protocol, and measured data using only *N_m_* = 18 SSA-selected sites. Localization performance was assessed by comparing dipole fits obtained with SSA-selected sensor subsets to reference fits computed from all available measurement sites.

**Figure 7:**
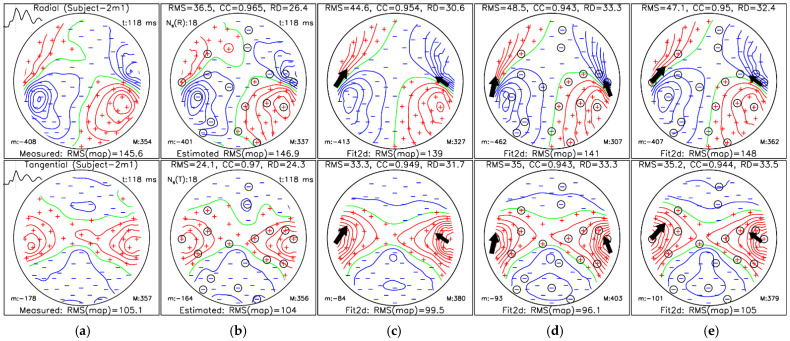
MFM at the M100 auditory evoked field peak (118 ms) for Subject-2m1: (**a**) measured reference data; (**b**) simulation-based SSA-estimated data using the “all–bases” protocol; (**c**) dual-dipole fit (Fit2d) of measured data; (**d**) dual-dipole fit of simulation-based SSA-estimated data using; and (**e**) dual-dipole fit of measured data using only *N_m_* = 18 SSA-selected sites. The radial and tangential magnetic field components are shown in the top and bottom rows, respectively. Panels (**b**–**e**) include RMS, CC, and RD relative to panel (**a**). Circled markers indicate SSA-selected sites; “m:” and “M:” denote minimum and maximum field values. Red, blue, and green isolines represent positive, negative, and zero field values, respectively. Measuring sites are denoted by red plus and blue minus signs for the corresponding positive and negative field values, respectively. Each of the dipole’s 3D positions and direction is projected into a 2D map and marked by a black arrow with the size corresponding to the dipole moment value.

**Figure 8:**
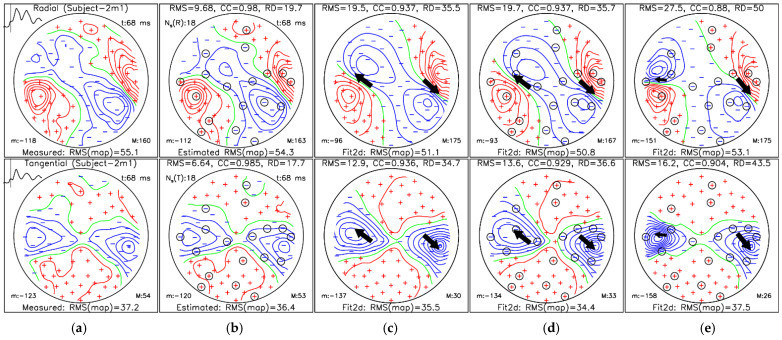
MFM at the M50 auditory evoked field peak (118 ms) for Subject-2m1: (**a**) measured reference data; (**b**) simulation-based SSA-estimated data using the “all–bases” protocol; (**c**) dual-dipole fit (Fit2d) of measured data; (**d**) dual-dipole fit of simulation-based SSA-estimated data; and (**e**) dual-dipole fit of measured data using only *N_m_* = 18 SSA-selected sites. The radial and tangential magnetic field components are shown in the top and bottom rows, respectively. Panels (**b**–**e**) include RMS, CC, and RD relative to panel (**a**). Circled markers indicate SSA-selected sites; “m:” and “M:” denote minimum and maximum field values. Red, blue, and green isolines represent positive, negative, and zero field values, respectively. Measuring sites are denoted by red plus and blue minus signs for the corresponding positive and negative field values, respectively. Each of the dipole’s 3D positions and direction is projected into a 2D map and marked by a black arrow with the size corresponding to the dipole moment value.

Figure 7 and Figure 8 show representative MFMs and dipole reconstructions at the M100 and M50 AEF peaks, respectively, for Subject-2m1. In both cases, SSA-estimated magnetic field maps closely resemble the measured reference maps, as reflected by high correlation coefficients. Dipole fits obtained from SSA-estimated maps and SSA-selected layout maps (Figure 7d,e and Figure 8d,e) yield source locations comparable to those obtained from measured data (Figure 7c and Figure 8c). For the M100 example shown in Figure 7, the ECD fit results are presented in Table 1, while the results for the M50 example shown in Figure 8 are summarized in Table 2.

**Figure 9:**
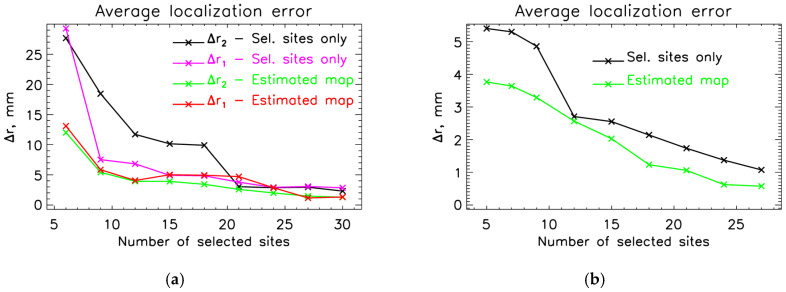
Average source localization error as a function of the number of selected measurement sites, referenced to dipole fitting using the full sensor set. Localization errors are shown for two configurations: channels from both hemispheres (**a**) and channels from the right hemisphere only (**b**). For (**a**), $\Delta r_{1}$ and $\Delta r_{2}$ correspond to localization errors obtained using only the selected measurement sites and using both the selected and SSA-estimated sites, respectively.

Figure 9 shows the average source localization error as a function of the number of selected measurement sites. As expected, localization accuracy improves as more measuring sites are included. In Figure 9a, where we use the sensors from both hemispheres, we see that for a low number of selected measurement sites (*N_m_* < 15), the localization accuracy is higher when we use SSA-estimated MFMs than when we use SSA-selected sites alone. When we use sensors from only the right hemisphere, SSA-estimated MFMs have higher accuracy across the entire shown interval.

Quantitative group-level statistics are provided in Table 3 and Table 4 for M100 MFMs. Table 3 reports average localization errors and evaluation metrics (RMS, RD, and CC) for the simulation-driven SSA using sensors from both hemispheres. We used data from 10 measurements to generate this table and Figure 9a. We excluded measurements that do not show simultaneous AEF responses in both hemispheres. Localization results for M100 for all measurements are presented in Supplementary Materials S4. In contrast, Table 4 presents the corresponding results when only right-hemisphere sensors are considered. We used data from 11 measurements to generate this table and Figure 9b. We excluded measurements that do not show a clear AEF response in the right hemisphere. Localization results for M100 on the right hemisphere for all measurements are presented in Supplementary Materials S5.

## 4. Discussion

The primary motivation of this work is to develop a methodology for optimizing the sensor layout for OPM-MEG using the SSA. This algorithm assesses relationships between measurement channels in a large database, where you have data for all possible measuring sites, to select the optimized layout. In real-life scenarios, this is not ideal, as only a few laboratories have access to 50+ OPMs to obtain a whole-head measurement. In this work, we present an approach that does not use measurements as the training database; instead, we simulate MFMs using realistic head geometries. This approach eliminates the need for pre-existing large databases.

To optimize the simulation-driven SSA methodology, we compare two forward models, simulate sources across different brain regions, and assess the impact of the number of simulated MFMs. Lastly, we perform source localization to evaluate the performance of the optimized layouts. We benchmark the simulation-driven SSA layouts with the OPM-MEG measurements of AEFs. In our work, we focus on the evoked response in the interval 40–240 ms, which is also clinically relevant [54,55,56,57,58,59]. Due to the limited number of OPMs available, we use whole-head SQUID-MEG measurements, which we transform to the unitary OPM-MEG layout [39,60].

First, we examine whether constraining simulations to a specific cortical region or employing two simultaneous ECDs yields sensor configurations tailored to a particular evoked response, such as AEFs. We compared different training databases: measured (MES, 1600 MFMs from 16 measurements on interval [42, 240] ms, 100 MFMs per measurement), combination of all subjects in simulation protocols (all–bases, 3600 MFMs, 100 MFMs per subject and protocols), and combination of all subjects and one of the protocols (single–all, single–3cm, double–3cm, and double–auditory, 400 MFMs per subject). The comparison of evaluation results on the M100 ± 12 ms interval for measured and different SPH simulated databases is display in Figure 4. Results for BEM simulated data and other time intervals are presented in Supplementary Materials S2. Comparison of the average results obtained by different simulation protocols shows the advantage of using a double-auditory training database only for the 100 ± 12 ms interval when selecting only a small number of measuring sites (up to 10).

For the nine selected measuring sites, we obtained average RMS = 23.2 ± 9.0 fT, RD = 36.5 ± 14.1%, and CC = 0.927 ± 0.061 for the SPH model (see Table S1) and RMS = 20.6 ± 8.0 fT, RD = 31.2 ± 12.5%, and CC = 0.947 ± 0.045 for the BEM model (see Table S3). This is also the only case when the BEM model clearly outperforms the SPH model. However, when using a combination of all protocols (all–bases) for the simulated training database, both models give comparable average results: RMS = 20.2 ± 8.8 fT, RD = 28.2 ± 8.3%, and CC = 0.957 ± 0.026 for the SPH model and RMS = 24.6 ± 9.6 fT, RD = 32.9 ± 9.1%, and CC = 0.943 ± 0.036 for the BEM model. The main reason why double–auditory is effective only for a small number is statistical powers (see Figure 3d, where the RSP (Equation (8)) exceeds 0.95 after 3 selected sites, and after 8–10 optimally selected sites, it reaches a plateau of 1). This means there is less variability in the database we use for learning than in other protocols, where we do not restrict simulated sources to an ROI. Therefore, all the information content of double–auditory is covered, and further site selection by the SSA is random, not optimal anymore.

These results, along with all other evaluation results across different time intervals (see summary Figures S3 and S6), suggest that all–bases is the best choice for the simulated training database. However, we still need to select more measurement sites to achieve results similar to those obtained with the measured (MES) training database. For example (see Figure S5 and Table S2), average evaluation results on interval [42, 240] ms are: RMS = 10.3 ± 2.4 fT, RD = 23.6 ± 12.5%, and CC = 0.963 ± 0.051 using MES after 12 selected sites and RMS = 12.6 ± 4.9 fT, RD = 25.1 ± 10.3%, and CC = 0.963 ± 0.037 using the all-bases SPH model after 18 selected sites. For the BEM model (Figure S8 and Table S4), we obtained RMS = 13.1 ± 5.3 fT, RD = 25.4 ± 10.6%, and CC = 0.963 ± 0.038 using all–bases after 18 selected sites. Since there was no significant difference between the evaluation results from the BEM and SPH models, we presented only the SPH results in the results section.

Using this all–bases database, we examined how the evaluation parameters (RE, CC, and RMS) change across four intervals as the number of selected channels increases (Figure 5). The lowest errors are for the M100 AEF response, as expected, since non-complex MFMs, representing a double dipole shape for this interval, are present. Despite this, for the other intervals, where we do not expect only simple MFMs, the error does not increase significantly.

Next, we check whether using a subject’s individualized head geometry when generating the simulations for the SSA is necessary/advantageous. We test this by using simulations from one subject’s head geometry (source space and forward model) and then calculate the optimized SSA layout and test it against measurements from another subject. We calculate results for all possible combinations (see Supplementary Materials S3). In Figure 6, we show how the evaluation results compare when using an individualized geometry or the SSA result from another subject, which performs best. In most cases, the best-performing SSA result was not the subject’s own. Additionally, we tested the all–bases database, which combined simulations from all subjects. This base proved to be just as effective as individualized bases (see Table S7). This base proved to be just as effective as individualized bases. Therefore, we use this base in all other calculations. The results show that there is no significant difference between using one’s individualized head geometry and using another subject’s geometry for the simulation-driven SSA selection. This is important for clinical applications. If this methodology were applied to clinical MEG systems, it would be possible to compute an optimal sensor layout and the corresponding transformation matrix (Equation (9)) using a representative set of head geometries. This approach would substantially reduce manual effort, since the calculation needs to be done only once.

Finally, we compared the usefulness of the SSA simulation approach for calculating the inverse problem. We performed ECD fits on M50 and M100 for three cases: whole-head measurements, optimally selected channels only, and sensor distribution, in which we extended the optimally selected channels to all possible locations (SSA estimation). As shown in Figure 9a, when sensors from both hemispheres are used, SSA-estimated MFMs yield lower localization errors than SSA-selected channels alone for a small number of selected sites (<15), indicating that SSA extrapolation compensates for sparse sensor coverage. When restricting sensors to the right hemisphere only (Figure 9b), SSA-estimated MFMs consistently outperform SSA-selected channels across all values of $n_{m}$, highlighting the benefit of SSA extrapolation in spatially constrained measurement scenarios. Overall, these results demonstrate that SSA-based estimation improves source localization accuracy, particularly when sensor coverage is limited.

The SSA is fundamentally a statistical channel selection method based on covariance structure rather than source localization-driven optimization. Source localization performance generally degrades as the number of sensors is reduced [5,6]; however, by accurately estimating whole-head magnetic field maps, this approach enables the extension of measured fields to unmeasured sensor locations. As shown in Figure 5, the first 15 to 20 optimally selected sensors capture most of the information (CC > 0.95) contained in full-head MFMs for the clinically relevant time interval 40–240 ms. Therefore, one can apply the SSA to calculate whole-head MFMs, which improves source localization accuracy. We demonstrate this in Figure 9 and Table 3 and Table 4, where we display localization results for M100 using one or two current dipole source models.

A limitation of the present study is that the optimization and validation of the proposed sensor layouts were primarily demonstrated using AEF data, which reflect relatively simple and spatially structured source configurations. We did not explicitly evaluate the performance of the SSA-based optimization for more complex cognitive paradigms involving distributed and time-varying sources. We expect that more complex inverse problems, such as the minimum-norm estimate (MNE) [61] and the beamformer [62], would even more benefit from well-estimated whole-head MFMs. In addition, clinically relevant applications such as focal epilepsy typically involve sources with relatively low degrees of freedom, for which we expect the proposed approach to be well suited. Systematic evaluation of these scenarios will be the focus of our future work.

Another limitation of this study is the lack of experimental evaluation using OPM sensors, largely because OPM-MEG systems with high channel counts (>50 sensors) remain relatively rare due to their high cost. Consequently, we relied on SQUID-based measurements projected onto OPM sensor locations. This process involves an intermediate inverse and forward modeling step, which necessitates some regularization. Consequently, the transformed magnetic fields are inherently “denoised” during the projection process, potentially introducing circularity that could artificially inflate source reconstruction accuracy. However, we have previously addressed the impact of this transformation [37]. We applied the SSA method to the original SQUID data and the OPM-transformed datasets, yielding comparable performance metrics across both formats. Nonetheless, it is important that this methodology is applied to and validated against real-world OPM measurements in future studies.

Furthermore, while our study utilizes synthetic field projections, real-world OPMs typically exhibit higher intrinsic noise floors than SQUIDs. Current OPM-MEG systems often rely on interference reduction techniques, such as Signal Space Separation (SSS) [63] or Homogeneous Field Correction (HFC) [64]. A critical next step is to investigate whether SSA-expanded field maps, which reconstruct high-density data from limited sensors, can be reliably integrated with these methods to mitigate noise in actual OPM recording environments. The ultimate goal of our research is to aid clinical applications. OPM-MEG enables noninvasive brain recordings with millisecond temporal resolution while allowing sensors to be placed directly on the scalp, resulting in higher signal strength, improved spatial resolution, and tolerance to head movement. These features make OPM-MEG particularly suitable for pediatric, psychiatric, and movement-disorder populations, where conventional MEG is often limited. OPM-MEG can reliably measure evoked responses, oscillatory activity, functional connectivity, and deep-brain signals with performance comparable to or exceeding SQUID-MEG. A promising future research direction is the use of MFMs for the analysis of AEFs. Analogous to body surface potential mapping in electrocardiography [65,66,67,68,69,70,71,72,73,74,75,76,77,78] and magnetic field mapping in magnetocardiography [79,80,81,82,83,84,85,86,87,88,89,90,91,92], MFMs derived from MEG recordings could enable precise visualization of spatiotemporal neural dynamics. By leveraging MFMs, it may become possible to track dynamic changes in neural activity with greater fidelity and to improve the detection and characterization of abnormal auditory processing patterns in clinical populations. Future work should investigate the integration of MFM-based analyses with SSA-optimized sensor layouts, with the aim of enhancing both the spatial resolution and interpretability of OPM-MEG measurements of auditory responses. In fact, our methodology could extend beyond neurology to prenatal care, as OPMs have proven valuable in monitoring fetal heart rate and fetal movement as objective indicators of fetal health [93,94,95,96,97,98,99,100,101,102]**.**

Beyond technical and clinical performance, the cost effectiveness of OPM-MEG remains a critical determinant of its successful clinical translation. Its economic value will ultimately depend on whether OPM-MEG can demonstrably reduce downstream healthcare costs, for example, by enabling earlier disease detection, improving surgical planning and precision, and reducing complication rates or the need for reoperations. However, several practical barriers currently limit widespread adoption. These include integration into existing clinical and surgical workflows, data acquisition and processing time, noise reduction, staff training requirements, regulatory approval pathways, and the need for standardized protocols across centers.

Addressing these challenges will require coordinated efforts in technological innovation, workflow adaptation, and rigorous health economic evaluation to establish the clinical and economic value proposition of OPM-MEG. In parallel, an additional dimension that has received limited attention until recently is patient and public involvement in research and health technology assessment [103], particularly in the development of novel imaging modalities [104]. Meaningful integration of patient perspectives across research stages, from ethics review and study design to recruitment strategies and dissemination of results, has the potential to improve research quality, align technological development with patient needs, and enhance broader societal impact.

## 5. Conclusions

We introduce a simulation-driven SSA framework for optimizing OPM-MEG sensor layouts without relying on large measurement databases. Using realistic head geometries, we simulated MFMs suitable for the SSA. The results show minimal sensitivity to forward model choice or individualized anatomy. The first 15 to 20 optimally selected sensors capture most of the information (CC > 0.95) contained in full-head MFMs for the clinically relevant time interval 40–240 ms. The SSA-optimized layouts improve source localization accuracy, particularly under sparse sensor coverage (localization error for M100 < 5 mm). This approach enables practical and scalable sensor layout optimization supporting future clinical deployment of OPM-MEG systems.

## Figures and Tables

**Table 1:** Dual ECD fitting results for the M100 AEF peak (Figure 7). The estimated dipole parameters comprise the source locations (${\vec{r}}_{1},{\vec{r}}_{2}$) and the corresponding dipole moments (${\vec{p}}_{1},{\vec{p}}_{2}$). Deviations relative to the reference solution (Figure 7c) are characterized using localization errors ($\Delta r_{1},\Delta r_{2}$), their combined Euclidean distance ($\Delta r_{c}$), and orientation discrepancies ($\Delta \phi_{1},\Delta \phi_{2}$).

Measures	Measured Map Fit (c)	Estimated Map Fit (d)	Selected Chan. Fit (e)
${\vec{r}}_{1}$ [mm]	(44.6, 0.9, 15.2)	(44.4, −4.7, 1.4)	(39.3, 1.5, 13.6)
${\vec{r}}_{2}$ [mm]	(−37.8, 3.1, 6.2)	(−37.8, −2.9, 0.6)	(−32.5, 5.3, 8.7)
${\vec{p}}_{1}$ [µAm]	(10.5, −12.0, −30.0)	(−1.3, −23.1, −36.7)	(15.4, −18.8, −42.6)
${\vec{p}}_{2}$ [µAm]	(−8.1, −26.2, −36.6)	(2.2, −37.0, −39.0)	(−19.8, −37.0, −51.9)
$∆r_{1}$ [mm]	/	14.9	5.6
$∆r_{2}$ [mm]	/	8.2	6.2
$∆r_{c}$ [mm]	/	17.0	8.3
$\Delta \phi_{1}$ [°]	/	22.3	1.9
$\Delta \phi_{2}$ [°]	/	14.8	7.1

**Table 2:** Dual ECD fitting results for the M50 AEF peak (Figure 8). The estimated dipole parameters comprise the source locations (${\vec{r}}_{1},{\vec{r}}_{2}$) and the corresponding dipole moments, including both magnitude and orientation (${\vec{p}}_{1},{\vec{p}}_{2}$). Deviations relative to the reference solution (Figure 8c) are characterized using localization errors ($\Delta r_{1},\Delta r_{2}$), their combined Euclidean distance ($\Delta r_{c}$), and orientation discrepancies ($\Delta \phi_{1},\Delta \phi_{2}$).

Measures	Measured Map Fit (c)	Estimated Map Fit (d)	Selected Chan. Fit (e)
${\vec{r}}_{1}$ [mm]	(49.7, −6.4, 27.3)	(48.9, −6.9, 25.5)	(52.4, −6.0, 24.7)
${\vec{r}}_{2}$ [mm]	(−34.0, 2.1, 41.7)	(−29.6, 0.2, 43.8)	(−57.9, 2.8, 34.5)
${\vec{p}}_{1}$ [µAm]	(−3.2, 2.9, 6.5)	(−3.3, 2.8, 7.0)	(−2.1, 3.3, 5.2)
${\vec{p}}_{2}$ [µAm]	(5.5, −3.3, 4.6)	(6.1, −4.4, 4.1)	(1.8, −0.1, 3.1)
$∆r_{1}$ [mm]	/	2.0	3.7
$∆r_{2}$ [mm]	/	5.3	25.0
$∆r_{c}$ [mm]	/	5.6	25.2
$\Delta \phi_{1}$ [°]	/	1.8	9.9
$\Delta \phi_{2}$ [°]	/	8.0	29.3

**Table 3:** Group-level average of source localization errors (mm) and evaluation parameters (RMS, RD, and CC), with standard deviations for different numbers of selected measurement sites, for simulation-driven, SSA-estimated M100 MFMs.

	Evaluation of Estimated M100			Localization—Estimated		Localization—Sel. Sites Only	
*N*_m_	RMS±SD [fT]	RD±SD [%]	CC±SD	$∆r_{1}\pm SD$ [mm]	$∆r_{2}\pm SD$ [mm]	$∆r_{1}\pm SD$ [mm]	$∆r_{2}\pm SD$ [mm]
6	34.9 ± 19.2	37.8 ± 7.5	0.926 ± 0.034	13.1 ± 5.3	12 ± 8.2	29.3 ± 11.9	27.7 ± 16.8
9	21.5 ± 11.7	24.2 ± 4.6	0.97 ± 0.01	5.8 ± 3.8	5.4 ± 2.7	7.5 ± 6.1	18.5 ± 18.8
12	20.5 ± 10.9	22.9 ± 4.4	0.973 ± 0.01	4.1 ± 1.5	3.9 ± 2.5	6.8 ± 4.3	11.7 ± 16.8
15	19.1 ± 10.6	21.1 ± 5.2	0.977 ± 0.011	5.0 ± 3.8	3.9 ± 1.7	4.9 ± 2.4	10.1 ± 16.2
18	17.5 ± 9.5	19.3 ± 5.5	0.98 ± 0.011	4.9 ± 4.4	3.4 ± 2.2	4.8 ± 3.1	9.9 ± 16.9
21	16.8 ± 9.5	18.8 ± 6.3	0.981 ± 0.013	4.7 ± 4.1	2.6 ± 1.7	3.8 ± 2.9	3.0 ± 1.2
24	14.2 ± 9.7	15.3 ± 5.3	0.988 ± 0.009	2.9 ± 3.8	2 ± 1.4	2.9 ± 2.9	2.9 ± 1.5
27	12.7 ± 9.0	13.4 ± 5.0	0.99 ± 0.007	1.2 ± 0.8	1.5 ± 1.1	3.1 ± 2.6	2.9 ± 1.6
30	11.7 ± 8.5	12.1 ± 4.9	0.992 ± 0.007	1.3 ± 1.0	1.3 ± 0.9	2.9 ± 2.7	2.3 ± 1.0

**Table 4:** Group-level average of source localization errors (mm) and evaluation parameters (RMS, RD, and CC), with standard deviations for different numbers of selected measurement sites, for simulation-driven, SSA-estimated M100 MFMs using sensors only on the right hemisphere.

	Evaluation of Estimated M100			Localization of M100	
				Estimated Map	Selected Sites Only
*N*_m_	RMS±SD [fT]	RD±SD [%]	CC±SD	∆r±SD [mm]	∆r±SD [mm]
5	32.2 ± 13.1	32.7 ± 12.1	0.944 ± 0.037	3.8 ± 1.8	5.4 ± 2.5
7	26.1 ± 11.4	25.7 ± 9.4	0.966 ± 0.021	3.6 ± 1.6	5.3 ± 3.2
9	22.5 ± 9.9	22.4 ± 10.1	0.975 ± 0.024	3.3 ± 2.5	4.9 ± 3.3
12	17.6 ± 8.5	18.2 ± 8.3	0.982 ± 0.018	2.6 ± 1.6	2.7 ± 0.8
15	16.1 ± 8.3	16.0 ± 7.5	0.985 ± 0.015	2.0 ± 1.3	2.6 ± 1.0
18	13.4 ± 9.8	12.3 ± 6.3	0.991 ± 0.010	1.2 ± 0.8	2.1 ± 0.9
21	11.2 ± 8.6	9.8 ± 5.5	0.994 ± 0.007	1.1 ± 1.1	1.7 ± 1.2
24	9.4 ± 8.2	8.3 ± 5.4	0.995 ± 0.006	0.6 ± 0.7	1.4 ± 0.9
27	9.3 ± 8.9	7.9 ± 5.8	0.995 ± 0.007	0.6 ± 0.6	1.1 ± 0.9

## Data Availability

The data presented in this study are available on request from the corresponding author. The data are not publicly available due to the nonstandard file formats.

## Supplementary Materials

The following supporting information can be downloaded at: https://www.mdpi.com/article/10.3390/s26041292/s1. Supplementary Materials S.1: Simulated databases; S.2: Comparisons of evaluation results for different databases; S.2.1: SPH model; S.2.2: BEM model; S.3: Comparison of evaluation results using personal databases; S.4: Localizations of M100 for all measurements; S.5: Localizations of M100 for all measurements using data from the right hemisphere only.

## Appendix A

Recording Details: The list of all recordings and their corresponding recording codes used throughout the paper.

**Table A1:** List of recordings.

N	Recording ^1^	Date	Age
1	Subject-1f1	8 October 2018	30
2	Subject-1f2	7 May 2019	31
3	Subject-2m1	16 October 2019	26
4	Subject-3f1	27 June 2019	31
5	Subject-3f2	10 April 2019	31
6	Subject-4m1	9 October 2018	36
7	Subject-4m2	10 May 2019	37
8	Subject-5m1	8 January 2020	31
9	Subject-6f1	5 July 2018	33
10	Subject-6f2	5 April 2019	34
11	Subject-7m1	7 May 2019	54
12	Subject-7m2	17 October 2019	54
13	Subject-8m1	11 October 2019	26
14	Subject-8m2	18 October 2019	26
15	Subject-9m1	18 June 2018	57
16	Subject-9m2	12 June 2019	58

^1^ f—female; m—male.
