# Supplementary material for: Optimizing OPM-MEG Sensor Layouts Using the Sequential Selection Algorithm with Simulated Sources and Individual Anatomy
Source: Sensors (Basel). 2026 Feb 17;26(4):1292. doi: 10.3390/s26041292 (PMC12944440; doi:10.3390/s26041292)
Supplement: Supplementary file 1 [file sensors-26-01292-s001.zip › sensors-4127895-supplementary.pdf]

S Supplementary Materials

Optimizing OPM-MEG Sensor Layouts Using the Sequential Selection Algorithm with Simulated Sources and Individual Anatomy

Urban Marhl, Rok Hren, Tilmann Sander, and Vojko Jazbinšek

Contents

S.1 Simulated databases . . . 2
S.2 Comparisons of evaluation results for different databases . . . 4
S.2.1 SPH model . . . 5
S.2.2 BEM model . . . 8
S.3 Comparison of evaluation results using personal databases . . . 11
S.4 Localization of M100 for all measurements . . . 15
S.5 Localization of M100 and for all measurements using data from the right hemisphere only . . . 24

List of Tables

S.1 Evaluation results on M100±12 ms for measured and different SPH simulated databases from Fig. S.4 (Fig. 3 in the paper). . . . 6
S.2 Evaluation results on $[42,240]$ ms for measured and different SPH simulated databases from Fig. S.5. . . . 7
S.3 Evaluation results on M100±12 ms for measured and different BEM simulated databases from Fig. S.7 . . . 9
S.4 Evaluation results on $[42,240]$ ms for measured and different BEM simulated databases from Fig. S.8. . . . 10
S.5 Evaluation results on M100±12 ms, SPH model, N_{m}=18 from Fig. S.9 (Fig. 5 in the paper) . . . 12
S.6 Evaluation results on M100±12 ms, BEM model, N_{m}=18 from Fig. S.10 . . . 13
S.7 Overview of evaluation results for single measurements using SPH and BEM simulated training databases . . . 14

List of Figures

S.1 Original simulated RMS(map) values . . . 2
S.2 Rescaled simulated RMS(map) values . . . 3
S.3 Summary of comparison of different databases for SPH model. . . . 5
S.4 (Fig. 3) Comparison of evaluation results on M100±12 ms for measured and different SPH simulated databases. . . . 6
S.5 Comparison of evaluation results on $[42,240]$ ms for measured and different SPH simulated databases . . . 7
S.6 Summary of comparison different databases for BEM model. . . . 8
S.7 Comparison of evaluation results on M100±12 ms for measured and different BEM simulated databases. . . . 9
S.8 Comparison of evaluation results on $[42,240]$ ms for measured and different BEM simulated databases ($N_{\mathrm{m}} = 9, 12, \ldots, 30$). . . . 10
S.9 (Fig. 5) Comparison of evaluation results for single measurements, SPH model, M100±12 ms, N_{m}=18 . . . 12
S.10 Comparison of evaluation results for single measurements, BEM model, M100±12 ms, N_{m}=18 . . . 13
S.11 Subject-1f1: 18 selected sites using **all-bases-sph**, fitting M100 with 2 dipoles . . . 16
S.12 Subject-1f2: 18 selected sites using **all-bases-sph**, fitting M100 with 1 dipole . . . 16
S.13 Subject-2m1: 18 selected sites using **all-bases-sph**, fitting M100 with 2 dipoles . . . 17
S.14 Subject-5m1: 18 selected sites using **all-bases-sph**, fitting M100 with 2 dipoles . . . 17
S.15 Subject-3f1: 18 selected sites using **all-bases-sph**, fitting M100 with 2 dipoles . . . 18
S.16 Subject-3f2: 18 selected sites using **all-bases-sph**, fitting M100 with 2 dipoles . . . 18
S.17 Subject-4m1: 18 selected sites using **all-bases-sph**, fitting M100 with 2 dipoles . . . 19
S.18 Subject-4m2: 18 selected sites using **all-bases-sph**, fitting M100 with 2 dipoles . . . 19
S.19 Subject-6f1: 18 selected sites using **all-bases-sph**, fitting M100 with 2 dipoles . . . 20
S.20 Subject-6f2: 18 selected sites using **all-bases-sph**, fitting M100 with 2 dipoles . . . 20
S.21 Subject-7m1: 18 selected sites using **all-bases-sph**, fitting M100 with 2 dipoles . . . 21
S.22 Subject-7m2: 18 selected sites using **all-bases-sph**, fitting M100 with 2 dipoles . . . 21
S.23 Subject-8m1: 18 selected sites using **all-bases-sph**, fitting M100 with 2 dipoles . . . 22
S.24 Subject-8m2: 18 selected sites using **all-bases-sph**, fitting M100 with 2 dipoles . . . 22
S.25 Subject-9m1: 18 selected sites using **all-bases-sph**, fitting M100 with 2 dipoles . . . 23
S.26 Subject-9m2: 18 selected sites using **all-bases-sph**, fitting M100 with 1 dipole . . . 23
S.27 Subject-1f1: 9 selected sites using **all-bases-sph**, fitting M100 with 1 dipole . . . 24
S.28 Subject-1f2: 9 selected sites using **all-bases-sph**, fitting M100 with 1 dipole . . . 24
S.29 Subject-2m1: 9 selected sites using **all-bases-sph**, fitting M100 with 1 dipole . . . 25
S.30 Subject-5m1: 9 selected sites using **all-bases-sph**, fitting M100 with 1 dipole . . . 25
S.31 Subject-3f1: 9 selected sites using **all-bases-sph**, fitting M100 with 1 dipole . . . 26
S.32 Subject-3f2: 9 selected sites using **all-bases-sph**, fitting M100 with 1 dipole . . . 26
S.33 Subject-4m1: 9 selected sites using **all-bases-sph**, fitting M100 with 1 dipole . . . 27
S.34 Subject-4m2: 9 selected sites using **all-bases-sph**, fitting M100 with 1 dipole . . . 27
S.35 Subject-6f1: 9 selected sites using **all-bases-sph**, fitting M100 with 1 dipole . . . 28
S.36 Subject-6f2: 9 selected sites using **all-bases-sph**, fitting M100 with 1 dipole . . . 28
S.37 Subject-7m1: 9 selected sites using **all-bases-sph**, fitting M100 with 1 dipole . . . 29
S.38 Subject-7m2: 9 selected sites using **all-bases-sph**, fitting M100 with 1 dipole . . . 29
S.39 Subject-8m1: 9 selected sites using **all-bases-sph**, fitting M100 with 1 dipole . . . 30
S.40 Subject-8m2: 9 selected sites using **all-bases-sph**, fitting M100 with 1 dipole . . . 30
S.41 Subject-9m1: 9 selected sites using **all-bases-sph**, fitting M100 with 1 dipole . . . 31
S.42 Subject-9m2: 9 selected sites using **all-bases-sph**, fitting M100 with 1 dipole . . . 31

S.1 Simulated databases

We implemented four simulation protocols:

- **single-all** – for each sample, one dipole was selected at random from the full cortical source space,
- **single-3cm** – single dipoles restricted to a cortical depth of < 3 cm,
- **double-3cm** – two dipoles, one from left and one from the right hemisphere, were selected with depth < 3 cm,
- **double-auditory** for each sample, two dipoles were selected, one in the left and one in the right auditory cortex.

A total of 10,000 samples were simulated for each subject and each simulation protocol.

For the forward problem, we used the individual BEM model, and a simplified spherical conductor model (SPH). For each subject, we optimally fitted a sphere to the BEM scalp surface. Results for both models are displayed in Fig. S.1.

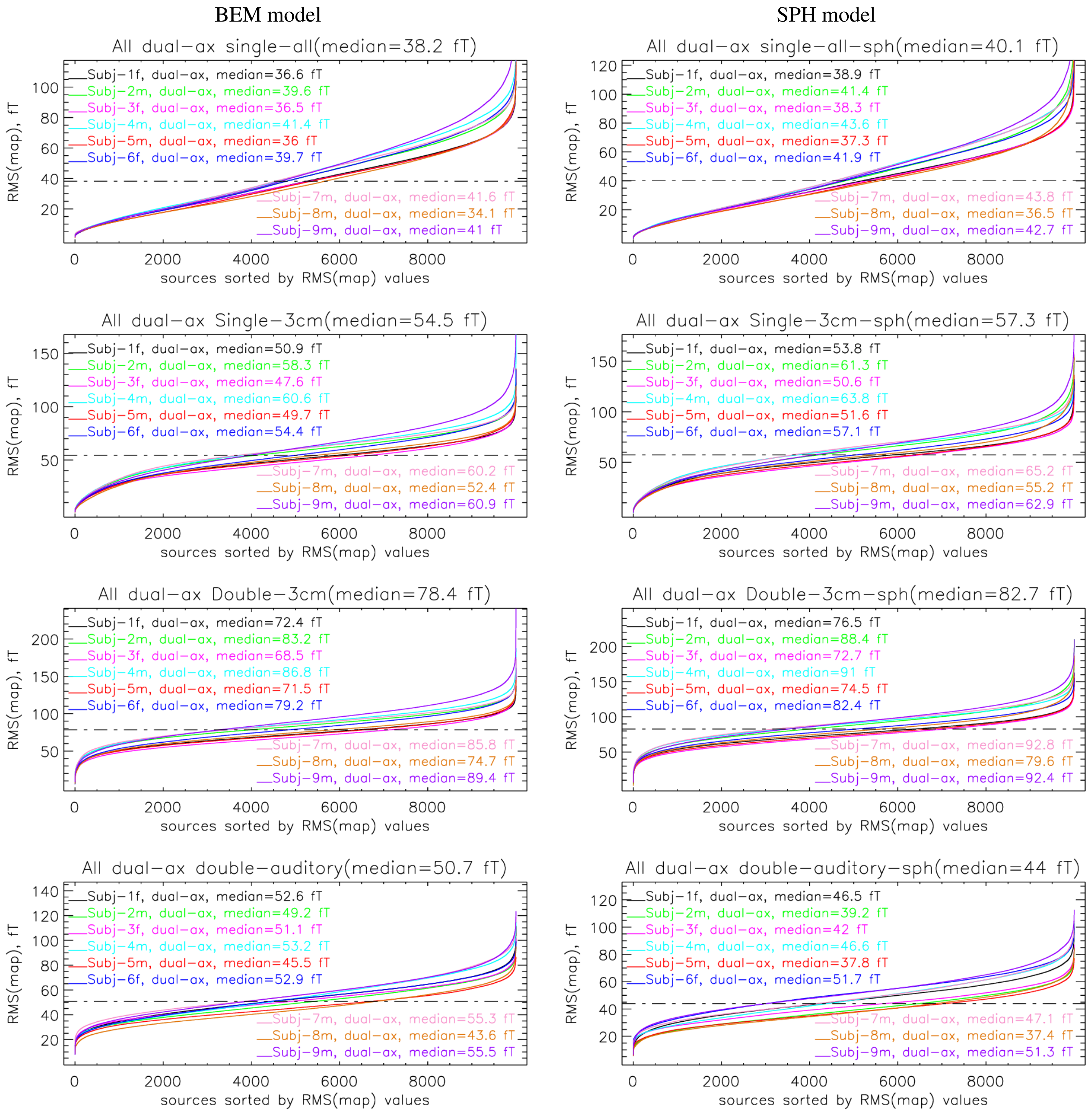

Figure S.1: RMS(map) values for all simulated databases for the more complex BEM model in the simpler SPH model, sorted by RMS(map) values. For each subject, the median RMS(map) value is indicated, while the title of each plot denotes the median value across all subjects, which is marked in the figure by a "— · —·" line.

SSA is based on minimizing RMS error of the estimated maps from limited number of selected channels. In order to combine maps from different simulations into one training database, we rescaled all simulated databases from Fig. S.1 to a median value of 50 fT. Results are displayed in Fig. S.2. We also fitted the linear part in the range from 1000 to 6000 to estimate the RMS(map) range of the most maps. In this paper, we selected the rescaled maps equidistantly from the interval [30,70] fT.

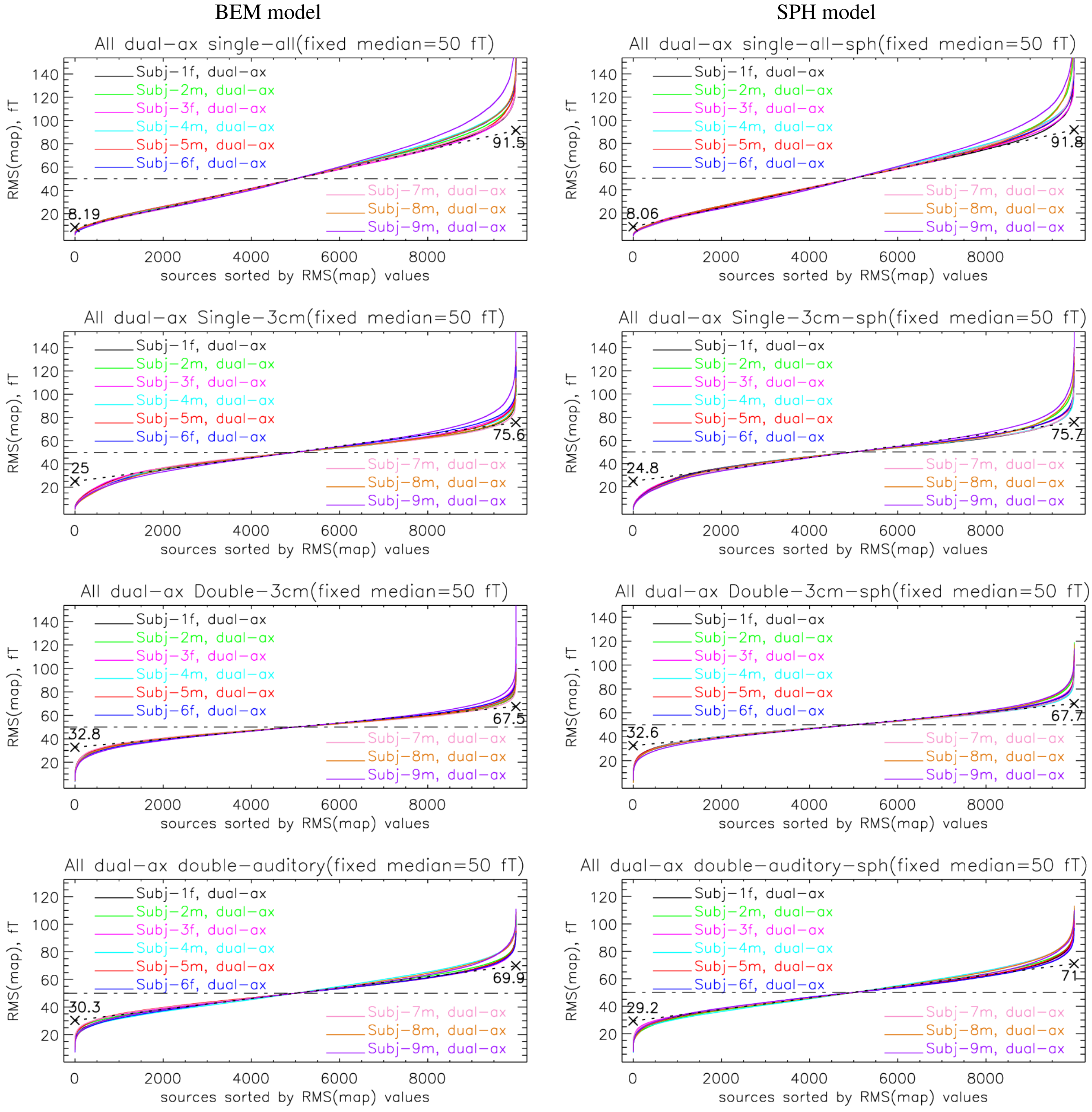

Figure S.2: Rescaled simulated RMS(map) values. The best fitting line for the RMS(map) values between 1 and 6 thousand is displayed with a dashed line. The numbers at the beginning and end of this line indicate the range of RMS values within which the majority of maps are located.

S.2 Comparisons of evaluation results for different databases

Here we compared different training databases: measured (**MES**, 1600 MFMs from 16 measurements on interval [42,240] ms, 100 MFMs per measurement), combination of all subjects in simulation protocols (**all-bases**, 3600 MFMs, 100 MFMs per subject and protocol), combination of all subjects and one of the protocols (**single-all**, **single-3cm**, **double-3cm**, **double-auditory**, 400 MFMs per subject).

In subsections S.2.1 and S.2.2 are results for the SPH and BEM models, respectively. Fig. S.3 for the SPH model and Fig. S.6 for the BEM model display a summary of evaluation results for $N_{\mathrm{m}} = 12, 18, 24, 30$ selected sites for the measured (MES) and different simulated training databases and different time intervals.

More detailed evaluation results on M100±12 ms and [42,240] ms intervals are displayed in Figs. S.4, S.5 for the SPH model, and in Figs. S.7, S.8 for the BEM model.

Comparison of average results obtained by different simulation protocols shows advantage of using **double-auditory** training database only for the 100±12 ms interval when selecting only a small number of measuring sites (up to 10). For the nine selected measuring sites we obtained average RMS=23.2±9.0 fT, RD=36.5±14.1%, CC=0.927±0.061 for the SPH model (see, Table S.1), and RMS=20.6±8.0 fT, RD=31.2±12.5%, CC=0.947±0.045 for the BEM model (see, Table S.3). This is also the only case when BEM model clearly outperforms SPH model. However, when using combination of all protocols (**all-bases**) for simulated training database both models give comparable average results: RMS=20.2±8.8 fT, RD=28.2±8.3%, CC=0.957±0.026 for the SPH model, and RMS=24.6±9.6 fT, RD=32.9±9.1%, CC=0.943±0.036 for the BEM model. The main reason, why **double-auditory** is effective only for a small number comes from the statistical power, see Fig. 2d, where the RSP (8) exceeds value of 0.95 after 3 selected sites already, and after 8-10 optimally selected sites reaches a plateau of 1. Therefore, all the information content of **double-auditory** is covered and further sites selection by SSA is random, not optimal anymore.

These results and all other evaluation results on different time interval, see summary Figs. S.3 and S.6, suggest that **all-bases** is the best choice for simulated training database. However, we still need to select more measuring sites to achieve similar evaluation results compared to results obtained measured **MES** training data base. For example (see, Fig. S.5 and Table S.2), average evaluation results on interval [42,240] ms are: RMS=10.3±2.4 fT, RD=23.6±12.5%, CC=0.963±0.051 using **MES** after 12 selected sites, and RMS=12.6±4.9 fT, RD=25.1±10.3%, CC=0.963±0.037 using **all-bases** SPH model after 18 selected sites. For the BEM model (see, Fig. S.8 and Table S.4), we obtained RMS=13.1±5.3 fT, RD=25.4±10.6%, CC=0.963±0.038 using **all-bases** after 18 selected sites.

Comparison of all the results show that both BEM and SPH forward models give very similar average RMS, RD and CC values and their standard deviations. Therefore we can conclude that using the more complex only numerically solvable BEM model has no advantage over the simplified analytically solvable SPH model for simulated data calculations.

S.2.1 SPH model

Fig. S.3 displays a summary of evaluation results for $N_\mathrm{m} = 12, 18, 24, 30$ selected sites for the measured (MES) and different SPH simulated training databases and different time intervals.
More detailed evaluation results for $N_\mathrm{m} = 9, 12, \ldots, 30$ selected sites are displayed in Fig. S.4 for the M100±12 ms, and in Fig. S.5 for the [42,240] ms.

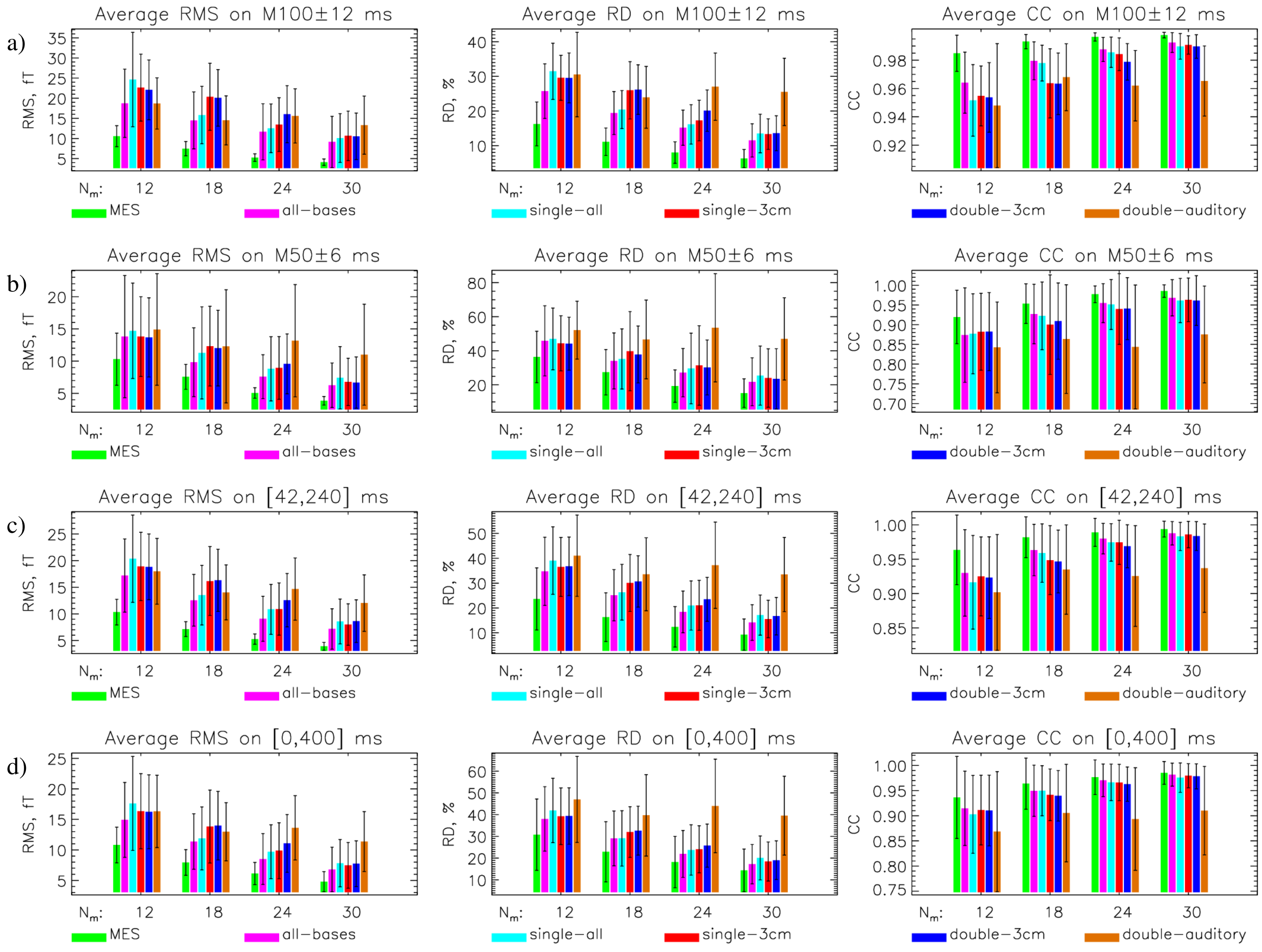

Figure S.3: Summary of comparison of evaluation results on different time intervals for the measured and different SPH simulated databases (all-bases, single-all, single-3cm, double-3cm, and double-auditory) on different time intervals: a) M100±12 ms, b) M50±6 ms, c) [42,240] ms, and d) [0,400] ms. Results for $N_\mathrm{m} = 12, 18, 24, 30$ selected sites are displayed.

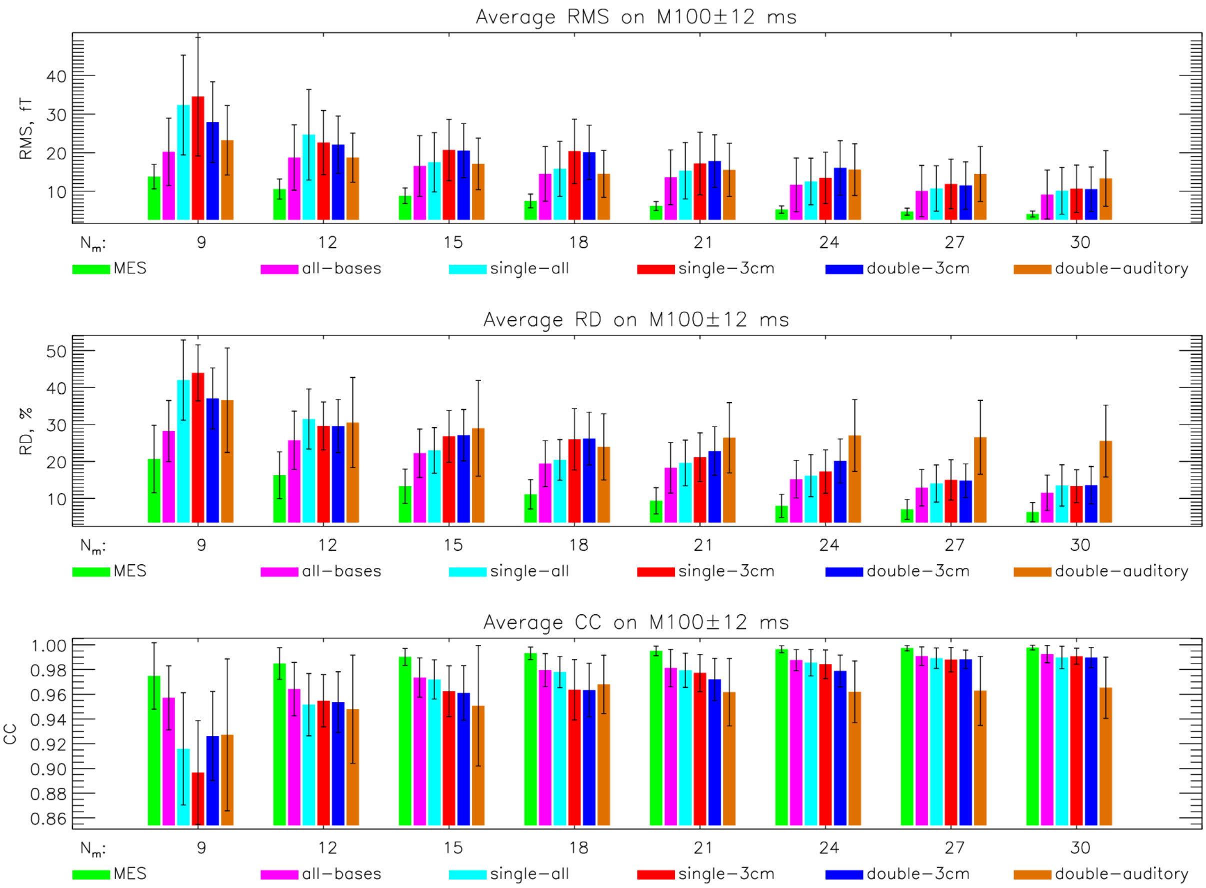

Figure S.4: (Fig.3) Comparison of evaluation results on M100±12 ms for measured and different SPH simulated databases ($N_\mathrm{m} = 9, 12, \ldots, 30$).

Table S.1: Evaluation results on M100±12 ms for measured and different SPH simulated databases from Fig. S.4 (Fig.3 in the paper).

	MES	all-bases	single-all	single-3cm	double-3cm	double-auditory
N_m	RMS ± SD	RMS ± SD	RMS ± SD	RMS ± SD	RMS ± SD	RMS ± SD
9	13.8 ± 3.16	20.2 ± 8.75	32.3 ± 12.9	34.5 ± 15.4	27.9 ± 10.5	23.2 ± 8.99
12	10.6 ± 2.6	18.7 ± 8.48	24.6 ± 11.7	22.6 ± 8.33	22.1 ± 7.44	18.7 ± 6.36
15	8.81 ± 2.03	16.5 ± 7.86	17.5 ± 7.68	20.7 ± 7.97	20.5 ± 7.01	17.1 ± 6.69
18	7.47 ± 1.77	14.5 ± 7.09	15.8 ± 7.13	20.4 ± 8.33	20.1 ± 7.03	14.5 ± 6.08
21	6.14 ± 1.17	13.6 ± 7.1	15.3 ± 7.31	17.2 ± 8.11	17.8 ± 6.84	15.5 ± 6.89
24	5.24 ± 0.948	11.7 ± 6.97	12.5 ± 6.05	13.4 ± 6.68	16 ± 7.08	15.6 ± 6.72
27	4.69 ± 0.914	10.1 ± 6.66	10.7 ± 5.89	11.9 ± 6.44	11.5 ± 6.16	14.5 ± 7.13
30	4.09 ± 0.792	9.14 ± 6.36	10.1 ± 6.07	10.7 ± 6.13	10.5 ± 5.78	13.3 ± 7.22
N_m	RD ± SD	RD ± SD	RD ± SD	RD ± SD	RD ± SD	RD ± SD
9	20.6 ± 9.13	28.2 ± 8.26	42 ± 10.8	44 ± 7.57	37 ± 8.28	36.5 ± 14.1
12	16.3 ± 6.33	25.7 ± 7.88	31.5 ± 8.12	29.6 ± 6.5	29.6 ± 7.19	30.5 ± 12.2
15	13.3 ± 4.63	22.2 ± 6.56	23 ± 6.17	26.8 ± 7	27.1 ± 6.96	29 ± 12.9
18	11.1 ± 3.97	19.4 ± 6.21	20.4 ± 5.49	26 ± 8.28	26.2 ± 7.17	23.9 ± 8.94
21	9.35 ± 3.56	18.3 ± 6.85	19.6 ± 6.19	21.1 ± 6.59	22.8 ± 6.57	26.4 ± 9.52
24	7.99 ± 3.11	15.2 ± 5.08	16.1 ± 5.69	17.3 ± 5.87	20.1 ± 5.97	27 ± 9.72
27	6.99 ± 2.7	12.9 ± 4.94	14 ± 5.03	15 ± 5.45	14.8 ± 4.55	26.5 ± 9.98
30	6.27 ± 2.61	11.5 ± 4.8	13.5 ± 5.59	13.3 ± 4.43	13.6 ± 5.04	25.5 ± 9.72
N_m	CC ± SD	CC ± SD	CC ± SD	CC ± SD	CC ± SD	CC ± SD
9	0.975 ± 0.027	0.957 ± 0.026	0.916 ± 0.045	0.897 ± 0.042	0.926 ± 0.036	0.927 ± 0.061
12	0.985 ± 0.013	0.964 ± 0.022	0.952 ± 0.025	0.955 ± 0.021	0.954 ± 0.025	0.948 ± 0.044
15	0.99 ± 0.0068	0.974 ± 0.016	0.972 ± 0.016	0.962 ± 0.021	0.961 ± 0.022	0.951 ± 0.049
18	0.993 ± 0.0051	0.98 ± 0.013	0.978 ± 0.013	0.964 ± 0.024	0.963 ± 0.022	0.968 ± 0.024
21	0.995 ± 0.0039	0.981 ± 0.015	0.979 ± 0.014	0.977 ± 0.015	0.972 ± 0.017	0.962 ± 0.027
24	0.996 ± 0.0029	0.988 ± 0.0085	0.986 ± 0.011	0.984 ± 0.012	0.979 ± 0.013	0.962 ± 0.025
27	0.997 ± 0.0022	0.991 ± 0.0074	0.989 ± 0.0083	0.988 ± 0.0099	0.988 ± 0.0074	0.963 ± 0.028
30	0.998 ± 0.0019	0.992 ± 0.007	0.99 ± 0.009	0.991 ± 0.0065	0.99 ± 0.0083	0.965 ± 0.025

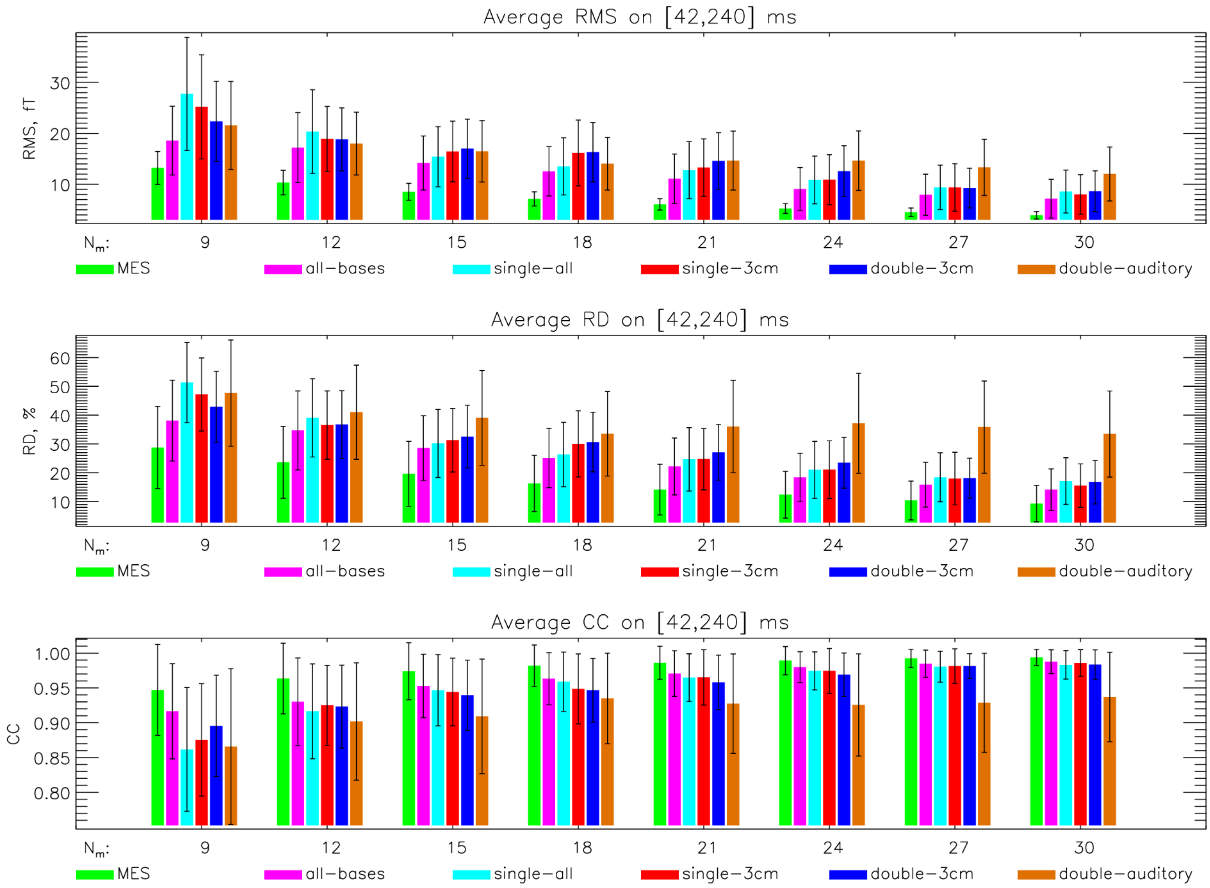

Figure S.5: Comparison of evaluation results on [42,240] ms for measured and different SPH simulated databases ($N_m = 9, 12, \ldots, 30$).

Table. S.2: Evaluation results on [42,240] ms for measured and different SPH simulated databases from Fig. S.5.

	MES	all-bases	single-all	single-3cm	double-3cm	double-auditory
N_m	RMS ± SD	RMS ± SD	RMS ± SD	RMS ± SD	RMS ± SD	RMS ± SD
9	13.2 ± 3.25	18.6 ± 6.74	27.8 ± 11.1	25.2 ± 10.2	22.3 ± 7.87	21.6 ± 8.64
12	10.3 ± 2.4	17.2 ± 6.87	20.4 ± 8.2	18.9 ± 6.4	18.8 ± 6.17	18 ± 6.17
15	8.52 ± 1.67	14.2 ± 5.3	15.4 ± 5.9	16.4 ± 5.97	17 ± 5.8	16.5 ± 6.03
18	7.14 ± 1.39	12.6 ± 4.85	13.5 ± 5.6	16.2 ± 6.47	16.3 ± 5.83	14 ± 5.16
21	6.05 ± 1.12	11.1 ± 4.85	12.8 ± 5.61	13.3 ± 5.66	14.6 ± 5.58	14.7 ± 5.8
24	5.25 ± 0.96	9.09 ± 4.22	10.9 ± 4.69	10.9 ± 4.91	12.6 ± 5.01	14.7 ± 5.83
27	4.51 ± 0.844	7.94 ± 4.04	9.4 ± 4.36	9.39 ± 4.67	9.26 ± 3.88	13.3 ± 5.51
30	3.91 ± 0.736	7.17 ± 3.8	8.58 ± 4.21	8 ± 3.89	8.64 ± 4.01	12 ± 5.3
N_m	RD ± SD	RD ± SD	RD ± SD	RD ± SD	RD ± SD	RD ± SD
9	28.8 ± 14.2	38.1 ± 14	51.3 ± 13.9	47.2 ± 12.7	43 ± 12.3	47.7 ± 18.4
12	23.6 ± 12.5	34.7 ± 13.7	39.1 ± 13.6	36.5 ± 11.9	36.8 ± 11.7	41 ± 16.3
15	19.6 ± 11.3	28.6 ± 11.2	30.2 ± 11.8	31.3 ± 11	32.6 ± 10.9	39 ± 16.5
18	16.3 ± 9.76	25.1 ± 10.3	26.3 ± 11.2	30 ± 11.5	30.7 ± 10.3	33.6 ± 14.7
21	14.2 ± 8.8	22.2 ± 9.88	24.7 ± 11	24.8 ± 10.6	27.1 ± 9.68	36.1 ± 16
24	12.4 ± 8.13	18.4 ± 8.38	21 ± 9.88	21.1 ± 10	23.5 ± 8.81	37.2 ± 17.4
27	10.4 ± 6.73	15.9 ± 7.79	18.5 ± 8.5	18 ± 9.14	18.1 ± 6.93	35.9 ± 16
30	9.29 ± 6.3	14.2 ± 7.17	17.1 ± 8.09	15.6 ± 7.54	16.7 ± 7.54	33.5 ± 14.9
N_m	CC ± SD	CC ± SD	CC ± SD	CC ± SD	CC ± SD	CC ± SD
9	0.947 ± 0.065	0.916 ± 0.068	0.862 ± 0.089	0.875 ± 0.081	0.895 ± 0.073	0.866 ± 0.112
12	0.963 ± 0.051	0.93 ± 0.063	0.916 ± 0.068	0.925 ± 0.057	0.923 ± 0.059	0.902 ± 0.084
15	0.974 ± 0.041	0.953 ± 0.046	0.947 ± 0.051	0.944 ± 0.049	0.939 ± 0.05	0.909 ± 0.082
18	0.982 ± 0.03	0.963 ± 0.037	0.959 ± 0.043	0.949 ± 0.05	0.947 ± 0.046	0.935 ± 0.065
21	0.986 ± 0.024	0.971 ± 0.033	0.965 ± 0.034	0.965 ± 0.04	0.958 ± 0.039	0.927 ± 0.071
24	0.989 ± 0.02	0.98 ± 0.022	0.974 ± 0.027	0.974 ± 0.032	0.969 ± 0.031	0.926 ± 0.073
27	0.992 ± 0.013	0.985 ± 0.02	0.98 ± 0.022	0.981 ± 0.025	0.981 ± 0.018	0.929 ± 0.071
30	0.994 ± 0.012	0.988 ± 0.017	0.983 ± 0.02	0.986 ± 0.019	0.984 ± 0.021	0.937 ± 0.064

S.2.2 BEM model

Fig. S.6 display a summary of evaluation results for $N_{\mathrm{m}} = 12, 18, 24, 30$ selected sites for the measured (MES) and different BEM simulated training databases and different time intervals.
More detailed evaluation results for $N_{\mathrm{m}} = 9, 12, \ldots, 30$ selected sites are displayed in Fig. S.7 for the M100±12 ms, and in Fig. S.8 for the [42,240] ms.

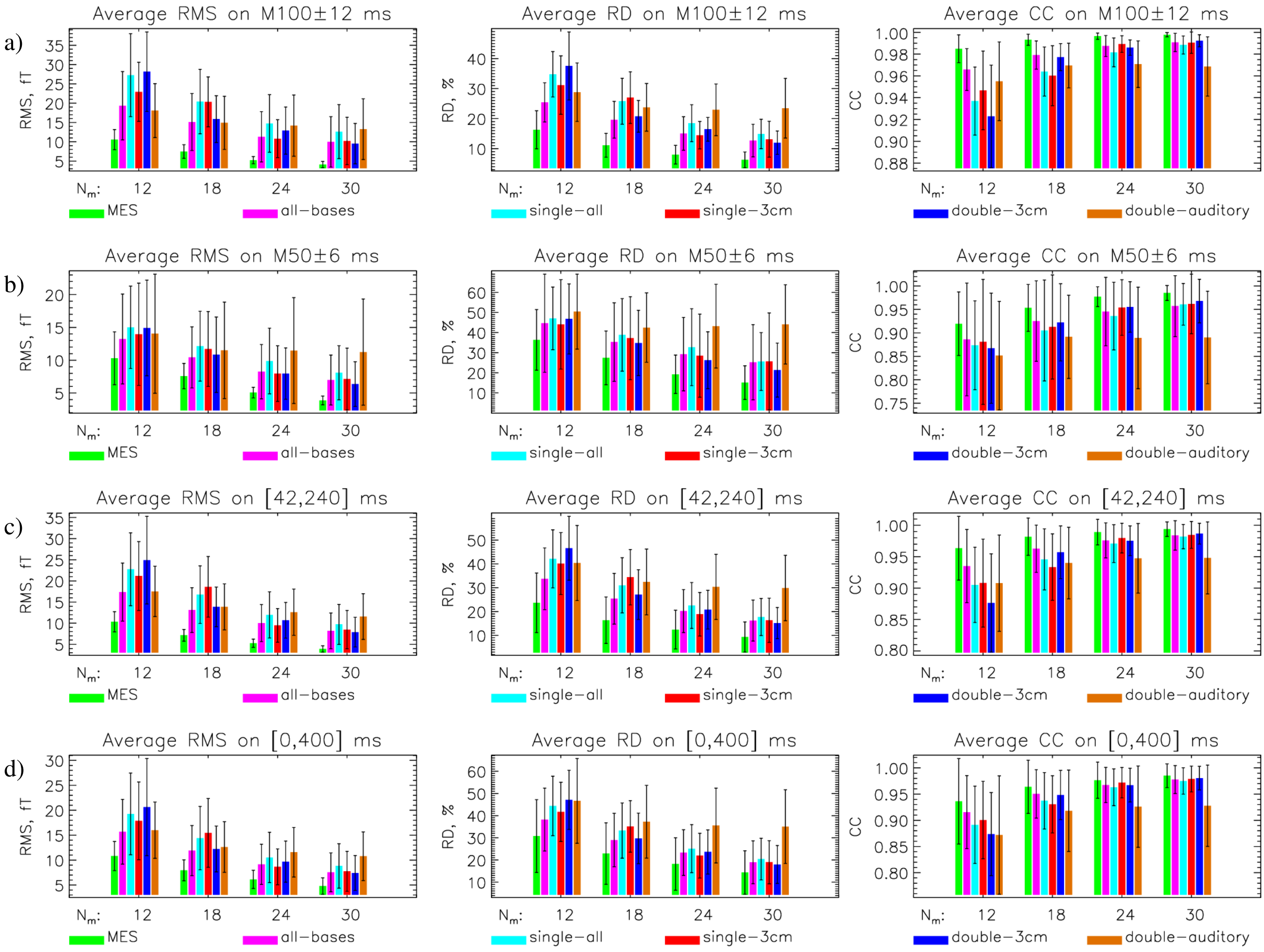

Figure S.6: Summary of comparison of evaluation results on different time intervals for measured and different BEM simulated databases ($N_{\mathrm{m}} = 12, 18, 24, 30$) on different time intervals: a) M100±12 ms, b) M50±6 ms, c) [42,240] ms, and d) [0,400] ms.

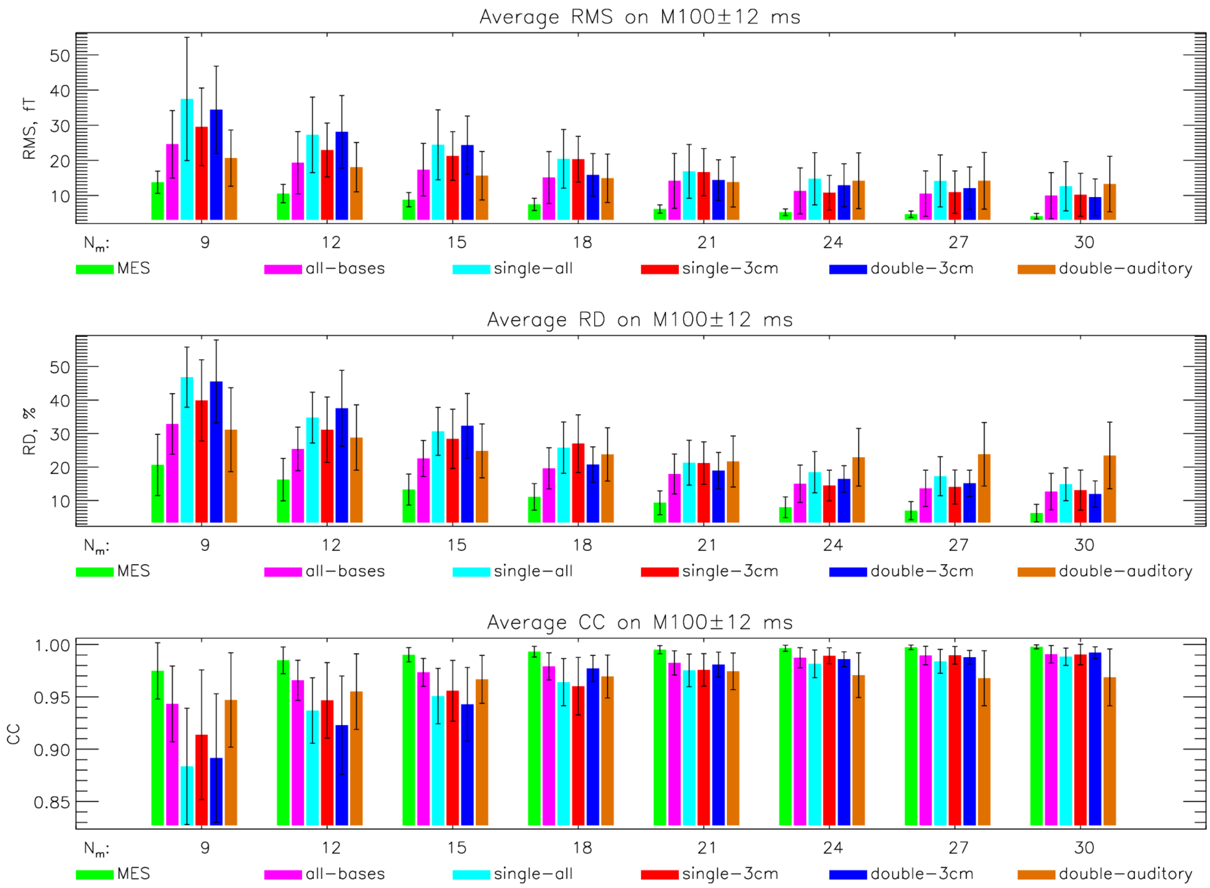

Figure S.7: Comparison of evaluation results on M100±12 ms for measured and different BEM simulated databases (N_m = 9, 12, ..., 30) Table S.3: Evaluation results on M100±12 ms for measured and different BEM simulated databases from Fig. S.7

	MES	all-bases	single-all	single-3cm	double-3cm	double-auditory
N_m	RMS ± SD	RMS ± SD	RMS ± SD	RMS ± SD	RMS ± SD	RMS ± SD
9	13.8 ± 3.16	24.6 ± 9.59	37.5 ± 17.5	29.6 ± 11	34.4 ± 12.4	20.6 ± 7.97
12	10.6 ± 2.6	19.3 ± 8.85	27.3 ± 10.7	22.9 ± 7.65	28.2 ± 10.3	18.1 ± 7
15	8.81 ± 2.03	17.3 ± 7.46	24.4 ± 9.96	21.3 ± 6.91	24.4 ± 8.28	15.6 ± 6.91
18	7.47 ± 1.77	15.1 ± 7.39	20.4 ± 8.34	20.3 ± 6.48	15.9 ± 6.06	14.9 ± 6.89
21	6.14 ± 1.17	14.2 ± 7.81	16.9 ± 7.66	16.6 ± 6.71	14.4 ± 5.78	13.8 ± 7.11
24	5.24 ± 0.948	11.3 ± 6.53	14.7 ± 7.45	10.8 ± 4.92	12.9 ± 6.11	14.2 ± 7.93
27	4.69 ± 0.914	10.5 ± 6.45	14.1 ± 7.39	11 ± 6.05	12.1 ± 6.05	14.2 ± 8.05
30	4.09 ± 0.792	9.98 ± 6.54	12.6 ± 7	10.2 ± 6.12	9.51 ± 5.23	13.3 ± 7.88
N_m	RD ± SD	RD ± SD	RD ± SD	RD ± SD	RD ± SD	RD ± SD
9	20.6 ± 9.13	32.9 ± 9.06	46.8 ± 8.96	39.9 ± 12.1	45.5 ± 12.4	31.2 ± 12.5
12	16.3 ± 6.33	25.4 ± 6.51	34.8 ± 7.56	31.1 ± 9.75	37.5 ± 11.3	28.8 ± 9.74
15	13.3 ± 4.63	22.6 ± 5.37	30.7 ± 7.16	28.4 ± 8.84	32.3 ± 9.62	24.8 ± 8.06
18	11.1 ± 3.97	19.6 ± 6.13	25.8 ± 7.65	27 ± 8.6	20.8 ± 5.28	23.7 ± 7.96
21	9.35 ± 3.56	17.9 ± 5.95	21.3 ± 6.69	21.2 ± 6.34	18.9 ± 5.44	21.7 ± 7.6
24	7.99 ± 3.11	15 ± 5.58	18.5 ± 6.15	14.5 ± 4.56	16.4 ± 3.96	22.9 ± 8.6
27	6.99 ± 2.7	13.6 ± 5.41	17.3 ± 5.8	14.1 ± 5.08	15.1 ± 3.91	23.8 ± 9.47
30	6.27 ± 2.61	12.7 ± 5.43	14.9 ± 4.88	13.1 ± 5.96	12 ± 3.9	23.5 ± 9.94
N_m	CC ± SD	CC ± SD	CC ± SD	CC ± SD	CC ± SD	CC ± SD
9	0.975 ± 0.027	0.943 ± 0.036	0.884 ± 0.056	0.914 ± 0.062	0.892 ± 0.061	0.947 ± 0.045
12	0.985 ± 0.013	0.966 ± 0.019	0.937 ± 0.031	0.947 ± 0.036	0.923 ± 0.047	0.955 ± 0.036
15	0.99 ± 0.0068	0.973 ± 0.013	0.951 ± 0.026	0.956 ± 0.029	0.943 ± 0.035	0.967 ± 0.023
18	0.993 ± 0.0051	0.979 ± 0.013	0.964 ± 0.022	0.96 ± 0.027	0.977 ± 0.012	0.969 ± 0.02
21	0.995 ± 0.0039	0.982 ± 0.012	0.975 ± 0.016	0.976 ± 0.016	0.981 ± 0.012	0.974 ± 0.017
24	0.996 ± 0.0029	0.987 ± 0.0096	0.982 ± 0.013	0.989 ± 0.0076	0.986 ± 0.0071	0.971 ± 0.021
27	0.997 ± 0.0022	0.989 ± 0.0088	0.984 ± 0.012	0.99 ± 0.0084	0.988 ± 0.0065	0.968 ± 0.026
30	0.998 ± 0.0019	0.991 ± 0.0084	0.988 ± 0.0082	0.991 ± 0.0098	0.992 ± 0.0056	0.969 ± 0.027

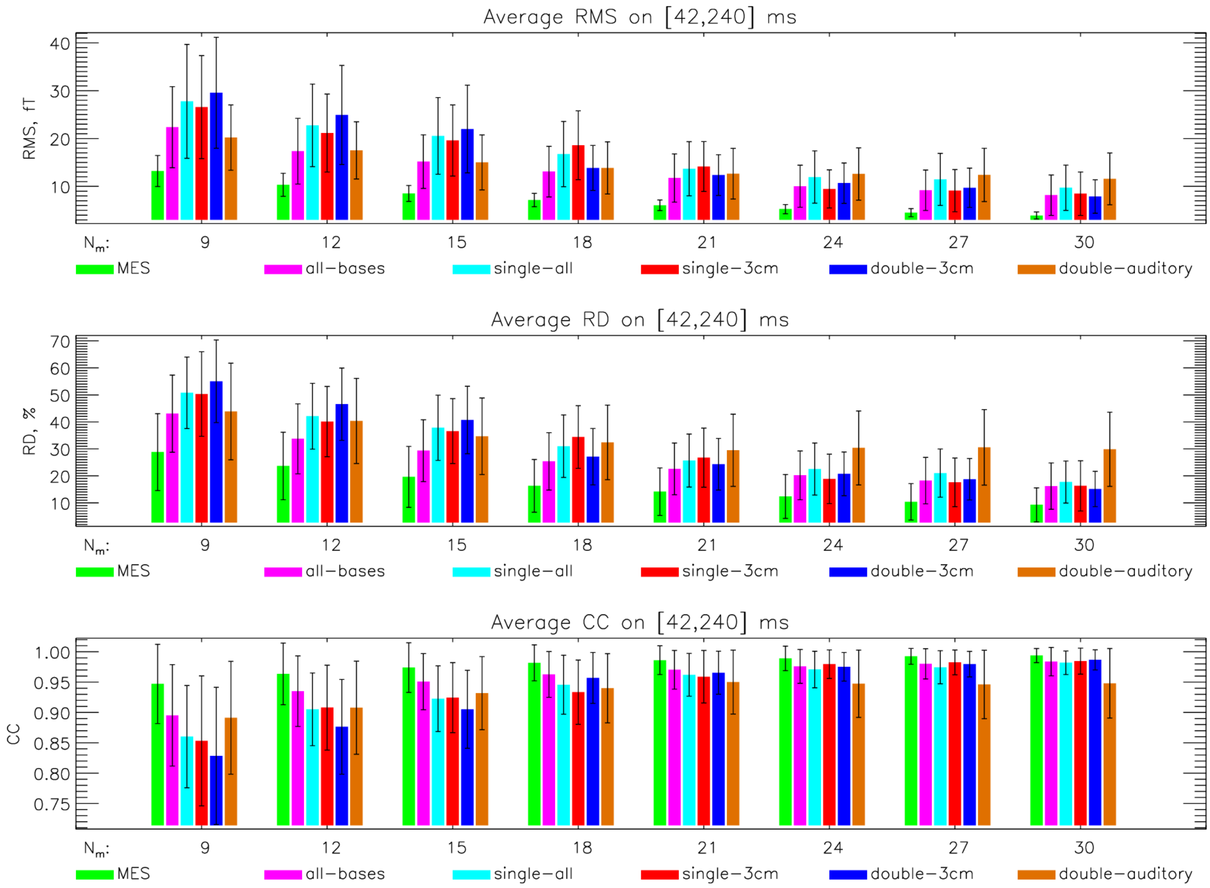

Figure S.8: Comparison of evaluation results on [42, 240] ms for measured and different BEM simulated databases (N_m = 9, 12, . . . , 30). Table S.4: Evaluation results on [42, 240] ms for measured and different BEM simulated databases from Fig. S.8.

	MES	all-bases	single-all	single-3cm	double-3cm	double-auditory
N_m	RMS ± SD	RMS ± SD	RMS ± SD	RMS ± SD	RMS ± SD	RMS ± SD
9	13.2 ± 3.25	22.4 ± 8.48	27.8 ± 11.9	26.6 ± 10.8	29.6 ± 11.6	20.2 ± 6.83
12	10.3 ± 2.4	17.4 ± 6.87	22.8 ± 8.63	21.2 ± 8.15	24.9 ± 10.4	17.5 ± 5.99
15	8.52 ± 1.67	15.2 ± 5.6	20.6 ± 8.01	19.6 ± 7.43	22 ± 9.16	15 ± 5.74
18	7.14 ± 1.39	13.1 ± 5.29	16.7 ± 6.81	18.6 ± 7.19	13.9 ± 4.71	13.9 ± 5.45
21	6.05 ± 1.12	11.8 ± 5.04	13.7 ± 5.66	14.2 ± 5.22	12.4 ± 4.26	12.7 ± 5.29
24	5.25 ± 0.96	10 ± 4.39	12 ± 5.47	9.47 ± 3.98	10.7 ± 4.23	12.6 ± 5.47
27	4.51 ± 0.844	9.2 ± 4.23	11.5 ± 5.45	9.11 ± 4.4	9.71 ± 4.11	12.4 ± 5.57
30	3.91 ± 0.736	8.17 ± 4.23	9.72 ± 4.73	8.47 ± 4.54	7.88 ± 3.51	11.6 ± 5.41
N_m	RD ± SD	RD ± SD	RD ± SD	RD ± SD	RD ± SD	RD ± SD
9	28.8 ± 14.2	43 ± 14.3	50.8 ± 13.2	50.3 ± 15.7	55 ± 15.3	43.8 ± 17.9
12	23.6 ± 12.5	33.7 ± 13	42.1 ± 12.2	40.1 ± 13	46.5 ± 13.4	40.3 ± 15.8
15	19.6 ± 11.3	29.3 ± 11.4	37.8 ± 12.1	36.6 ± 12	40.7 ± 12.5	34.7 ± 14.2
18	16.3 ± 9.76	25.4 ± 10.6	31 ± 11.6	34.4 ± 11.6	27.1 ± 10.4	32.4 ± 13.8
21	14.2 ± 8.8	22.6 ± 9.58	25.7 ± 9.82	26.7 ± 11	24.3 ± 9.58	29.5 ± 13.4
24	12.4 ± 8.13	20.2 ± 9.04	22.5 ± 9.63	18.9 ± 9.15	20.8 ± 8.11	30.3 ± 13.7
27	10.4 ± 6.73	18.3 ± 8.59	21 ± 8.92	17.6 ± 9.02	18.8 ± 7.66	30.6 ± 14
30	9.29 ± 6.3	16.2 ± 8.59	17.7 ± 7.8	16.3 ± 9.29	15.1 ± 6.54	29.9 ± 13.7
N_m	CC ± SD	CC ± SD	CC ± SD	CC ± SD	CC ± SD	CC ± SD
9	0.947 ± 0.065	0.895 ± 0.084	0.86 ± 0.084	0.853 ± 0.107	0.828 ± 0.113	0.891 ± 0.093
12	0.963 ± 0.051	0.935 ± 0.058	0.905 ± 0.06	0.908 ± 0.07	0.876 ± 0.078	0.908 ± 0.077
15	0.974 ± 0.041	0.951 ± 0.046	0.923 ± 0.054	0.924 ± 0.058	0.905 ± 0.064	0.932 ± 0.06
18	0.982 ± 0.03	0.963 ± 0.038	0.946 ± 0.049	0.933 ± 0.053	0.957 ± 0.042	0.94 ± 0.057
21	0.986 ± 0.024	0.97 ± 0.032	0.962 ± 0.035	0.959 ± 0.043	0.966 ± 0.036	0.95 ± 0.053
24	0.989 ± 0.02	0.976 ± 0.028	0.971 ± 0.03	0.98 ± 0.024	0.975 ± 0.024	0.947 ± 0.055
27	0.992 ± 0.013	0.98 ± 0.025	0.975 ± 0.027	0.982 ± 0.02	0.98 ± 0.021	0.946 ± 0.056
30	0.994 ± 0.012	0.984 ± 0.023	0.982 ± 0.019	0.985 ± 0.021	0.987 ± 0.017	0.948 ± 0.057

S.3 Comparison of evaluation results using personal databases

Here we compared databases simulated from individual cortex source space.
We performed the following analysis:

1. We applied SSA using 3600 simulated training maps for each subject combining all four source spaces (**single-all**, **single-3cm**, **double-3cm**, and **double-auditory**, i.e., 900 maps per source space).
2. For comparison, we applied SSA using 3600 simulated training maps combining all four source spaces from all nine subjects, i.e., 100 maps per source space and subject (**all-bases**).
3. Simulated maps for each source space are calculated using both BEM and SPH models and rescaled to have median RMS(map) value equal 50 fT. Then training maps are selected equidistantly from the rescaled RMS(map) values in the interval [30,70] fT.
4. Using above training databases, we applied SSA and selected 12, 18, 24 and 30 measuring sites, and evaluate results using 16 measured data for different time intervals: M100±12, M50±6, [42,24], and [0,400] ms.
5. All evaluation results for each measurement are compared and stored. Figs. S.9, S.10 display example bar-charts of evaluation results on the time interval M100±12 ms after selecting N_m=18 sites for SPH and BEM model, respectively. On each of these bar-charts, we compared evaluation results (RMS±SD, RD±SD, CC±SD) for each measurement
   (a) evaluation results from all subjects' databases (**all-bases**)
   (b) evaluation results from the individual subject's databases
   (c) the best evaluation result for a given measurement, picked from all the results obtained by all subjects' databases and nine individual subject's databases
6. Results can be displayed as a table (see, Table S.5 for the results from Fig. S.9, and Table S.6 for the results from S.10). In the best results' columns we marked, which training database gives the best results for a given measurement.
7. In the last line of each table marked by Σ, the mean results averaged over all subjects are displayed.
8. All the mean evaluation results for different time intervals and both SPH and BEM models are collected in an overview table S.7

Evaluation results clearly show that there is no advantage using individual databases or using an individual BEM model instead simplified analytically solvable SPH model to simulate training databases.

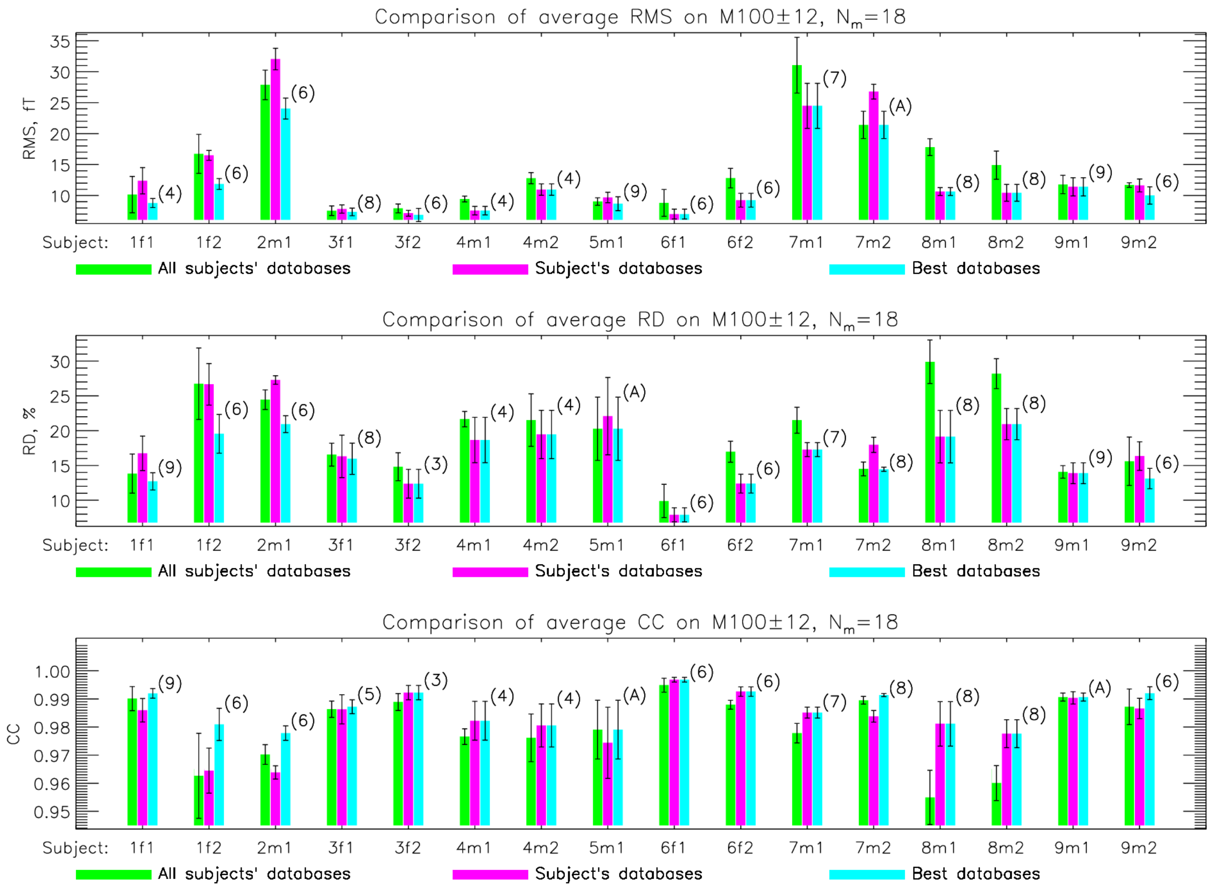

Figure S.9: (Fig.5) Comparison of evaluation results for single measurements on M100±12 ms interval using 18 selected measuring sites from 3600 training maps from SPH simulated databases.

Table S.5: Evaluation results from Fig. S.9 (Fig. 5 in the paper)

Comparison of evaluation results for single measurements on M100±12 ms using 3600 SPH simulated maps, N_{m}=18

	All subjects' databases			Subject's databases			Best databases		
Subject	RMS ± SD	RD ± SD	CC ± SD	RMS ± SD	RD ± SD	CC ± SD	RMS$^{(*)}$ ± SD	RD$^{(*)}$ ± SD	CC$^{(*)}$ ± SD
1f1	10.1 ± 2.93	13.8 ± 2.81	0.99 ± 0.0043	12.4 ± 2.12	16.7 ± 2.48	0.986 ± 0.0042	$8.78^{(4)}$ ± 0.726	$12.7^{(9)}$ ± 1.24	$0.992^{(9)}$ ± 0.0017
1f2	16.7 ± 3.15	26.7 ± 5.13	0.963 ± 0.015	16.5 ± 0.794	26.7 ± 2.99	0.964 ± 0.008	$11.9^{(6)}$ ± 0.883	$19.5^{(6)}$ ± 2.78	$0.981^{(6)}$ ± 0.0057
2m1	27.9 ± 2.38	24.5 ± 1.41	0.97 ± 0.0035	32 ± 1.75	27.3 ± 0.616	0.964 ± 0.0024	$24^{(6)}$ ± 1.7	$20.9^{(6)}$ ± 1.21	$0.978^{(6)}$ ± 0.0026
3f1	7.54 ± 0.776	16.6 ± 1.64	0.986 ± 0.0029	7.81 ± 0.66	16.3 ± 3.05	0.986 ± 0.0052	$7.35^{(8)}$ ± 0.621	$16^{(8)}$ ± 2.24	$0.987^{(5)}$ ± 0.0025
3f2	7.92 ± 0.714	14.8 ± 2.01	0.989 ± 0.003	7.14 ± 0.474	12.4 ± 2.07	0.992 ± 0.0026	$6.86^{(6)}$ ± 1.07	$12.4^{(3)}$ ± 2.07	$0.992^{(3)}$ ± 0.0026
4m1	9.44 ± 0.465	21.7 ± 1.11	0.977 ± 0.0028	7.56 ± 0.676	18.7 ± 3.26	0.982 ± 0.0069	$7.56^{(4)}$ ± 0.676	$18.7^{(4)}$ ± 3.26	$0.982^{(4)}$ ± 0.0069
4m2	12.8 ± 0.91	21.5 ± 3.77	0.976 ± 0.0085	11 ± 0.92	19.4 ± 3.48	0.981 ± 0.0077	$11^{(4)}$ ± 0.92	$19.4^{(4)}$ ± 3.48	$0.981^{(4)}$ ± 0.0077
5m1	9.03 ± 0.601	20.3 ± 4.54	0.979 ± 0.01	9.68 ± 0.842	22.1 ± 5.57	0.974 ± 0.013	$8.68^{(9)}$ ± 1.13	$20.3^{(A)}$ ± 4.54	$0.979^{(A)}$ ± 0.01
6f1	8.81 ± 2.17	9.9 ± 2.4	0.995 ± 0.0025	7 ± 0.803	7.91 ± 1.01	0.997 ± 0.00081	$7^{(6)}$ ± 0.803	$7.91^{(6)}$ ± 1.01	$0.997^{(6)}$ ± 0.00081
6f2	12.8 ± 1.57	17 ± 1.51	0.988 ± 0.0016	9.24 ± 1.11	12.4 ± 1.35	0.993 ± 0.0017	$9.24^{(6)}$ ± 1.11	$12.4^{(6)}$ ± 1.35	$0.993^{(6)}$ ± 0.0017
7m1	31.1 ± 4.5	21.5 ± 1.86	0.978 ± 0.0035	24.5 ± 3.64	17.3 ± 0.997	0.985 ± 0.0019	$24.5^{(7)}$ ± 3.64	$17.3^{(7)}$ ± 0.997	$0.985^{(7)}$ ± 0.0019
7m2	21.4 ± 2.2	14.5 ± 0.987	0.99 ± 0.0014	26.8 ± 1.19	18 ± 1.1	0.984 ± 0.0021	$21.4^{(A)}$ ± 2.2	$14.4^{(8)}$ ± 0.303	$0.991^{(8)}$ ± 0.00055
8m1	17.8 ± 1.36	29.9 ± 3.12	0.955 ± 0.0097	10.6 ± 0.632	19.1 ± 3.77	0.981 ± 0.0079	$10.6^{(8)}$ ± 0.632	$19.1^{(8)}$ ± 3.77	$0.981^{(8)}$ ± 0.0079
8m2	14.9 ± 2.28	28.2 ± 2.15	0.96 ± 0.0062	10.4 ± 1.36	20.9 ± 2.24	0.978 ± 0.005	$10.4^{(8)}$ ± 1.36	$20.9^{(8)}$ ± 2.24	$0.978^{(8)}$ ± 0.005
9m1	11.8 ± 1.47	14.1 ± 0.898	0.991 ± 0.0014	11.4 ± 1.48	13.9 ± 1.5	0.99 ± 0.0022	$11.4^{(9)}$ ± 1.48	$13.9^{(9)}$ ± 1.5	$0.991^{(A)}$ ± 0.0014
9m2	11.7 ± 0.373	15.6 ± 3.47	0.987 ± 0.0063	11.6 ± 1.04	16.3 ± 2.05	0.987 ± 0.0036	$10^{(6)}$ ± 1.39	$13.1^{(6)}$ ± 1.46	$0.992^{(6)}$ ± 0.0023
Σ	14.5 ± 0.093	19.4 ± 0.868	0.98 ± 0.0016	13.5 ± 0.261	17.8 ± 0.512	0.983 ± 0.00091	11.9 ± 0.348	16.2 ± 0.366	0.986 ± 0.00057

$^{(*)}$Best databases: $^{(A)}$ – All subjects' databases (**all-bases**), $^{(n)}$ – n-th subject's databases.

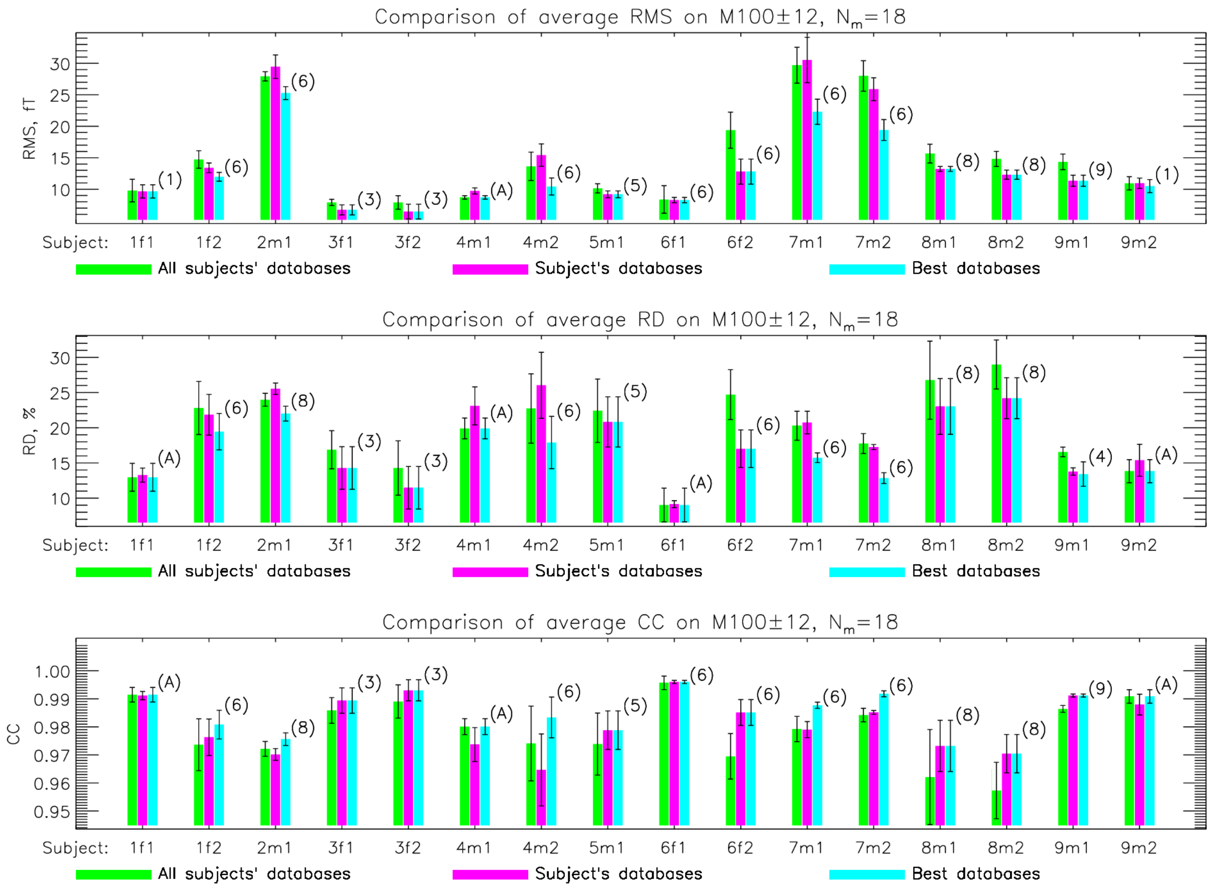

Figure S.10: Comparison of evaluation results for single measurements on M100±12 ms interval using 18 selected measuring sites from 3600 training maps from BEM simulated databases.

Table S.6: Evaluation results from Fig. S.10

Comparison of evaluation results for single measurements on M100±12 ms using 3600 BEM simulated maps, N_{m}=18

	All subjects' databases			Subject's databases			Best databases		
Subject	RMS ± SD	RD ± SD	CC ± SD	RMS ± SD	RD ± SD	CC ± SD	$RMS^{(*)}$ ± SD	$RD^{(*)}$ ± SD	$CC^{(*)}$ ± SD
1f1	9.78 ± 1.8	13 ± 1.99	0.991 ± 0.0026	9.66 ± 1.05	13.3 ± 1	0.991 ± 0.0015	$9.66^{(1)}$ ± 1.05	$13^{(A)}$ ± 1.99	$0.991^{(A)}$ ± 0.0026
1f2	14.7 ± 1.39	22.8 ± 3.77	0.974 ± 0.0092	13.4 ± 0.777	21.8 ± 2.9	0.976 ± 0.0065	$12^{(6)}$ ± 0.703	$19.4^{(6)}$ ± 2.59	$0.981^{(6)}$ ± 0.0051
2m1	27.9 ± 0.736	24 ± 0.917	0.972 ± 0.0026	29.5 ± 1.86	25.5 ± 0.808	0.97 ± 0.0021	$25.3^{(6)}$ ± 1.02	$22^{(8)}$ ± 1.05	$0.976^{(8)}$ ± 0.0022
3f1	7.9 ± 0.493	16.9 ± 2.71	0.986 ± 0.0046	6.73 ± 0.791	14.3 ± 3.03	0.989 ± 0.0045	$6.73^{(3)}$ ± 0.791	$14.3^{(3)}$ ± 3.03	$0.989^{(3)}$ ± 0.0045
3f2	7.9 ± 1.08	14.3 ± 3.86	0.989 ± 0.0059	6.44 ± 1.18	11.5 ± 3.02	0.993 ± 0.0038	$6.44^{(3)}$ ± 1.18	$11.5^{(3)}$ ± 3.02	$0.993^{(3)}$ ± 0.0038
4m1	8.7 ± 0.281	19.9 ± 1.48	0.98 ± 0.0028	9.72 ± 0.496	23.1 ± 2.7	0.974 ± 0.006	$8.7^{(A)}$ ± 0.281	$19.9^{(A)}$ ± 1.48	$0.98^{(A)}$ ± 0.0028
4m2	13.6 ± 2.26	22.7 ± 4.93	0.974 ± 0.013	15.4 ± 1.78	26 ± 4.69	0.965 ± 0.013	$10.4^{(6)}$ ± 1.35	$17.9^{(6)}$ ± 3.72	$0.983^{(6)}$ ± 0.0073
5m1	10.1 ± 0.727	22.4 ± 4.5	0.974 ± 0.011	9.19 ± 0.54	20.8 ± 3.58	0.979 ± 0.0069	$9.19^{(5)}$ ± 0.54	$20.8^{(5)}$ ± 3.58	$0.979^{(5)}$ ± 0.0069
6f1	8.36 ± 2.18	9.02 ± 2.39	0.996 ± 0.0024	8.28 ± 0.415	9.14 ± 0.497	0.996 ± 0.00056	$8.28^{(6)}$ ± 0.415	$9.02^{(A)}$ ± 2.39	$0.996^{(6)}$ ± 0.00056
6f2	19.4 ± 2.87	24.7 ± 3.54	0.97 ± 0.0081	12.8 ± 1.99	17 ± 2.67	0.985 ± 0.0046	$12.8^{(6)}$ ± 1.99	$17^{(6)}$ ± 2.67	$0.985^{(6)}$ ± 0.0046
7m1	29.7 ± 2.85	20.3 ± 2.06	0.979 ± 0.0045	30.5 ± 3.6	20.7 ± 1.61	0.979 ± 0.0029	$22.3^{(6)}$ ± 2	$15.7^{(6)}$ ± 0.691	$0.988^{(6)}$ ± 0.0011
7m2	28 ± 2.42	17.7 ± 1.42	0.984 ± 0.0024	25.9 ± 1.82	17.3 ± 0.354	0.985 ± 0.00067	$19.4^{(6)}$ ± 1.65	$12.8^{(6)}$ ± 0.77	$0.992^{(6)}$ ± 0.0011
8m1	15.7 ± 1.49	26.8 ± 5.55	0.962 ± 0.017	13.2 ± 0.383	23 ± 3.96	0.973 ± 0.0091	$13.2^{(8)}$ ± 0.383	$23^{(8)}$ ± 3.96	$0.973^{(8)}$ ± 0.0091
8m2	14.8 ± 1.2	29 ± 3.48	0.957 ± 0.01	12.3 ± 0.725	24.2 ± 2.91	0.97 ± 0.0068	$12.3^{(8)}$ ± 0.725	$24.2^{(8)}$ ± 2.91	$0.97^{(8)}$ ± 0.0068
9m1	14.3 ± 1.24	16.6 ± 0.681	0.986 ± 0.0012	11.3 ± 0.892	13.8 ± 0.524	0.991 ± 0.00055	$11.3^{(9)}$ ± 0.892	$13.4^{(4)}$ ± 1.73	$0.991^{(9)}$ ± 0.00055
9m2	10.9 ± 1.05	13.8 ± 1.64	0.991 ± 0.0024	10.9 ± 0.819	15.4 ± 2.27	0.988 ± 0.0037	$10.5^{(1)}$ ± 1.04	$13.8^{(A)}$ ± 1.64	$0.991^{(A)}$ ± 0.0024
Σ	15.1 ± 0.262	19.6 ± 0.409	0.979 ± 0.0006	14.1 ± 0.205	18.6 ± 0.568	0.982 ± 0.00092	12.4 ± 0.26	16.7 ± 0.409	0.985 ± 0.0006

$^{(*)}$Best databases: $^{(A)}$ – All subjects' databases (**all-bases**), $^{(n)}$ – n-th subject's databases.

Table S.7: Overview of evaluation results for single measurements using SPH and BEM simulated training databases Comparison of evaluation results for single measurements on M100±12 ms using 3600 SPH simulated maps

	All subjects' databases			Subject's databases			Best databases		
N_m	RMS ± SD	RD ± SD	CC ± SD	RMS ± SD	RD ± SD	CC ± SD	RMS$^{(*)}$ ± SD	RD$^{(*)}$ ± SD	CC$^{(*)}$ ± SD
12	18.7 ± 0.231	25.7 ± 1.09	0.964 ± 0.0025	18.6 ± 0.306	24.7 ± 0.881	0.969 ± 0.0022	15.9 ± 0.231	21.7 ± 1.09	0.976 ± 0.0013
18	14.5 ± 0.093	19.4 ± 0.868	0.98 ± 0.0016	13.5 ± 0.261	17.8 ± 0.512	0.983 ± 0.00091	11.9 ± 0.348	16.2 ± 0.366	0.986 ± 0.00057
24	11.7 ± 0.102	15.2 ± 0.614	0.988 ± 0.0009	11.1 ± 0.392	14.8 ± 0.116	0.987 ± 0.00012	9.68 ± 0.392	13 ± 0.169	0.991 ± 0.0002
30	9.14 ± 0.238	11.5 ± 0.153	0.992 ± 0.00011	8.94 ± 0.178	11.7 ± 0.145	0.992 ± 0.0001	8.09 ± 0.178	10.5 ± 0.145	0.994 ± 0.0001

Comparison of evaluation results for single measurements on M100±12 ms using 3600 BEM simulated maps

	All subjects' databases			Subject's databases			Best databases		
N_m	RMS ± SD	RD ± SD	CC ± SD	RMS ± SD	RD ± SD	CC ± SD	RMS$^{(*)}$ ± SD	RD$^{(*)}$ ± SD	CC$^{(*)}$ ± SD
12	19.3 ± 0.306	25.4 ± 0.91	0.966 ± 0.0024	19.6 ± 0.288	25.3 ± 0.987	0.967 ± 0.0024	16.4 ± 0.497	21.9 ± 0.252	0.974 ± 0.00052
18	15.1 ± 0.262	19.6 ± 0.409	0.979 ± 0.0006	14.1 ± 0.205	18.6 ± 0.568	0.982 ± 0.00092	12.4 ± 0.26	16.7 ± 0.409	0.985 ± 0.0006
24	11.3 ± 0.345	15 ± 0.283	0.987 ± 0.00033	11.5 ± 0.322	15.4 ± 0.238	0.987 ± 0.00033	10 ± 0.345	13.4 ± 0.283	0.99 ± 0.00033
30	9.98 ± 0.435	12.7 ± 0.281	0.991 ± 0.00028	9.5 ± 0.437	12.3 ± 0.191	0.991 ± 0.00026	8.29 ± 0.262	10.9 ± 0.262	0.993 ± 0.00025

Comparison of evaluation results for single measurements on M50±6 ms using 3600 SPH simulated maps

	All subjects' databases			Subject's databases			Best databases		
N_m	RMS ± SD	RD ± SD	CC ± SD	RMS ± SD	RD ± SD	CC ± SD	RMS$^{(*)}$ ± SD	RD$^{(*)}$ ± SD	CC$^{(*)}$ ± SD
12	13.8 ± 0.456	45.8 ± 1.4	0.874 ± 0.0079	12.8 ± 0.165	43.2 ± 2.5	0.891 ± 0.013	11.1 ± 0.144	37.5 ± 2.3	0.915 ± 0.0095
18	9.83 ± 0.25	34 ± 1.47	0.927 ± 0.0056	10.3 ± 0.178	34.6 ± 1.76	0.929 ± 0.0068	8.65 ± 0.178	30.5 ± 1.76	0.944 ± 0.0063
24	7.59 ± 0.258	27.1 ± 1.28	0.955 ± 0.0045	8.18 ± 0.091	29.3 ± 1.67	0.944 ± 0.0051	6.69 ± 0.247	23.9 ± 1.22	0.962 ± 0.0043
30	6.26 ± 0.149	21.8 ± 1.3	0.968 ± 0.0038	6.32 ± 0.161	22.7 ± 0.603	0.965 ± 0.0013	5.5 ± 0.161	19.7 ± 0.603	0.972 ± 0.0013

Comparison of evaluation results for single measurements on M50±6 ms using 3600 BEM simulated maps

	All subjects' databases			Subject's databases			Best databases		
N_m	RMS ± SD	RD ± SD	CC ± SD	RMS ± SD	RD ± SD	CC ± SD	RMS$^{(*)}$ ± SD	RD$^{(*)}$ ± SD	CC$^{(*)}$ ± SD
12	13.2 ± 0.109	44.6 ± 2.24	0.886 ± 0.0086	13.3 ± 0.14	43.3 ± 2.69	0.89 ± 0.0098	11.2 ± 0.19	37.2 ± 1.97	0.916 ± 0.009
18	10.4 ± 0.047	35.3 ± 2.6	0.925 ± 0.01	9.59 ± 0.09	32.6 ± 2.51	0.938 ± 0.0071	8.57 ± 0.09	28.9 ± 2.51	0.948 ± 0.0071
24	8.25 ± 0.151	29.3 ± 1.14	0.945 ± 0.0032	8.35 ± 0.069	29.8 ± 1.85	0.943 ± 0.0054	7.18 ± 0.151	25.1 ± 1.14	0.957 ± 0.0032
30	6.98 ± 0.27	25.2 ± 0.421	0.957 ± 0.0011	6.23 ± 0.019	22.9 ± 1.23	0.964 ± 0.0023	5.59 ± 0.019	20 ± 1.23	0.973 ± 0.0023

Comparison of evaluation results for single measurements on [42,240] ms using 3600 SPH simulated maps

	All subjects' databases			Subject's databases			Best databases		
N_m	RMS ± SD	RD ± SD	CC ± SD	RMS ± SD	RD ± SD	CC ± SD	RMS$^{(*)}$ ± SD	RD$^{(*)}$ ± SD	CC$^{(*)}$ ± SD
12	17.2 ± 0.555	34.7 ± 2.15	0.93 ± 0.0081	16.5 ± 0.714	32.7 ± 2.14	0.939 ± 0.0082	15 ± 0.714	30.6 ± 1.73	0.945 ± 0.0068
18	12.6 ± 0.454	25.1 ± 1.83	0.963 ± 0.0053	12.5 ± 0.435	24.8 ± 1.88	0.964 ± 0.0054	11.1 ± 0.435	22.9 ± 1.88	0.969 ± 0.005
24	9.09 ± 0.347	18.4 ± 1.7	0.98 ± 0.0044	9.66 ± 0.281	19 ± 1.62	0.979 ± 0.0037	8.38 ± 0.281	17.2 ± 1.62	0.982 ± 0.0037
30	7.17 ± 0.329	14.2 ± 1.45	0.988 ± 0.0029	7.2 ± 0.242	14.3 ± 1.18	0.988 ± 0.0018	6.69 ± 0.242	13.4 ± 1.18	0.989 ± 0.0018

Comparison of evaluation results for single measurements on [42,240] ms using 3600 BEM simulated maps

	All subjects' databases			Subject's databases			Best databases		
N_m	RMS ± SD	RD ± SD	CC ± SD	RMS ± SD	RD ± SD	CC ± SD	RMS$^{(*)}$ ± SD	RD$^{(*)}$ ± SD	CC$^{(*)}$ ± SD
12	17.4 ± 0.978	33.7 ± 2.08	0.935 ± 0.0067	17.7 ± 0.806	34.8 ± 2.29	0.931 ± 0.0084	15.5 ± 0.644	31.1 ± 2.44	0.943 ± 0.0093
18	13.1 ± 0.472	25.4 ± 2.3	0.963 ± 0.0068	13.2 ± 0.701	26 ± 2.16	0.962 ± 0.0059	11.2 ± 0.392	22.9 ± 2.3	0.97 ± 0.0059
24	10 ± 0.435	20.2 ± 1.58	0.976 ± 0.0034	10.1 ± 0.322	19.7 ± 1.5	0.977 ± 0.0036	9.47 ± 0.435	19 ± 1.58	0.979 ± 0.0034
30	8.17 ± 0.422	16.2 ± 1.22	0.984 ± 0.0022	7.48 ± 0.417	14.4 ± 1.09	0.987 ± 0.0019	6.95 ± 0.408	13.7 ± 1.06	0.989 ± 0.0018

Comparison of evaluation results for single measurements on [0,400] ms using 3600 SPH simulated maps

	All subjects' databases			Subject's databases			Best databases		
N_m	RMS ± SD	RD ± SD	CC ± SD	RMS ± SD	RD ± SD	CC ± SD	RMS$^{(*)}$ ± SD	RD$^{(*)}$ ± SD	CC$^{(*)}$ ± SD
12	14.9 ± 0.636	38 ± 3.18	0.915 ± 0.014	14.7 ± 0.674	36.8 ± 2.95	0.921 ± 0.014	13.4 ± 0.674	34.5 ± 2.95	0.929 ± 0.013
18	11.4 ± 0.503	29.1 ± 2.82	0.95 ± 0.011	11.3 ± 0.449	28.7 ± 2.66	0.951 ± 0.01	10.2 ± 0.449	26.9 ± 2.66	0.956 ± 0.01
24	8.51 ± 0.401	21.9 ± 2.53	0.97 ± 0.0082	8.86 ± 0.32	22.4 ± 2.06	0.97 ± 0.0055	7.88 ± 0.32	20.5 ± 2.06	0.974 ± 0.0055
30	6.8 ± 0.284	17.2 ± 2	0.982 ± 0.0048	6.67 ± 0.29	17.1 ± 1.7	0.982 ± 0.0036	6.29 ± 0.29	16.3 ± 1.7	0.983 ± 0.0036

Comparison of evaluation results for single measurements on [0,400] ms using 3600 BEM simulated maps

	All subjects' databases			Subject's databases			Best databases		
N_m	RMS ± SD	RD ± SD	CC ± SD	RMS ± SD	RD ± SD	CC ± SD	RMS$^{(*)}$ ± SD	RD$^{(*)}$ ± SD	CC$^{(*)}$ ± SD
12	15.7 ± 0.869	38.2 ± 3.14	0.916 ± 0.014	15.7 ± 0.904	38.6 ± 3.51	0.914 ± 0.017	14 ± 0.648	35 ± 2.83	0.927 ± 0.012
18	11.9 ± 0.459	29 ± 2.75	0.95 ± 0.0097	12 ± 0.708	29.8 ± 3.14	0.948 ± 0.013	10.2 ± 0.467	26.5 ± 2.25	0.958 ± 0.0076
24	9.16 ± 0.392	23.3 ± 2	0.967 ± 0.0051	9.23 ± 0.328	23.1 ± 2.25	0.968 ± 0.0071	8.68 ± 0.392	22.1 ± 2	0.971 ± 0.0051
30	7.55 ± 0.368	18.9 ± 1.83	0.978 ± 0.0045	6.85 ± 0.399	16.9 ± 1.46	0.982 ± 0.0033	6.49 ± 0.399	16.4 ± 1.46	0.983 ± 0.0033

S.4 Localizations of M100 for all measurements

Comparisons of M100 source localization for all cases. First we applied SSA using SPH simulated data from all subjects and all simulation protocols (**all-bases-sph**) to find the first 18 optimal measuring sites. Then we localized M100 with a single (fit1d) or two dipole sources (fit2d) from the measured MFM, the estimated MFM and from the selected sites only. All results are displayed in the following figures. In figure captions, all coordinates and localization errors are expressed in units of mm, dipole moments in μAm, and dipole orientation errors with angular degrees. We applied one dipole source for cases where AEF signal is observed only on one hemisphere.

On each figure, 10 maps are presented in five columns and two rows, radial component maps in the first and tangential component maps in the second. All maps are displayed with red, blue and green isolines representing positive, negative and zero field values. At the bottom of each map the RMS(value) is denoted, and minimum and maximum values are labeled by "m:" and "M:" on the left and right bottom sides, respectively. All magnetic field values are displayed in units of fT. Measured maps are presented in the first column (a). Field component is denoted at the top followed by subject's name in parenthesis. On the left top a time trace of all RMS(map) values (from 0 to 400 ms) are displayed with vertical line marking the time of the presented map, denoted by "t:" on the right top side. Measuring sites are denoted by red plus signs and blue minus signs for the corresponding positive and negative field values, respectively. Estimated maps are presented in the second column (b). At the top SSA evaluation results (RMS, CC, and RD) are denoted. Selected sites are labeled with circles, and the number selected sites are denoted at the top right side. Localization results obtained from the measured map in column(a), estimated map in column(b), and from selected sites only are displayed in the third (c), fourth (d), and fifth (e) columns, respectively. Each of dipole's 3D position and direction are projected into a 2D map and marked by a black arrow with the size corresponding to the dipole moment value. Evaluation of fit results, i.e. comparisons of RMS, CC, and RD between measured field and field calculated from the obtained dipoles are denoted at the top of maps.

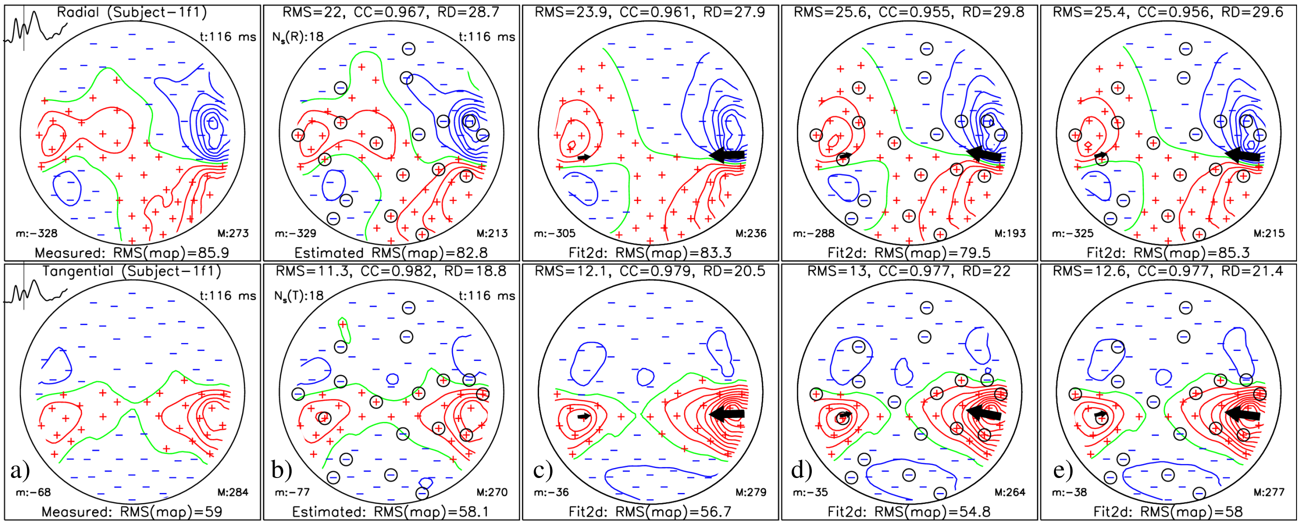

Figure S.11: Subject-1f1: 18 selected sites using **all-bases-sph**, fitting M100 with 2 dipoles:
a) Measured data, b) Estimated data map,
c) measured map fit: $\vec{r}_1$=(58.8,-15.3,18.7), $\vec{r}_2$=(-54.6,-19,32.1), $\vec{p}_1$=(3.1,0.7,-9.1), $\vec{p}_2$=(-1.6,-0.1,-2.9),
d) estimated map fit: $\vec{r}_1$=(57.1,-14.9,20.9), $\vec{r}_2$=(-53.3,-17.9,35.3), $\vec{p}_1$=(3.2,-0.5,-9.2), $\vec{p}_2$=(-1.7,-0.4,-2.8),
reconstructed source errors: Δr_1=2.8, Δr_2=3.6, Δr_c=4.6, $\Delta\varphi_1$=6.98, $\Delta\varphi_2$=5.31,
e) selected chan. fit: $\vec{r}_1$=(58.2,-16,21.3), $\vec{r}_2$=(-54.6,-16.8,31), $\vec{p}_1$=(3.3,-0.1,-9.2), $\vec{p}_2$=(-1.6,-0.3,-2.9),
reconstructed source errors: Δr_1=2.7, Δr_2=2.5, Δr_c=3.7, $\Delta\varphi_1$=4.96, $\Delta\varphi_2$=3.43.

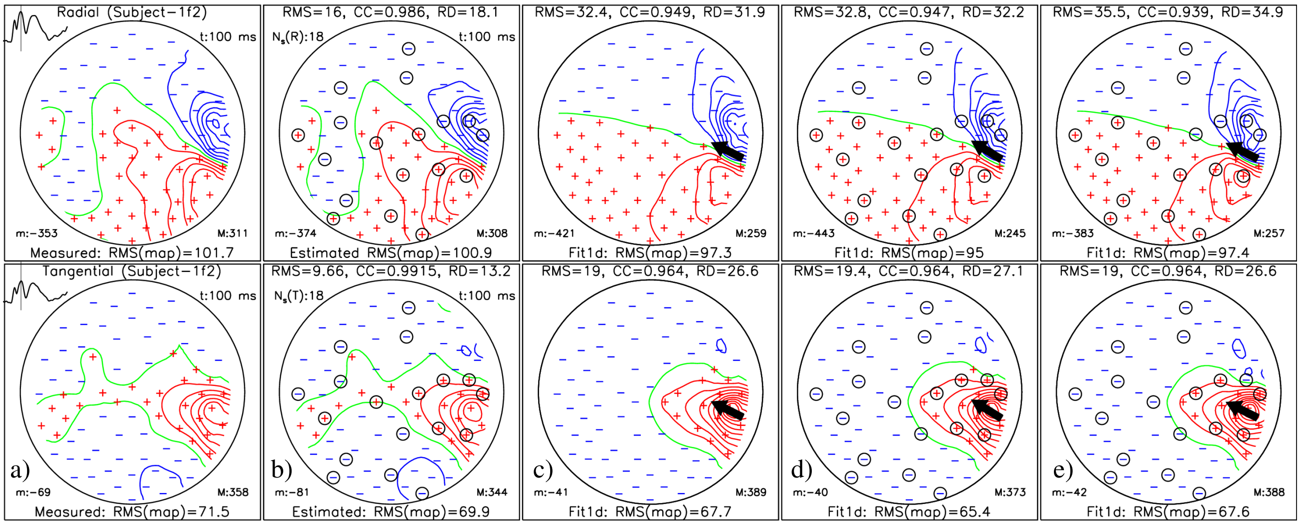

Figure S.12: Subject-1f2: 18 selected sites using **all-bases-sph**, fitting M100 with 1 dipole:
a) Measured data, b) Estimated data map,
c) measured map fit: $\vec{r}$=(58.4,-11.8,19.2), $\vec{p}$=(3.2,-2.4,-11.4),
d) estimated map fit – source parameters: $\vec{r}$=(57.8,-11.3,18.6), $\vec{p}$=(3.1,-3.1,-11.5),
reconstructed source errors: $\Delta\vec{r}$=(-0.7,0.4,-0.6), Δr=1, $\Delta\vec{p}$=(-0.2,-0.6,-0.1), Δp=0.7, $\Delta\varphi$=0.053,
e) selected chan. fit: $\vec{r}$=(59.8,-13,23.5), $\vec{p}$=(3.4,-2.3,-9.8),
reconstructed source errors: $\Delta\vec{r}$=(1.4,-1.2,4.3), Δr=4.6, $\Delta\vec{p}$=(0.1,0.1,1.5), Δp=1.5, $\Delta\varphi$=0.052.

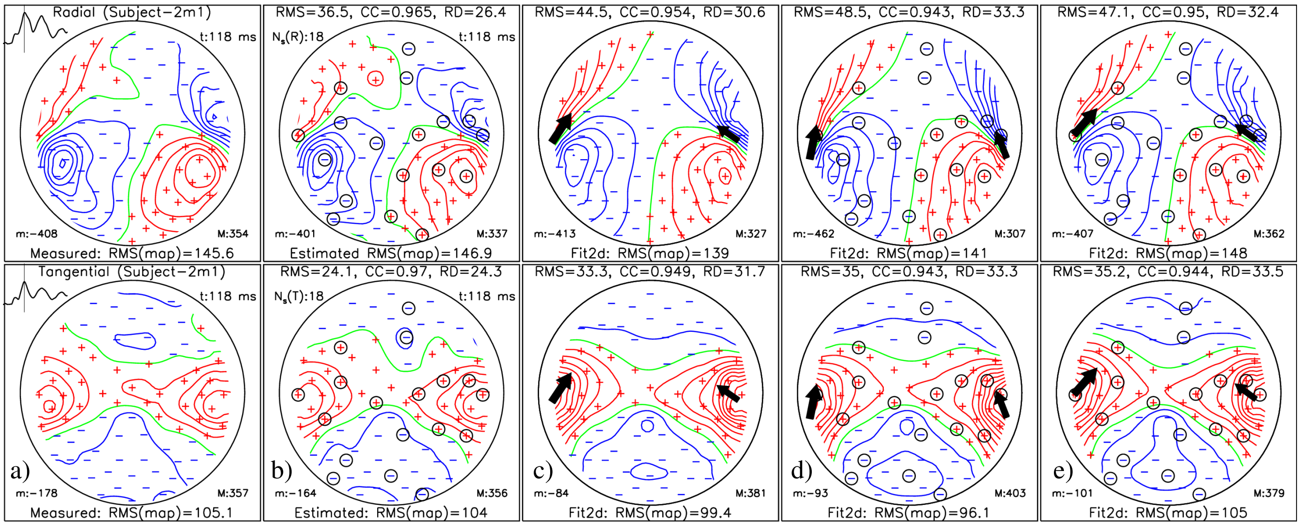

Figure S.13: Subject-2m1: 18 selected sites using **all-bases-sph**, fitting M100 with 2 dipoles:
a) Measured data, b) Estimated data map,
c) measured map fit: $\vec{r}_1$=(44.5,0.9,15.1), $\vec{r}_2$=(-37.8,3,6.2), $\vec{p}_1$=(10.5,-12.1,-30.1), $\vec{p}_2$=(-8.1,-26.3,-36.7),
d) estimated map fit: $\vec{r}_1$=(44.4,-4.7,1.4), $\vec{r}_2$=(-37.8,-2.8,0.6), $\vec{p}_1$=(-1.3,-23.1,-36.7), $\vec{p}_2$=(2.2,-37,-39),
reconstructed source errors: Δr_1=14.8, Δr_2=8.1, Δr_c=16.9, $\Delta\varphi_1$=22.1, $\Delta\varphi_2$=14.8,
e) selected chan. fit: $\vec{r}_1$=(39.3,1.5,13.6), $\vec{r}_2$=(-32.5,5.3,8.7), $\vec{p}_1$=(15.4,-18.8,-42.6), $\vec{p}_2$=(-19.9,-37,-51.8),
reconstructed source errors: Δr_1=5.5, Δr_2=6.2, Δr_c=8.3, $\Delta\varphi_1$=1.84, $\Delta\varphi_2$=7.15.

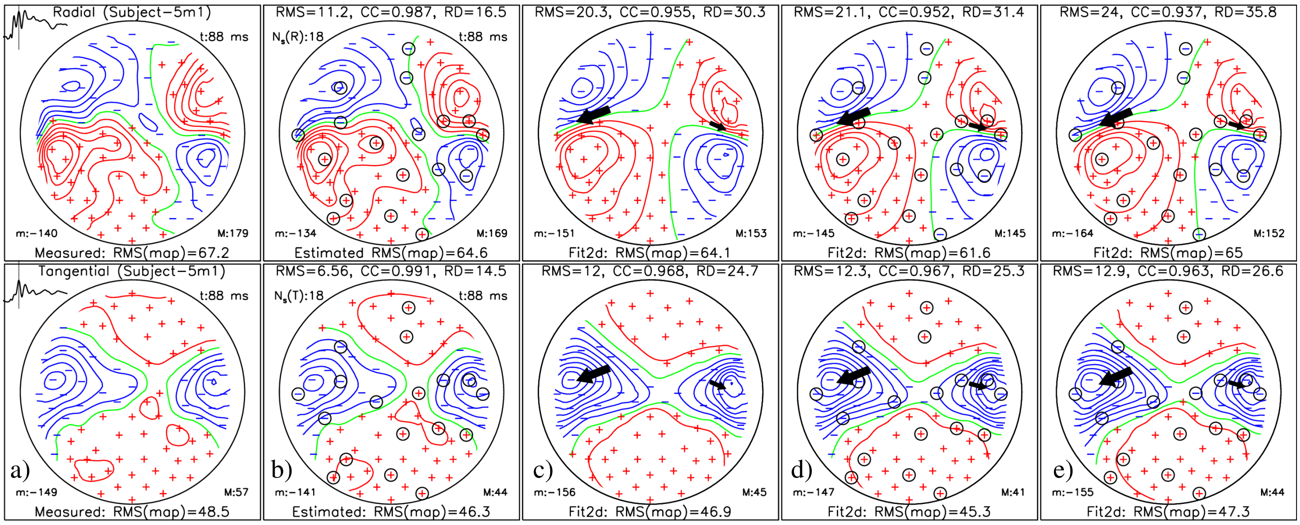

Figure S.14: Subject-5m1: 18 selected sites using **all-bases-sph**, fitting M100 with 2 dipoles:
a) Measured data, b) Estimated data map,
c) measured map fit: $\vec{r}_1$=(50.6,6.3,27.3), $\vec{r}_2$=(-35,12.2,29.1), $\vec{p}_1$=(-3.6,1.4,6.4), $\vec{p}_2$=(10.5,3.2,11.3),
d) estimated map fit: $\vec{r}_1$=(51.1,5.8,26.8), $\vec{r}_2$=(-35.1,11.3,30.6), $\vec{p}_1$=(-3.3,0.7,6.2), $\vec{p}_2$=(9.9,2.9,10.3),
reconstructed source errors: Δr_1=0.9, Δr_2=1.7, Δr_c=1.9, $\Delta\varphi_1$=5.32, $\Delta\varphi_2$=0.961,
e) selected chan. fit: $\vec{r}_1$=(51.3,6.8,26.6), $\vec{r}_2$=(-34.9,11.2,33.8), $\vec{p}_1$=(-3.2,0.8,6), $\vec{p}_2$=(10.6,3.9,9.7),
reconstructed source errors: Δr_1=1.1, Δr_2=4.8, Δr_c=4.9, $\Delta\varphi_1$=4.25, $\Delta\varphi_2$=5.73.

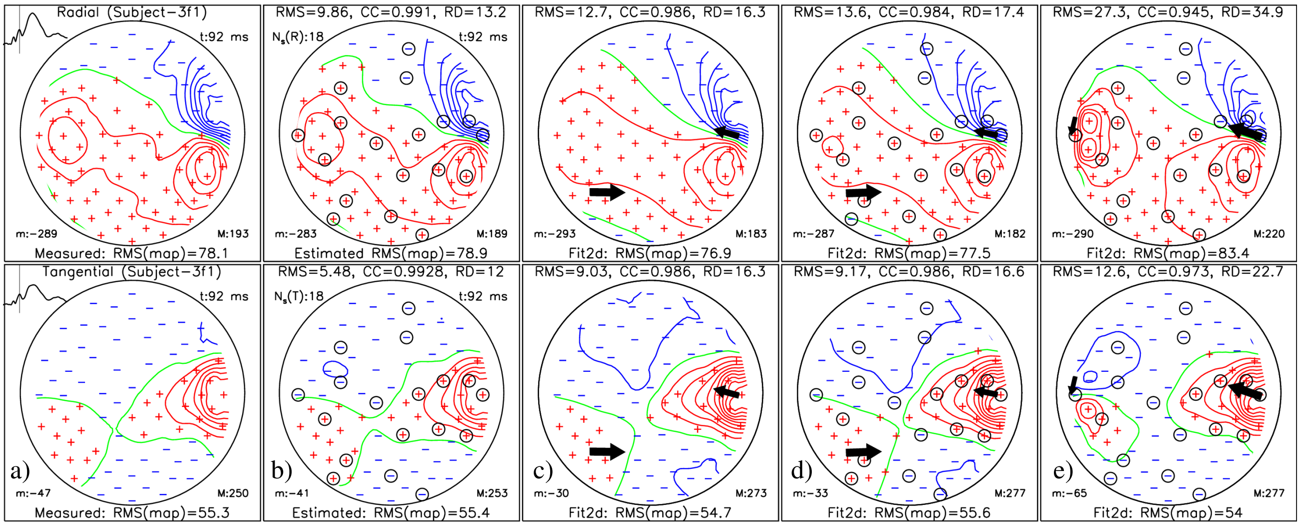

Figure S.15: Subject-3f1: 18 selected sites using **all-bases-sph**, fitting M100 with 2 dipoles:
a) Measured data, b) Estimated data map,
c) measured map fit: $\vec{r}_1$=(51.6,1.6,19), $\vec{r}_2$=(-13.5,-18.6,11.7), $\vec{p}_1$=(5,-2.3,-13.5), $\vec{p}_2$=(-14.3,1.9,-13.5),
d) estimated map fit: $\vec{r}_1$=(50.5,1.4,18.3), $\vec{r}_2$=(-13.2,-17.2,10.2), $\vec{p}_1$=(5.3,-1.8,-14.6), $\vec{p}_2$=(-15.4,1.7,-17.1),
reconstructed source errors: Δr_1=1.3, Δr_2=2.1, Δr_c=2.5, $\Delta\varphi_1$=2.51, $\Delta\varphi_2$=4.82,
e) selected chan. fit: $\vec{r}_1$=(56.4,2.5,20.2), $\vec{r}_2$=(-64.5,6,1.9), $\vec{p}_1$=(3.4,-1.9,-9.2), $\vec{p}_2$=(0.5,5.2,-0.1),
reconstructed source errors: Δr_1=5, Δr_2=57.5, Δr_c=57.7, $\Delta\varphi_1$=2.24, $\Delta\varphi_2$=87.5.

M10

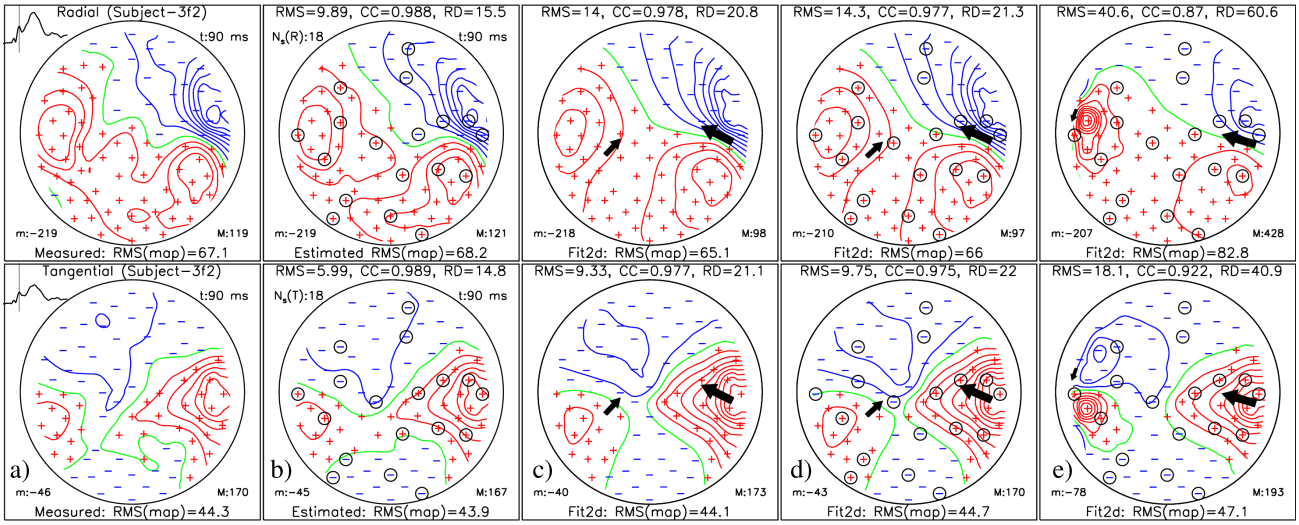

Figure S.16: Subject-3f2: 18 selected sites using **all-bases-sph**, fitting M100 with 2 dipoles:
a) Measured data, b) Estimated data map,
c) measured map fit: $\vec{r}_1$=(38.5,0.4,21.5), $\vec{r}_2$=(-18.1,-6.2,30.1), $\vec{p}_1$=(9.8,-6.5,-17.4), $\vec{p}_2$=(-7.6,-8.4,-6.3),
d) estimated map fit: $\vec{r}_1$=(36.6,0.5,20.5), $\vec{r}_2$=(-16.5,-7.4,30.6), $\vec{p}_1$=(11.2,-6.6,-19.9), $\vec{p}_2$=(-9.2,-8.6,-7),
reconstructed source errors: Δr_1=2.2, Δr_2=2, Δr_c=3, $\Delta\varphi_1$=2.07, $\Delta\varphi_2$=4.13,
e) selected chan. fit: $\vec{r}_1$=(47.4,-3.1,21.8), $\vec{r}_2$=(-73.3,15.6,2.6), $\vec{p}_1$=(4.8,-1.9,-10.7), $\vec{p}_2$=(0.9,4.1,0),
reconstructed source errors: Δr_1=9.5, Δr_2=65.4, Δr_c=66.1, $\Delta\varphi_1$=10.3, $\Delta\varphi_2$=139.

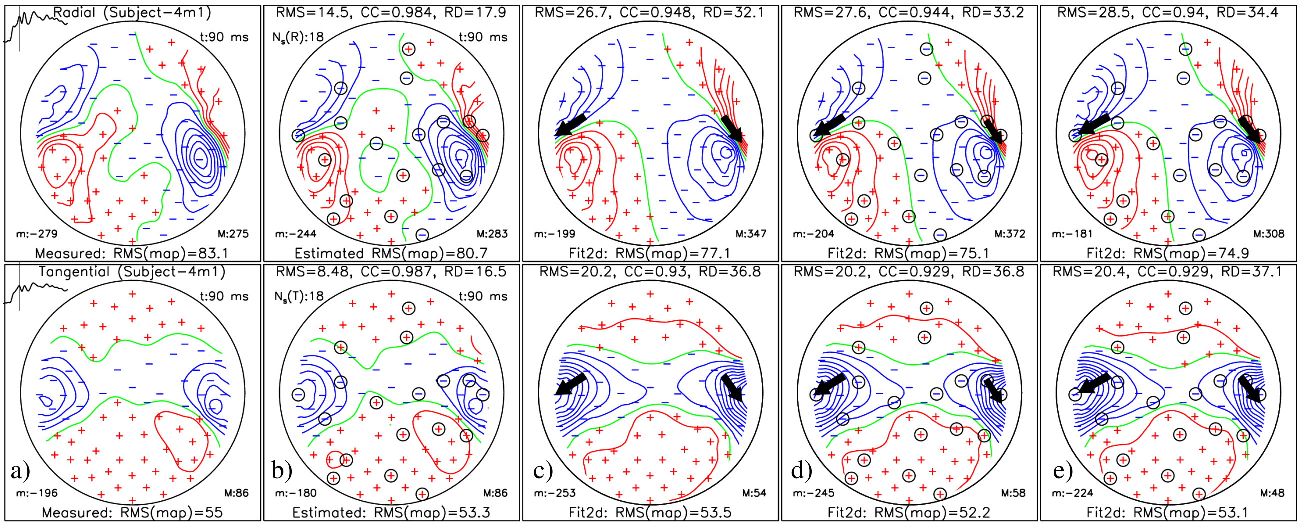

Figure S.17: Subject-4m1: 18 selected sites using **all-bases-sph**, fitting M100 with 2 dipoles:
a) Measured data, b) Estimated data map,
c) measured map fit: $\vec{r}_1$=(54.7,3.5,13), $\vec{r}_2$=(-46.2,5.4,15.3), $\vec{p}_1$=(-2.5,8,8.3), $\vec{p}_2$=(4.3,4.6,11.3),
d) estimated map fit: $\vec{r}_1$=(57,1.8,11.1), $\vec{r}_2$=(-46.6,5.4,15.5), $\vec{p}_1$=(-1.6,6.9,7.2), $\vec{p}_2$=(4.1,4.2,11),
reconstructed source errors: Δr_1=3.5, Δr_2=0.5, Δr_c=3.5, $\Delta\varphi_1$=2.98, $\Delta\varphi_2$=0.867,
e) selected chan. fit: $\vec{r}_1$=(54.3,2.8,13.3), $\vec{r}_2$=(-47.3,6.6,21.2), $\vec{p}_1$=(-2.3,6.8,7.9), $\vec{p}_2$=(4.9,3.5,9.8),
reconstructed source errors: Δr_1=0.9, Δr_2=6, Δr_c=6.1, $\Delta\varphi_1$=2.98, $\Delta\varphi_2$=6.24.

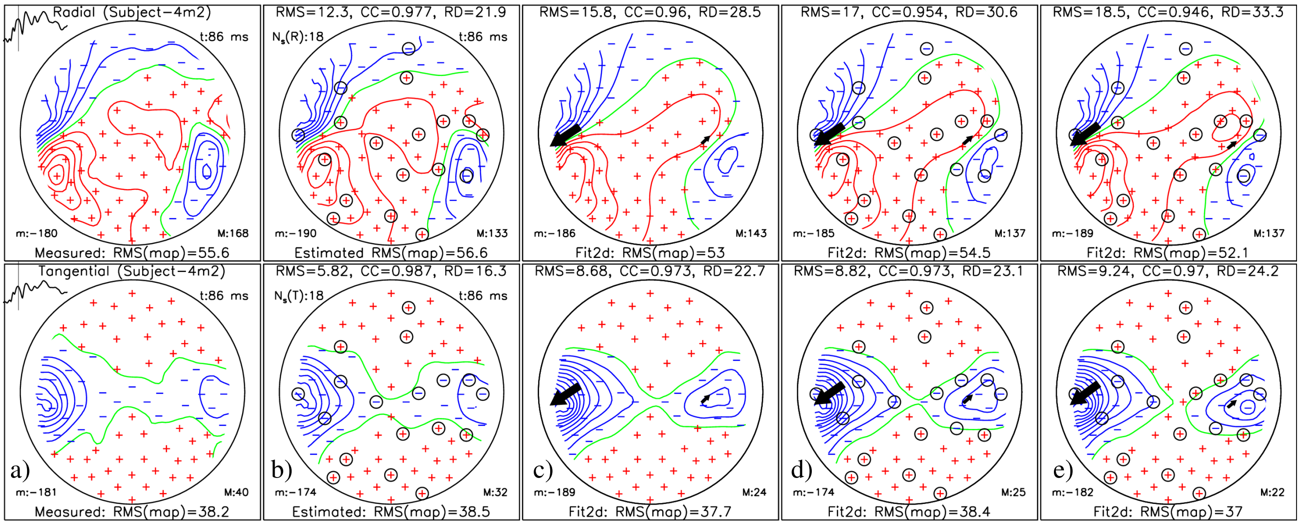

Figure S.18: Subject-4m2: 18 selected sites using **all-bases-sph**, fitting M100 with 2 dipoles:
a) Measured data, b) Estimated data map,
c) measured map fit: $\vec{r}_1$=(47.1,-4.3,39.9), $\vec{r}_2$=(-46.6,-0.9,11), $\vec{p}_1$=(-1.9,-1.9,2.1), $\vec{p}_2$=(2.5,3.4,10.9),
d) estimated map fit: $\vec{r}_1$=(46.9,-4.1,35.7), $\vec{r}_2$=(-45.2,-0.3,15.2), $\vec{p}_1$=(-2.2,-2.1,2.6), $\vec{p}_2$=(3.7,3.9,11),
reconstructed source errors: Δr_1=4.2, Δr_2=4.5, Δr_c=6.2, $\Delta\varphi_1$=3.91, $\Delta\varphi_2$=5.53,
e) selected chan. fit: $\vec{r}_1$=(53,-8.3,33.2), $\vec{r}_2$=(-47.8,-0.3,12.8), $\vec{p}_1$=(-1.6,-1.6,2.2), $\vec{p}_2$=(2.5,3.6,9.6),
reconstructed source errors: Δr_1=9.8, Δr_2=2.2, Δr_c=10, $\Delta\varphi_1$=6.61, $\Delta\varphi_2$=3.32.

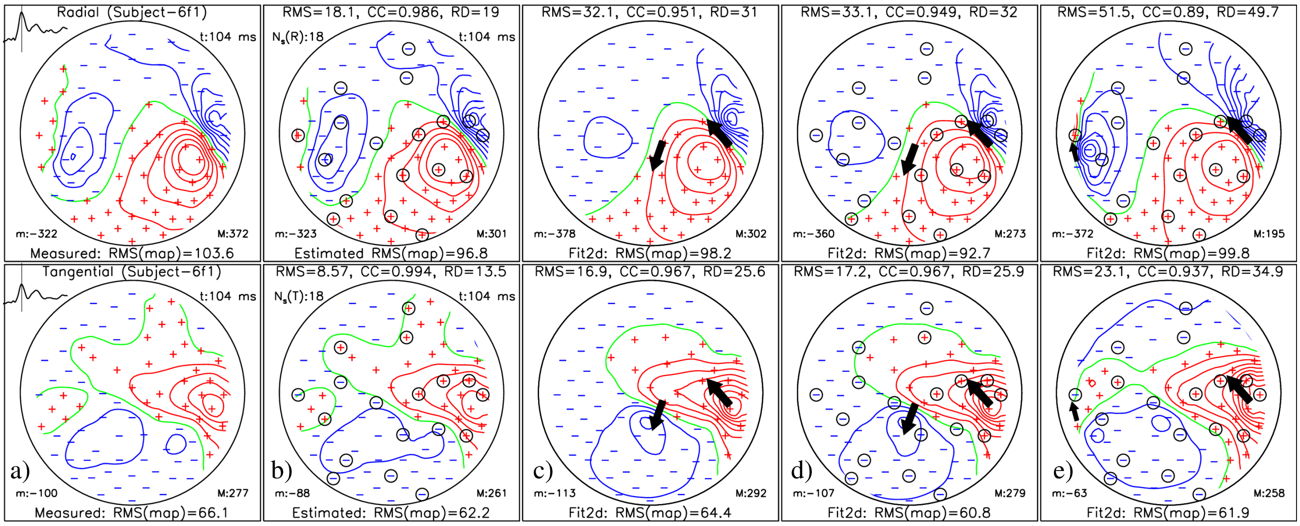

Figure S.19: Subject-6f1: 18 selected sites using **all-bases-sph**, fitting M100 with 2 dipoles:
a) Measured data, b) Estimated data map,
c) measured map fit: $\vec{r}_1$=(55.3,1.8,29.6), $\vec{r}_2$=(6.6,-16,49.4), $\vec{p}_1$=(4.4,-6.5,-7.8), $\vec{p}_2$=(3.1,9.1,2.5),
d) estimated map fit: $\vec{r}_1$=(56.1,1.7,28.5), $\vec{r}_2$=(1.3,-18.5,48.1), $\vec{p}_1$=(3.9,-5.6,-7.3), $\vec{p}_2$=(3,8.3,3.1),
reconstructed source errors: Δr_1=1.4, Δr_2=6.1, Δr_c=6.2, $\Delta\varphi_1$=2.03, $\Delta\varphi_2$=4.88,
e) selected chan. fit: $\vec{r}_1$=(45.2,3.6,22), $\vec{r}_2$=(-53.5,-9.7,1.9), $\vec{p}_1$=(8.8,-13,-16), $\vec{p}_2$=(2.1,-11.8,-1.9),
reconstructed source errors: Δr_1=12.7, Δr_2=76.9, Δr_c=77.9, $\Delta\varphi_1$=0.528, $\Delta\varphi_2$=151.

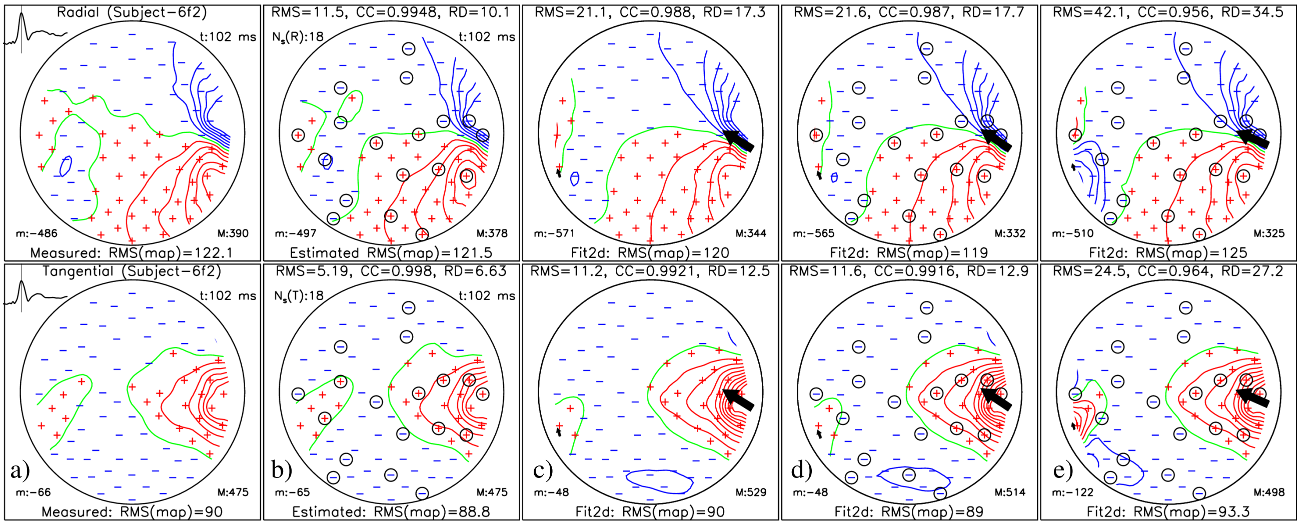

Figure S.20: Subject-6f2: 18 selected sites using **all-bases-sph**, fitting M100 with 2 dipoles:
a) Measured data, b) Estimated data map,
c) measured map fit: $\vec{r}_1$=(52.8,-3.1,7.4), $\vec{r}_2$=(-52,-22.2,-2.7), $\vec{p}_1$=(3,-5.8,-23.6), $\vec{p}_2$=(1.9,-4.1,-3.1),
d) estimated map fit: $\vec{r}_1$=(52.6,-2.7,8.6), $\vec{r}_2$=(-50.8,-23.2,-2.1), $\vec{p}_1$=(3.5,-6.5,-23.2), $\vec{p}_2$=(2.1,-4.4,-2.8),
reconstructed source errors: Δr_1=1.3, Δr_2=1.7, Δr_c=2.1, $\Delta\varphi_1$=2.11, $\Delta\varphi_2$=4.44,
e) selected chan. fit: $\vec{r}_1$=(52.6,-2.2,11.2), $\vec{r}_2$=(-69.2,-24,-5.1), $\vec{p}_1$=(4.5,-4.4,-22.1), $\vec{p}_2$=(1.4,-3.5,-1.9),
reconstructed source errors: Δr_1=3.9, Δr_2=17.5, Δr_c=17.9, $\Delta\varphi_1$=5, $\Delta\varphi_2$=8.25.

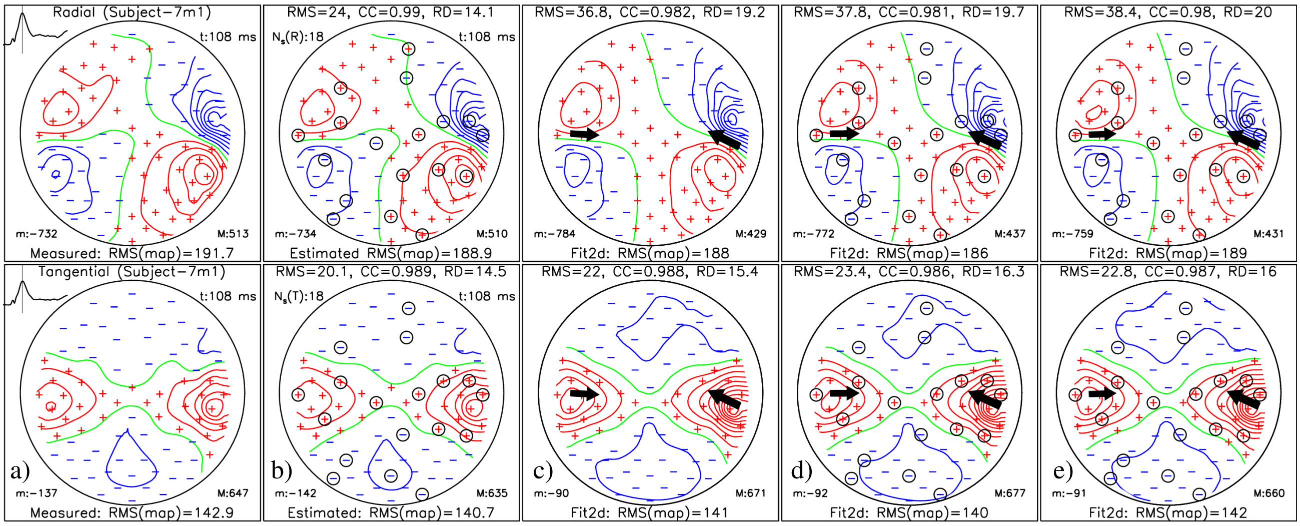

Figure S.21: Subject-7m1: 18 selected sites using **all-bases-sph**, fitting M100 with 2 dipoles:
a) Measured data, b) Estimated data map,
c) measured map fit: $\vec{r}_1$=(54.7,-2.8,22.8), $\vec{r}_2$=(-43.6,0.4,28.7), $\vec{p}_1$=(9.6,-6.9,-23.9), $\vec{p}_2$=(-11.9,0.8,-18),
d) estimated map fit: $\vec{r}_1$=(54.9,-3.1,21.1), $\vec{r}_2$=(-42.4,0.9,28.7), $\vec{p}_1$=(9.1,-6.4,-24.6), $\vec{p}_2$=(-12.4,-0.2,-18.4),
reconstructed source errors: Δr_1=1.7, Δr_2=1.3, Δr_c=2.2, $\Delta\varphi_1$=2.08, $\Delta\varphi_2$=2.66,
e) selected chan. fit: $\vec{r}_1$=(53.4,-3,21.1), $\vec{r}_2$=(-43.4,0.5,28.2), $\vec{p}_1$=(10.1,-7.1,-26.6), $\vec{p}_2$=(-12.2,-0.6,-18.8),
reconstructed source errors: Δr_1=2.2, Δr_2=0.6, Δr_c=2.3, $\Delta\varphi_1$=1.58, $\Delta\varphi_2$=3.66.

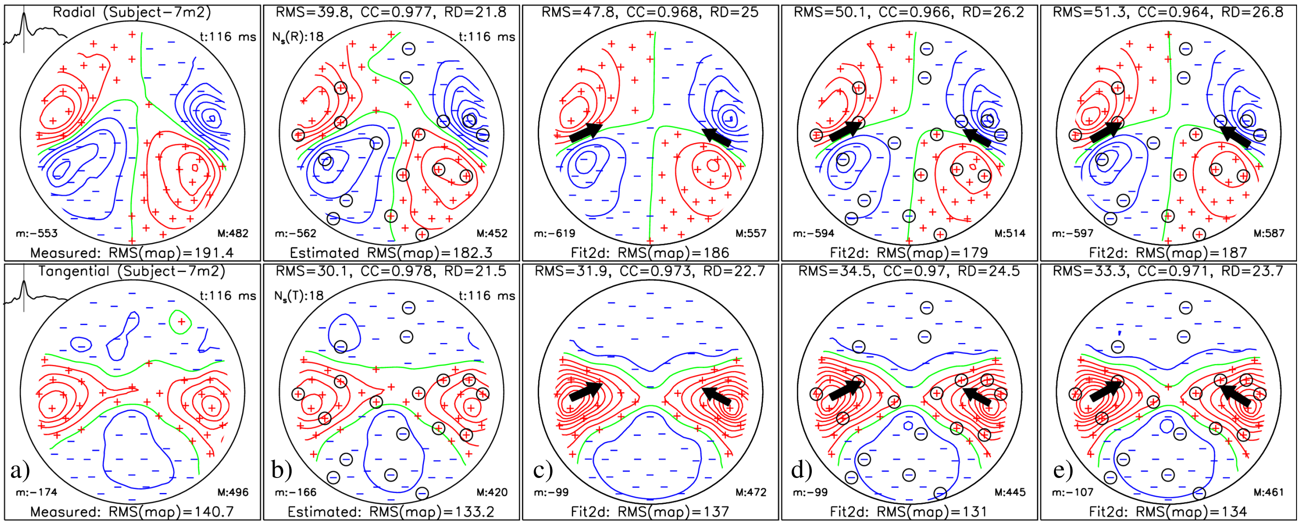

Figure S.22: Subject-7m2: 18 selected sites using **all-bases-sph**, fitting M100 with 2 dipoles:
a) Measured data, b) Estimated data map,
c) measured map fit: $\vec{r}_1$=(52.3,-1.6,30.9), $\vec{r}_2$=(-46.9,1.9,32.9), $\vec{p}_1$=(10.1,-7,-17.5), $\vec{p}_2$=(-13.1,-7.3,-18.3),
d) estimated map fit: $\vec{r}_1$=(50,-1.7,28), $\vec{r}_2$=(-43.5,2.9,31.1), $\vec{p}_1$=(11,-8,-20.2), $\vec{p}_2$=(-15.5,-9.8,-20.8),
reconstructed source errors: Δr_1=3.7, Δr_2=3.9, Δr_c=5.4, $\Delta\varphi_1$=1.4, $\Delta\varphi_2$=2.77,
e) selected chan. fit: $\vec{r}_1$=(49.3,-0.9,28.6), $\vec{r}_2$=(-46,2.5,34.5), $\vec{p}_1$=(11.5,-9.3,-20.1), $\vec{p}_2$=(-14,-9.5,-18),
reconstructed source errors: Δr_1=3.9, Δr_2=1.9, Δr_c=4.3, $\Delta\varphi_1$=2.86, $\Delta\varphi_2$=5.02.

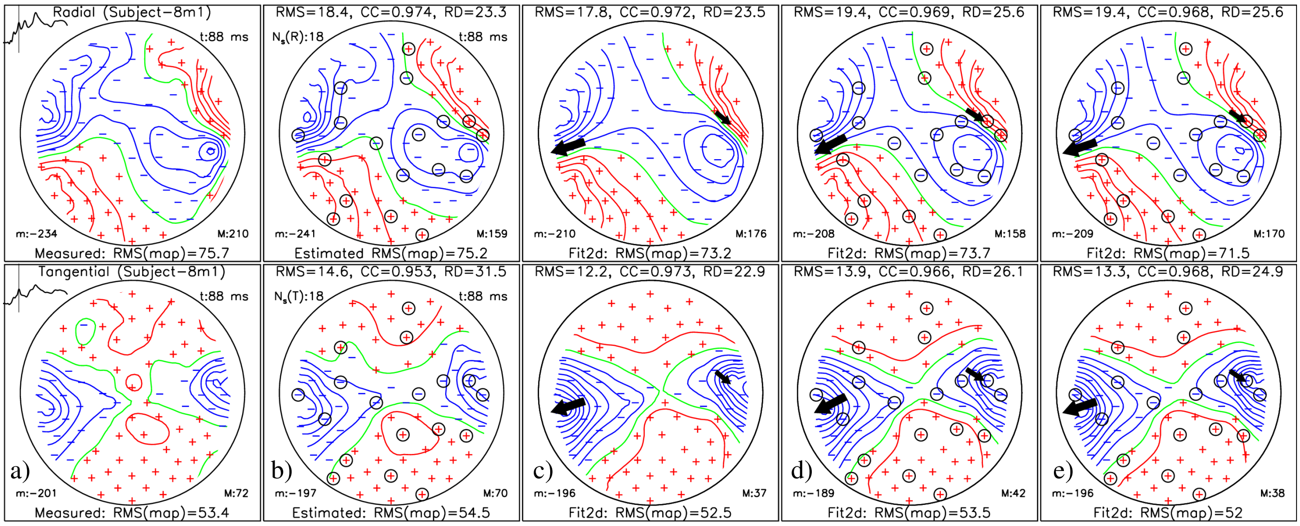

Figure S.23: Subject-8m1: 18 selected sites using **all-bases-sph**, fitting M100 with 2 dipoles:

a) Measured data, b) Estimated data map,

c) measured map fit: $\vec{r}_1$=(47.6,10.7,19.3), $\vec{r}_2$=(-38.1,-5.7,10.7), $\vec{p}_1$=(-4.8,6.2,8.4), $\vec{p}_2$=(5.5,2.5,21),

d) estimated map fit: $\vec{r}_1$=(41,11.9,22.4), $\vec{r}_2$=(-34.8,-4.5,12.2), $\vec{p}_1$=(-8.1,6.7,11.4), $\vec{p}_2$=(8,5.4,24.8),
reconstructed source errors: Δr_1=7.3, Δr_2=3.8, Δr_c=8.3, $\Delta\varphi_1$=8.73, $\Delta\varphi_2$=6.01,

e) selected chan. fit: $\vec{r}_1$=(44.9,11.6,21.3), $\vec{r}_2$=(-37.3,-5.4,5.7), $\vec{p}_1$=(-6.1,5.7,9.7), $\vec{p}_2$=(3.3,2.4,23.7),
reconstructed source errors: Δr_1=3.5, Δr_2=5, Δr_c=6.1, $\Delta\varphi_1$=6.69, $\Delta\varphi_2$=6.81.

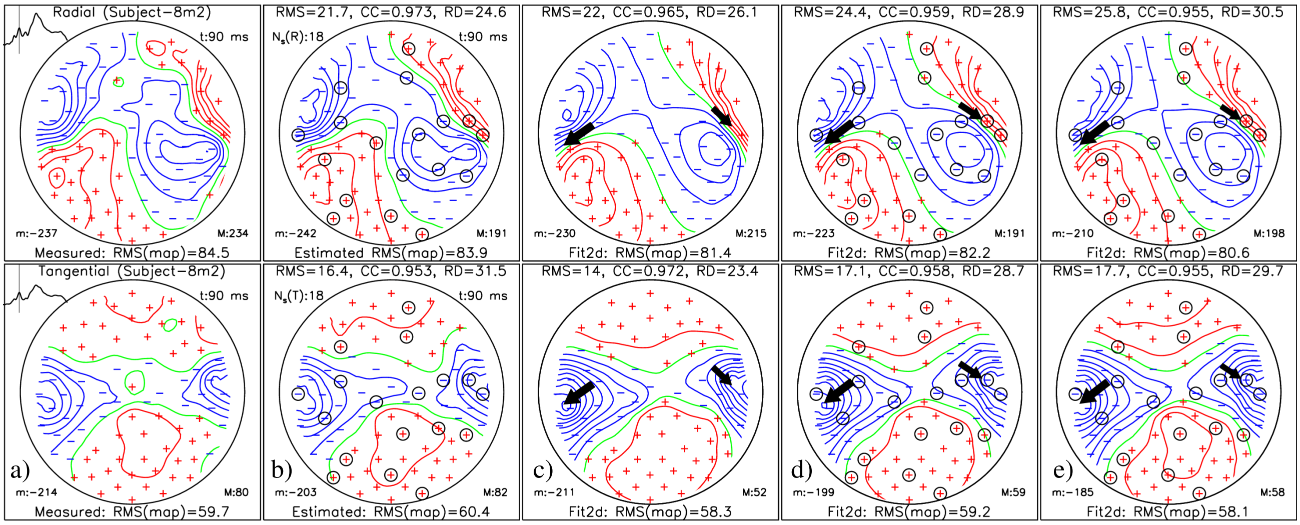

Figure S.24: Subject-8m2: 18 selected sites using **all-bases-sph**, fitting M100 with 2 dipoles:

a) Measured data, b) Estimated data map,

c) measured map fit: $\vec{r}_1$=(45.1,10.9,19), $\vec{r}_2$=(-38.5,-0.2,19.8), $\vec{p}_1$=(-6.7,9.8,10.2), $\vec{p}_2$=(9.3,6.7,18.2),

d) estimated map fit: $\vec{r}_1$=(37.1,13.6,23.6), $\vec{r}_2$=(-34.5,0.8,18.5), $\vec{p}_1$=(-12.6,10.7,13.6), $\vec{p}_2$=(12.7,10.4,23.3),
reconstructed source errors: Δr_1=9.6, Δr_2=4.3, Δr_c=10.5, $\Delta\varphi_1$=11.7, $\Delta\varphi_2$=3.54,

e) selected chan. fit: $\vec{r}_1$=(36.7,12.6,22.5), $\vec{r}_2$=(-31,0.4,14.2), $\vec{p}_1$=(-13.6,11.7,15.7), $\vec{p}_2$=(14.2,13.3,30.4),
reconstructed source errors: Δr_1=9.2, Δr_2=9.3, Δr_c=13.1, $\Delta\varphi_1$=11.3, $\Delta\varphi_2$=4.06.

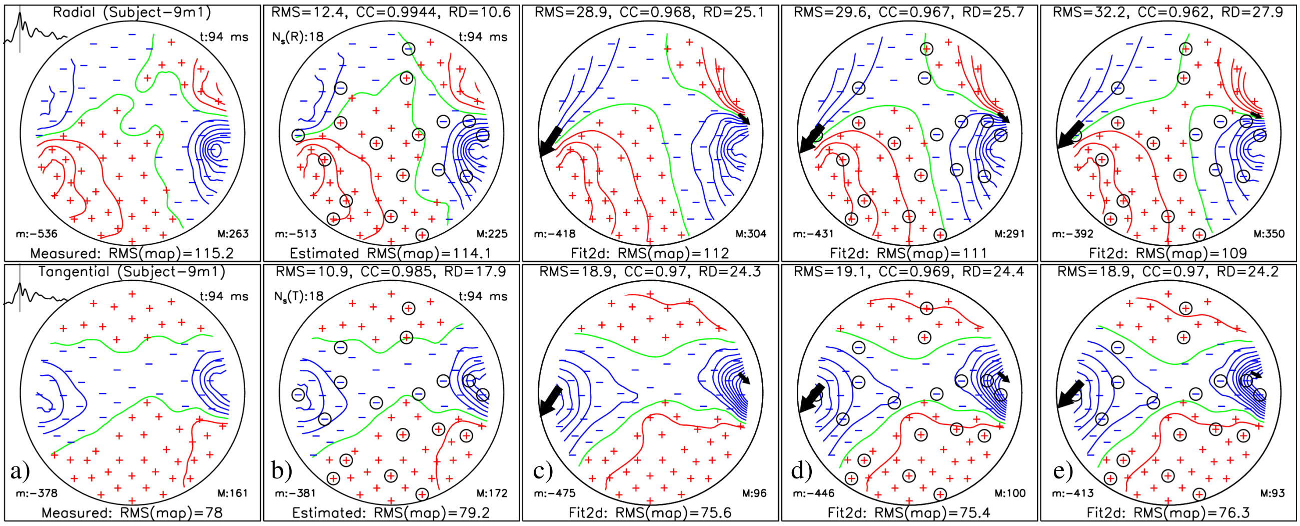

Figure S.25: Subject-9m1: 18 selected sites using **all-bases-sph**, fitting M100 with 2 dipoles:
a) Measured data, b) Estimated data map,
c) measured map fit: $\vec{r}_1$=(58,9.6,-1.2), $\vec{r}_2$=(-35,-2.6,-2.7), $\vec{p}_1$=(-1.1,8.6,13.6), $\vec{p}_2$=(-4,18.6,33.6),
d) estimated map fit: $\vec{r}_1$=(55.8,9.9,-2), $\vec{r}_2$=(-31.7,-1.4,-0.8), $\vec{p}_1$=(-1,8.9,15.9), $\vec{p}_2$=(-2,20.3,40),
reconstructed source errors: Δr_1=2.3, Δr_2=4, Δr_c=4.6, $\Delta\varphi_1$=3.13, $\Delta\varphi_2$=4,
e) selected chan. fit: $\vec{r}_1$=(57,12.5,5.3), $\vec{r}_2$=(-30.5,-0.1,-0.9), $\vec{p}_1$=(-2.3,4.8,14), $\vec{p}_2$=(-1.3,20.1,42.1),
reconstructed source errors: Δr_1=7.2, Δr_2=5.5, Δr_c=9, $\Delta\varphi_1$=14.1, $\Delta\varphi_2$=5.53.

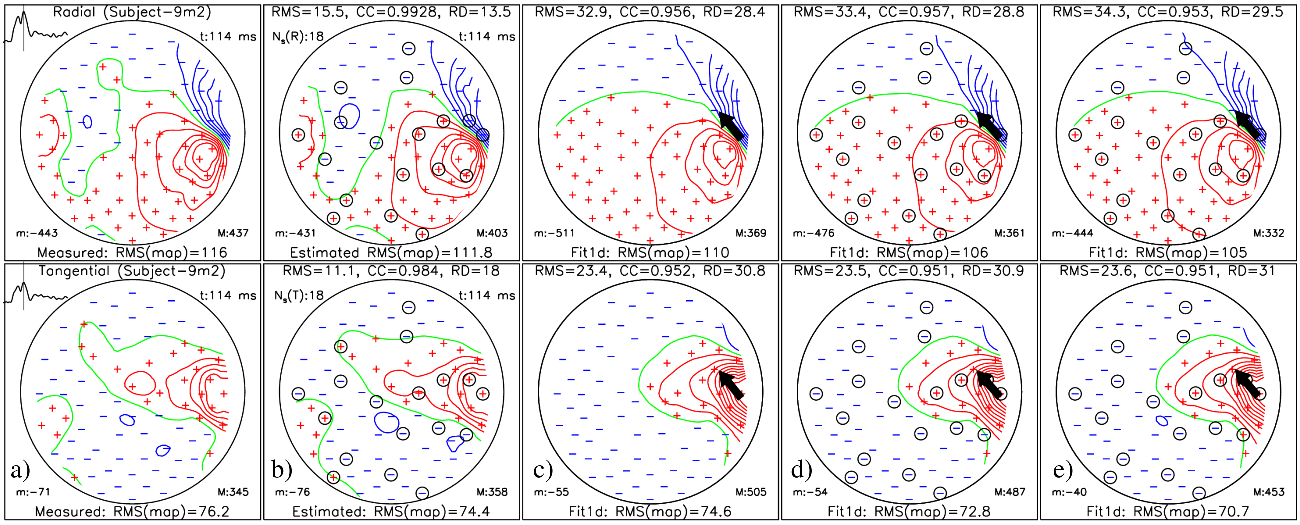

Figure S.26: Subject-9m2: 18 selected sites using **all-bases-sph**, fitting M100 with 1 dipole:
a) Measured data, b) Estimated data map,
c) measured map fit: $\vec{r}$=(55.4,6.2,15.7), $\vec{p}$=(5.2,-10.4,-14.1),
d) estimated map fit – source parameters: $\vec{r}$=(55.9,6.8,16.5), $\vec{p}$=(5,-9.2,-13.3),
reconstructed source errors: $\Delta\vec{r}$=(0.5,0.6,0.8), Δr=1.1, $\Delta\vec{p}$=(-0.1,1.2,0.9), Δp=1.5, $\Delta\varphi$=0.031,
e) selected chan. fit: $\vec{r}$=(53.6,7.1,16.5), $\vec{p}$=(5.8,-10.8,-14.4),
reconstructed source errors: $\Delta\vec{r}$=(-1.9,0.9,0.8), Δr=2.2, $\Delta\vec{p}$=(0.7,-0.4,-0.2), Δp=0.8, $\Delta\varphi$=0.03.

S.5 Localizations of M100 for all measurements using data from the right hemisphere only

Comparisons of M100 source localization for all cases using data from the right hemisphere only. First we applied SSA using SPH simulated data from all subjects and all simulation protocols (**all-bases-sph**) to find the first 9 optimal measuring sites out of 43 that covers the right hemisphere. Then we localized M100 with a single dipole source from the measured MFM, the estimated MFM and from the selected sites only. All results are displayed in the following figures. In figures's captions, all coordinates and localization errors are expressed in units of mm, dipole moments in μAm, and dipole orientation errors with angular degrees.

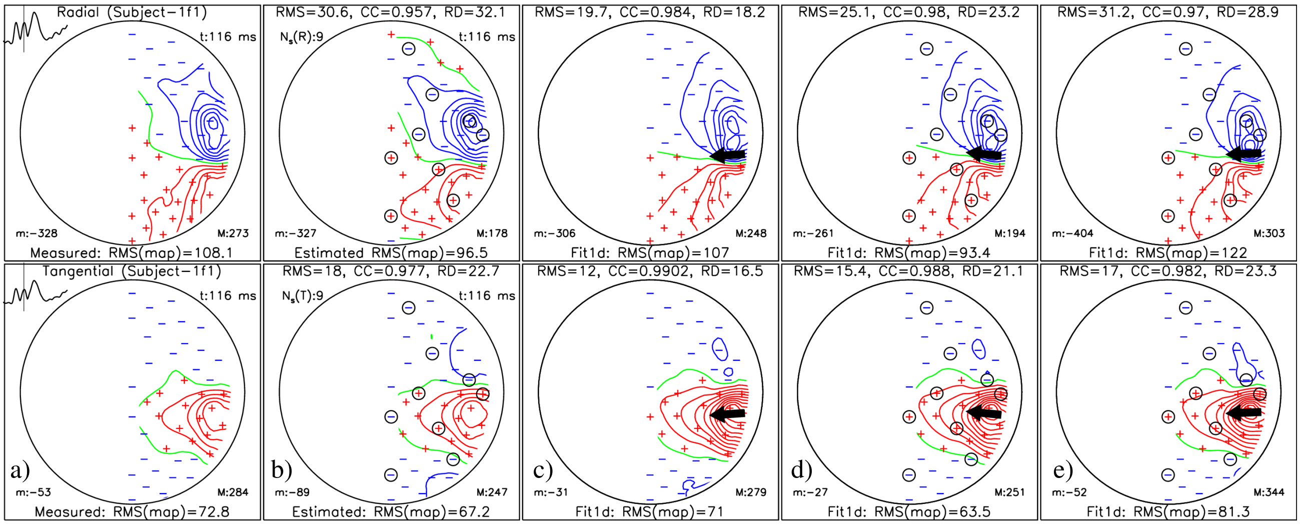

Figure S.27: Subject-1f1: 9 selected sites using **all-bases-sph**, fitting M100 with 1 dipole:
a) Measured data, b) Estimated data map,
c) measured map fit: $\vec{r}$=(59.6,-15.6,18.3), $\vec{p}$=(2.9,0.9,-8.6),
d) estimated map fit – source parameters: $\vec{r}$=(59,-15.1,21.4), $\vec{p}$=(2.7,0,-7.5),
reconstructed source errors: $\Delta\vec{r}$=(-0.6,0.5,3.1), Δr=3.2, $\Delta\vec{p}$=(-0.2,-0.9,1.1), Δp=1.4, $\Delta\varphi$=0.1,
e) selected chan. fit: $\vec{r}$=(63.5,-15.8,22.1), $\vec{p}$=(2.8,0.6,-7.6),
reconstructed source errors: $\Delta\vec{r}$=(4,-0.2,3.8), Δr=5.5, $\Delta\vec{p}$=(-0.1,-0.2,1.1), Δp=1.1, $\Delta\varphi$=0.036.

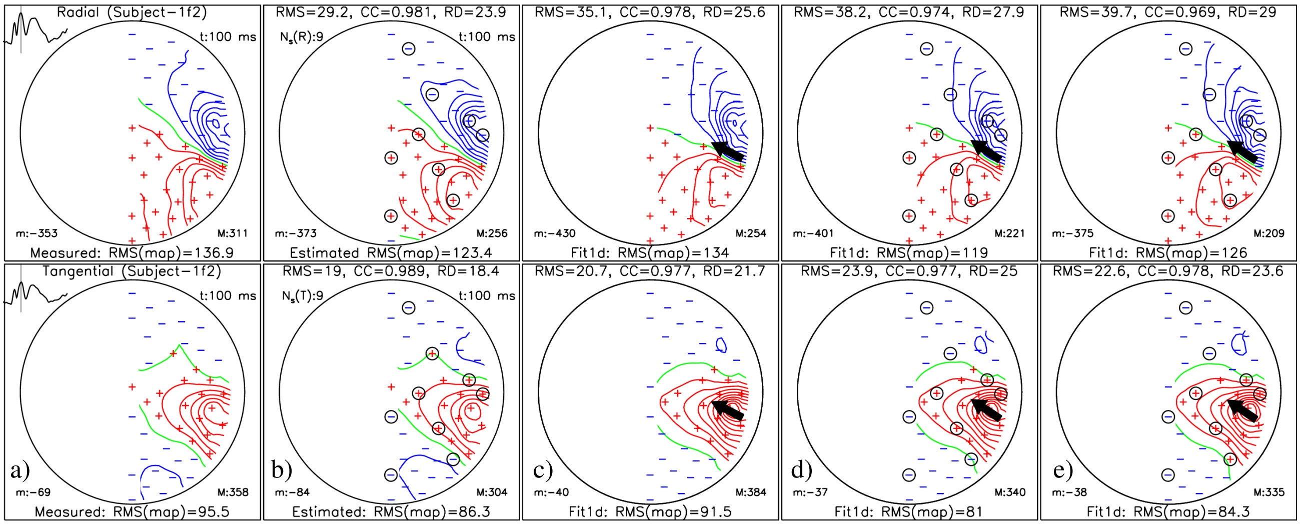

Figure S.28: Subject-1f2: 9 selected sites using **all-bases-sph**, fitting M100 with 1 dipole:
a) Measured data, b) Estimated data map,
c) measured map fit: $\vec{r}$=(57.3,-11.7,18.9), $\vec{p}$=(3.5,-2.7,-12.2),
d) estimated map fit – source parameters: $\vec{r}$=(57.2,-11.4,19.5), $\vec{p}$=(3,-3.2,-10.8),
reconstructed source errors: $\Delta\vec{r}$=(-0.1,0.3,0.6), Δr=0.7, $\Delta\vec{p}$=(-0.4,-0.5,1.4), Δp=1.6, $\Delta\varphi$=0.07,
e) selected chan. fit: $\vec{r}$=(55.5,-11.9,22.1), $\vec{p}$=(3.8,-3.8,-11.6),
reconstructed source errors: $\Delta\vec{r}$=(-1.9,-0.2,3.2), Δr=3.7, $\Delta\vec{p}$=(0.3,-1.1,0.6), Δp=1.3, $\Delta\varphi$=0.097.

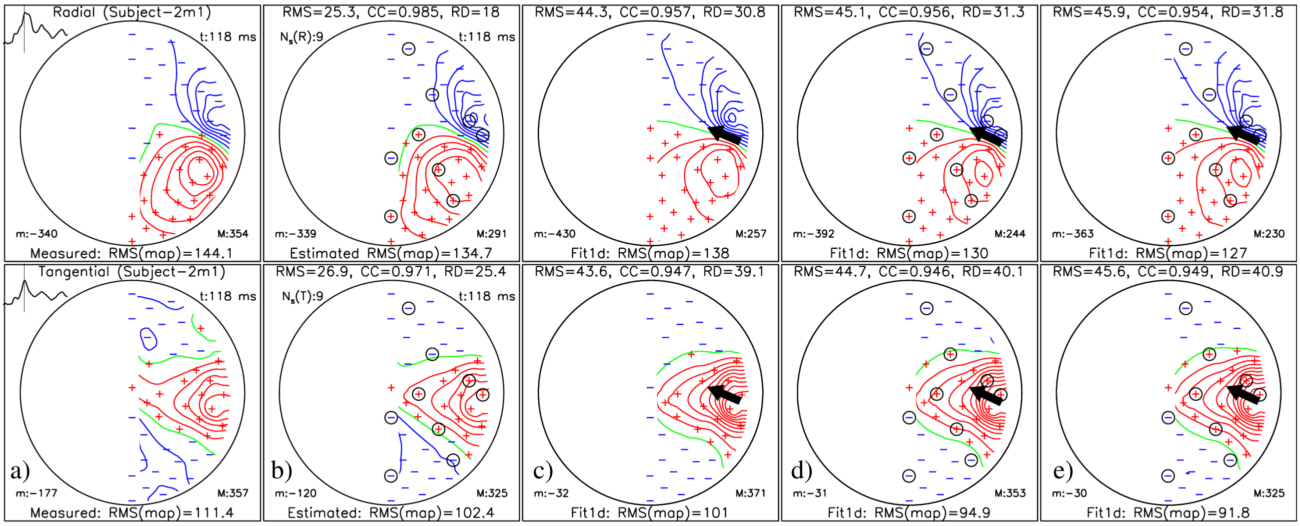

Figure S.29: Subject-2m1: 9 selected sites using **all-bases-sph**, fitting M100 with 1 dipole:
a) Measured data, b) Estimated data map,
c) measured map fit: $\vec{r}$=(53.3,0.3,22.3), $\vec{p}$=(6.2,-4,-14.8),
d) estimated map fit – source parameters: $\vec{r}$=(53,-0.4,19.5), $\vec{p}$=(5.4,-4.1,-14.9),
reconstructed source errors: $\Delta\vec{r}$=(-0.3,-0.6,-2.9), Δr=3, $\Delta\vec{p}$=(-0.8,-0.1,-0.1), Δp=0.8, $\Delta\varphi$=0.048,
e) selected chan. fit: $\vec{r}$=(50.9,0.4,20.7), $\vec{p}$=(6.3,-4.4,-15.5),
reconstructed source errors: $\Delta\vec{r}$=(-2.4,0.1,-1.7), Δr=2.9, $\Delta\vec{p}$=(0.1,-0.4,-0.7), Δp=0.8, $\Delta\varphi$=0.018.

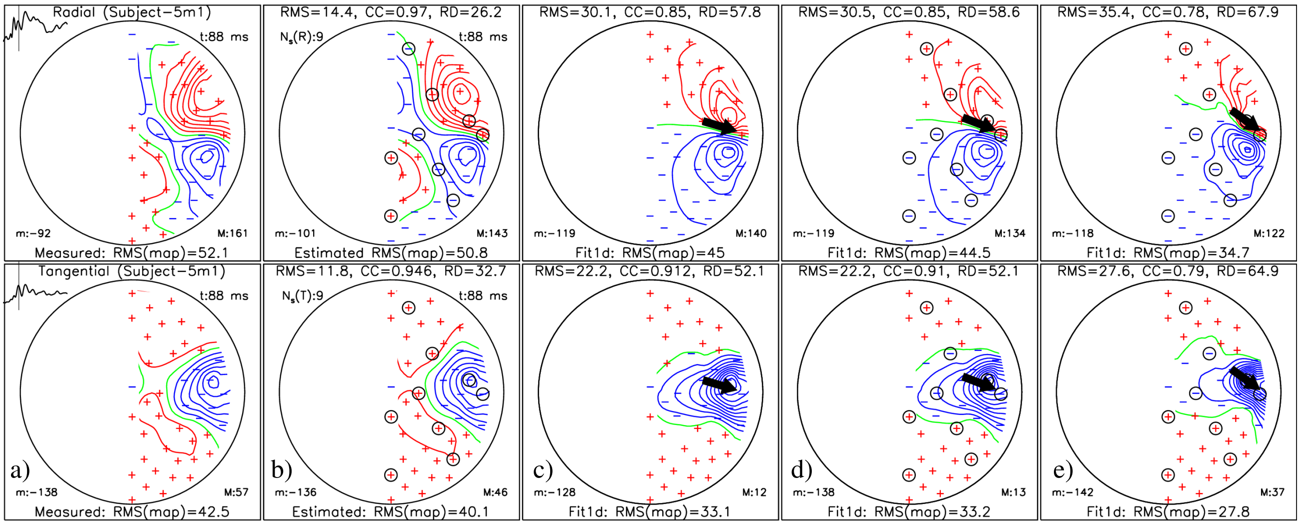

Figure S.30: Subject-5m1: 9 selected sites using **all-bases-sph**, fitting M100 with 1 dipole:
a) Measured data, b) Estimated data map,
c) measured map fit: $\vec{r}$=(59,7.2,29.2), $\vec{p}$=(-1.7,0.4,3.2),
d) estimated map fit – source parameters: $\vec{r}$=(58.6,9,28.4), $\vec{p}$=(-1.7,0.7,3.2),
reconstructed source errors: $\Delta\vec{r}$=(-0.3,1.8,-0.8), Δr=2, $\Delta\vec{p}$=(0,0.2,0), Δp=0.2, $\Delta\varphi$=0.058,
e) selected chan. fit: $\vec{r}$=(67.3,12.3,21.7), $\vec{p}$=(-0.7,0.9,1.7),
reconstructed source errors: $\Delta\vec{r}$=(8.3,5.1,-7.6), Δr=12.4, $\Delta\vec{p}$=(1,0.4,-1.6), Δp=1.9, $\Delta\varphi$=0.335.

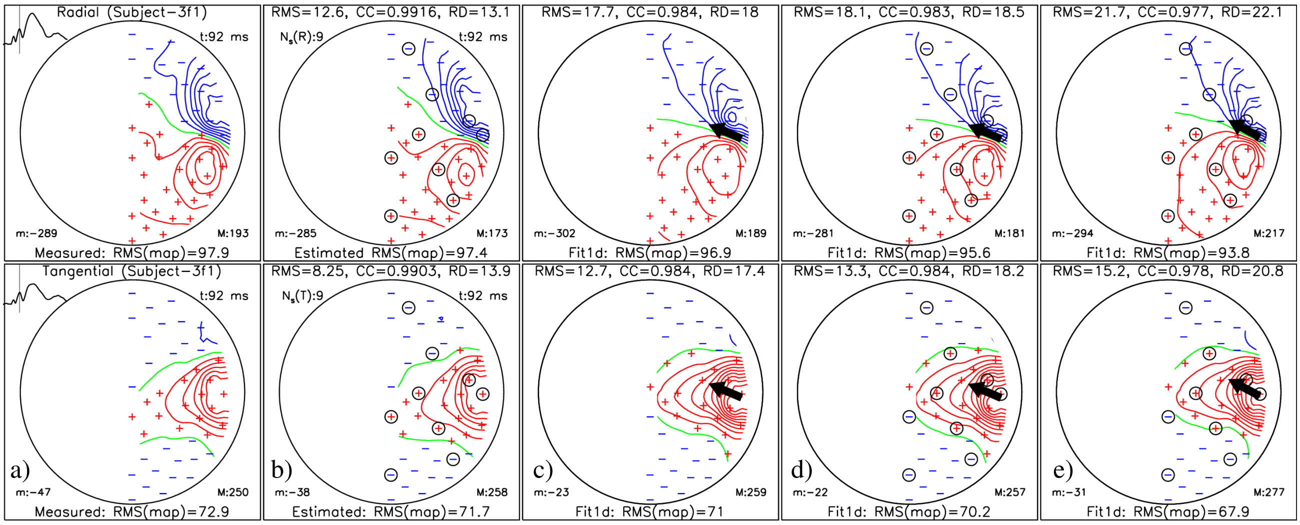

Figure S.31: Subject-3f1: 9 selected sites using **all-bases-sph**, fitting M100 with 1 dipole:
a) Measured data, b) Estimated data map,
c) measured map fit: $\vec{r}$=(55.1,2.2,21.4), $\vec{p}$=(3.9,-2.6,-9.7),
d) estimated map fit – source parameters: $\vec{r}$=(53.6,1.9,20.4), $\vec{p}$=(4.1,-2.8,-10.5),
reconstructed source errors: $\Delta\vec{r}$=(-1.6,-0.3,-1.1), Δr=1.9, $\Delta\vec{p}$=(0.2,-0.2,-0.8), Δp=0.9, $\Delta\varphi$=0.0082,
e) selected chan. fit: $\vec{r}$=(57,4.4,21), $\vec{p}$=(3.3,-2.9,-8.5),
reconstructed source errors: $\Delta\vec{r}$=(1.8,2.2,-0.4), Δr=2.9, $\Delta\vec{p}$=(-0.5,-0.3,1.2), Δp=1.4, $\Delta\varphi$=0.068.

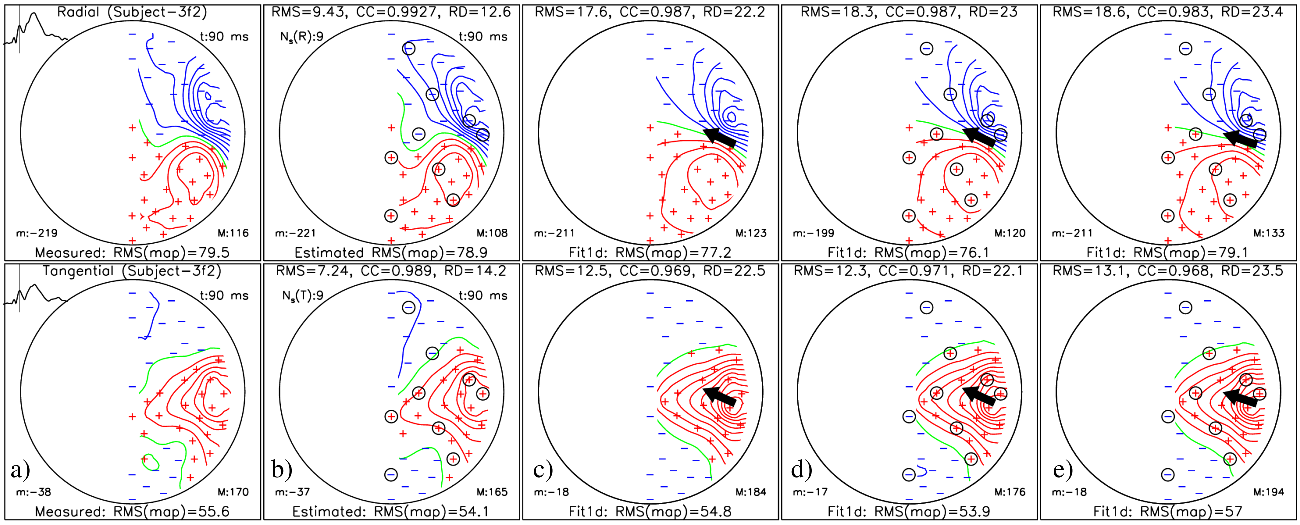

Figure S.32: Subject-3f2: 9 selected sites using **all-bases-sph**, fitting M100 with 1 dipole:
a) Measured data, b) Estimated data map,
c) measured map fit: $\vec{r}$=(46.3,-1.7,24.2), $\vec{p}$=(5.3,-3.2,-10.4),
d) estimated map fit – source parameters: $\vec{r}$=(44.9,-1.3,22.9), $\vec{p}$=(5.6,-3.5,-11.1),
reconstructed source errors: $\Delta\vec{r}$=(-1.4,0.4,-1.2), Δr=1.9, $\Delta\vec{p}$=(0.3,-0.3,-0.7), Δp=0.8, $\Delta\varphi$=0.01,
e) selected chan. fit: $\vec{r}$=(46.2,-2.9,21), $\vec{p}$=(5.2,-2.4,-11.8),
reconstructed source errors: $\Delta\vec{r}$=(-0.1,-1.2,-3.2), Δr=3.4, $\Delta\vec{p}$=(-0.1,0.8,-1.3), Δp=1.6, $\Delta\varphi$=0.098.

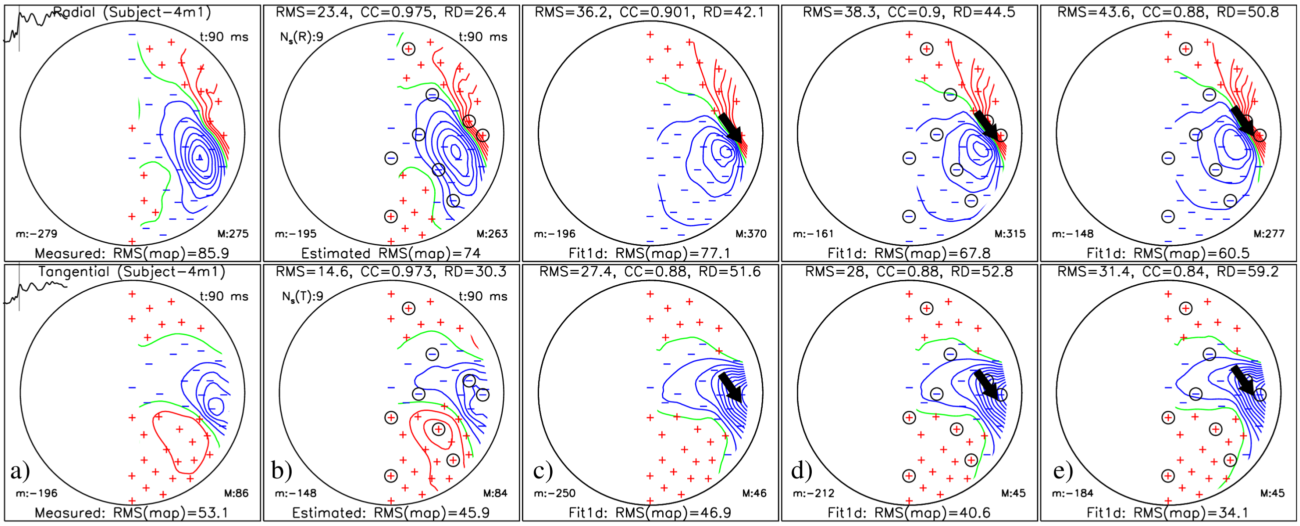

Figure S.33: Subject-4m1: 9 selected sites using **all-bases-sph**, fitting M100 with 1 dipole:
a) Measured data, b) Estimated data map,
c) measured map fit: $\vec{r}$=(58.4,4.3,15.5), $\vec{p}$=(-1.9,5.8,5.7),
d) estimated map fit – source parameters: $\vec{r}$=(58.9,6.6,19.1), $\vec{p}$=(-2,4.8,4.4),
reconstructed source errors: $\Delta\vec{r}$=(0.5,2.3,3.6), Δr=4.3, $\Delta\vec{p}$=(0,-1,-1.3), Δp=1.6, $\Delta\varphi$=0.069,
e) selected chan. fit: $\vec{r}$=(59.1,10.2,21.7), $\vec{p}$=(-1.9,4.6,3.1),
reconstructed source errors: $\Delta\vec{r}$=(0.7,5.9,6.2), Δr=8.6, $\Delta\vec{p}$=(0,-1.2,-2.6), Δp=2.9, $\Delta\varphi$=0.199.

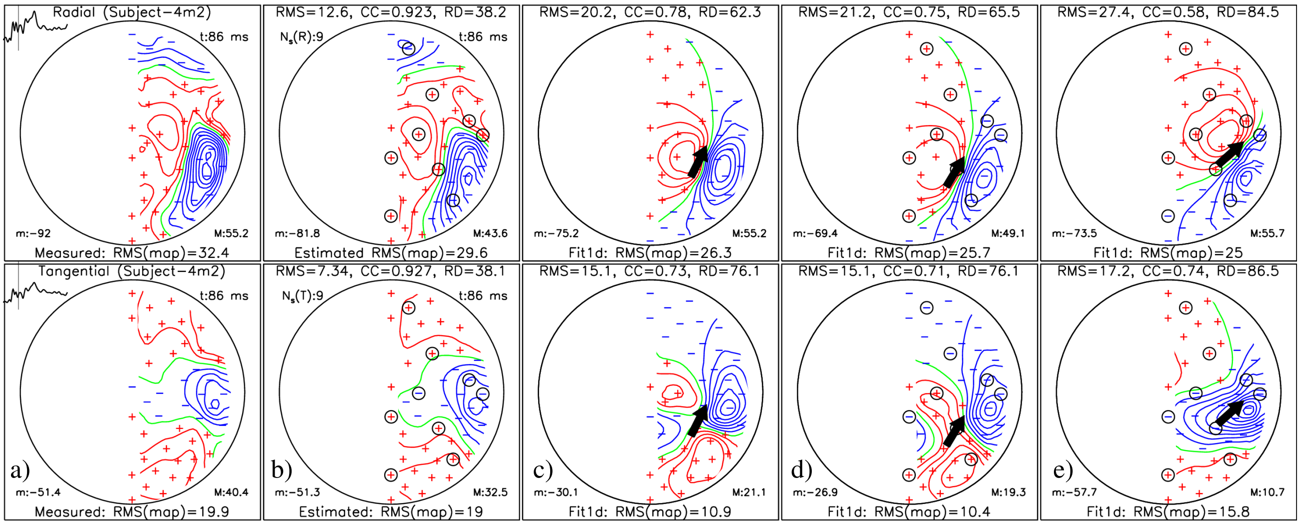

Figure S.34: Subject-4m2: 9 selected sites using **all-bases-sph**, fitting M100 with 1 dipole:
a) Measured data, b) Estimated data map,
c) measured map fit: $\vec{r}$=(49.3,-26.3,48.1), $\vec{p}$=(-0.8,-1.8,-0.1),
d) estimated map fit – source parameters: $\vec{r}$=(43,-33.6,41.7), $\vec{p}$=(-1.2,-2.2,-0.5),
reconstructed source errors: $\Delta\vec{r}$=(-6.3,-7.3,-6.4), Δr=11.6, $\Delta\vec{p}$=(-0.4,-0.3,-0.3), Δp=0.6, $\Delta\varphi$=0.146,
e) selected chan. fit: $\vec{r}$=(53.8,-15.8,34), $\vec{p}$=(-1.4,-1.6,1.4),
reconstructed source errors: $\Delta\vec{r}$=(4.5,10.5,-14.1), Δr=18.1, $\Delta\vec{p}$=(-0.5,0.3,1.6), Δp=1.7, $\Delta\varphi$=0.728.

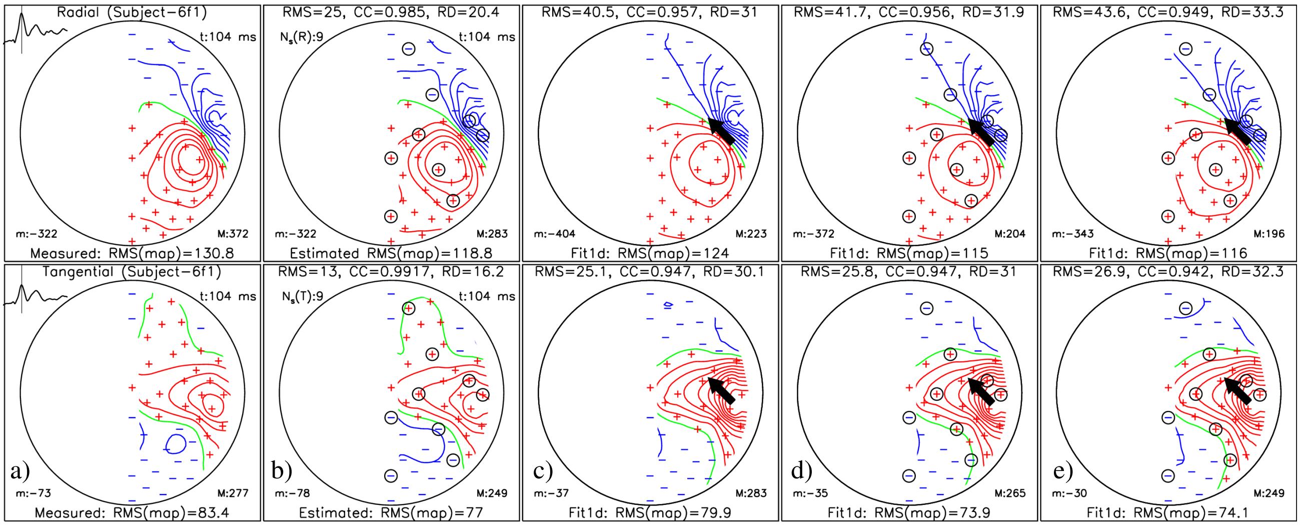

Figure S.35: Subject-6f1: 9 selected sites using **all-bases-sph**, fitting M100 with 1 dipole:
a) Measured data, b) Estimated data map,
c) measured map fit: $\vec{r}$=(51.7,3.2,25.2), $\vec{p}$=(6.1,-8.3,-11.5),
d) estimated map fit – source parameters: $\vec{r}$=(51.6,2.5,24.3), $\vec{p}$=(5.6,-7.9,-10.9),
reconstructed source errors: $\Delta\vec{r}$=(-0.1,-0.7,-0.9), Δr=1.2, $\Delta\vec{p}$=(-0.6,0.3,0.5), Δp=0.9, $\Delta\varphi$=0.019,
e) selected chan. fit: $\vec{r}$=(47.4,2.9,25), $\vec{p}$=(7.3,-9.5,-12.7),
reconstructed source errors: $\Delta\vec{r}$=(-4.3,-0.3,-0.2), Δr=4.3, $\Delta\vec{p}$=(1.2,-1.3,-1.2), Δp=2.1, $\Delta\varphi$=0.027.

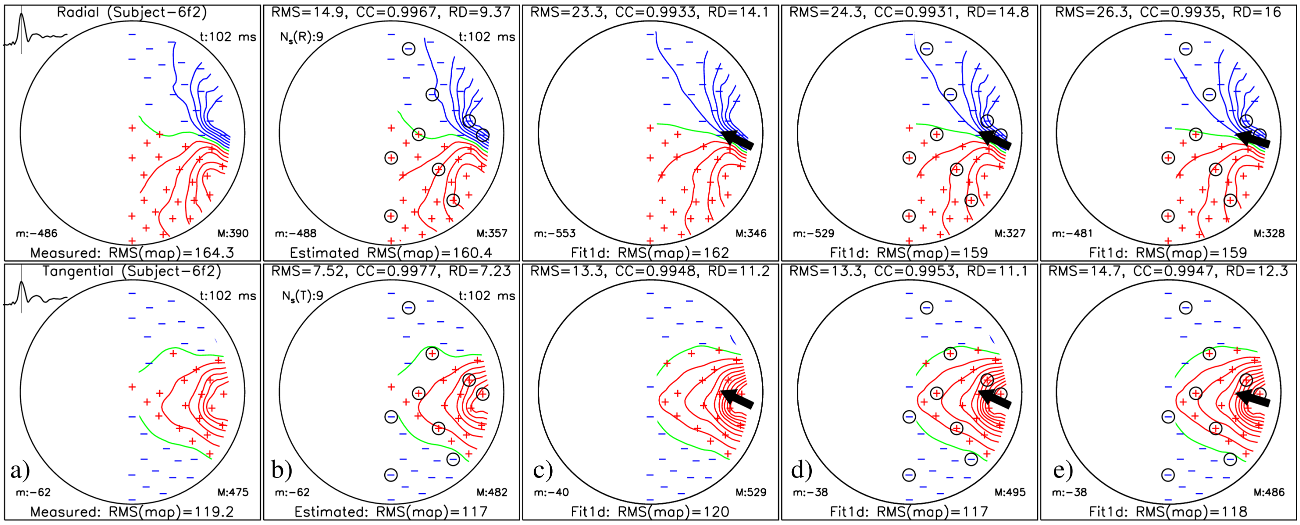

Figure S.36: Subject-6f2: 9 selected sites using **all-bases-sph**, fitting M100 with 1 dipole:
a) Measured data, b) Estimated data map,
c) measured map fit: $\vec{r}$=(53.6,-2.9,8.6), $\vec{p}$=(3.3,-4.4,-22.1),
d) estimated map fit – source parameters: $\vec{r}$=(53,-2.8,10), $\vec{p}$=(3.9,-4.4,-21.7),
reconstructed source errors: $\Delta\vec{r}$=(-0.5,0,1.4), Δr=1.5, $\Delta\vec{p}$=(0.6,0,0.4), Δp=0.7, $\Delta\varphi$=0.027,
e) selected chan. fit: $\vec{r}$=(51.8,-2.8,10.5), $\vec{p}$=(4.5,-3.1,-23),
reconstructed source errors: $\Delta\vec{r}$=(-1.8,0.1,1.9), Δr=2.6, $\Delta\vec{p}$=(1.2,1.3,-0.9), Δp=2, $\Delta\varphi$=0.076.

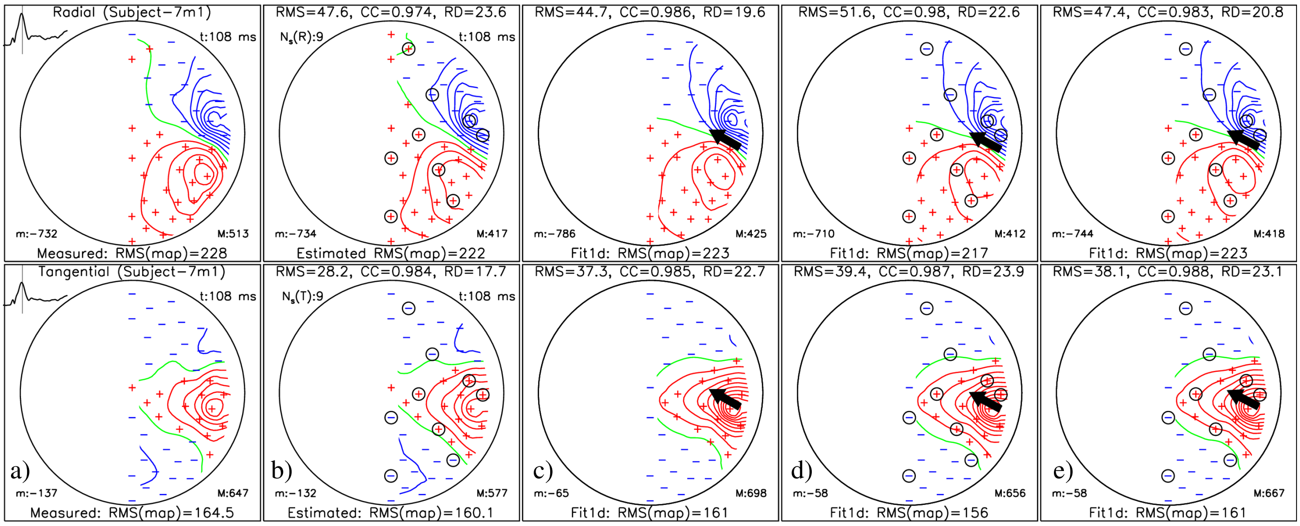

Figure S.37: Subject-7m1: 9 selected sites using **all-bases-sph**, fitting M100 with 1 dipole:
a) Measured data, b) Estimated data map,
c) measured map fit: $\vec{r}$=(57.7,-2.8,23.3), $\vec{p}$=(7.3,-6.3,-18.9),
d) estimated map fit – source parameters: $\vec{r}$=(55.5,-4.5,20.6), $\vec{p}$=(7.5,-6.3,-21.7),
reconstructed source errors: $\Delta\vec{r}$=(-2.3,-1.7,-2.7), Δr=3.9, $\Delta\vec{p}$=(0.2,0.1,-2.7), Δp=2.7, $\Delta\varphi$=0.048,
e) selected chan. fit: $\vec{r}$=(55.6,-3,22.1), $\vec{p}$=(8.2,-6.4,-21.5),
reconstructed source errors: $\Delta\vec{r}$=(-2.1,-0.1,-1.2), Δr=2.4, $\Delta\vec{p}$=(0.9,0,-2.5), Δp=2.7, $\Delta\varphi$=0.032.

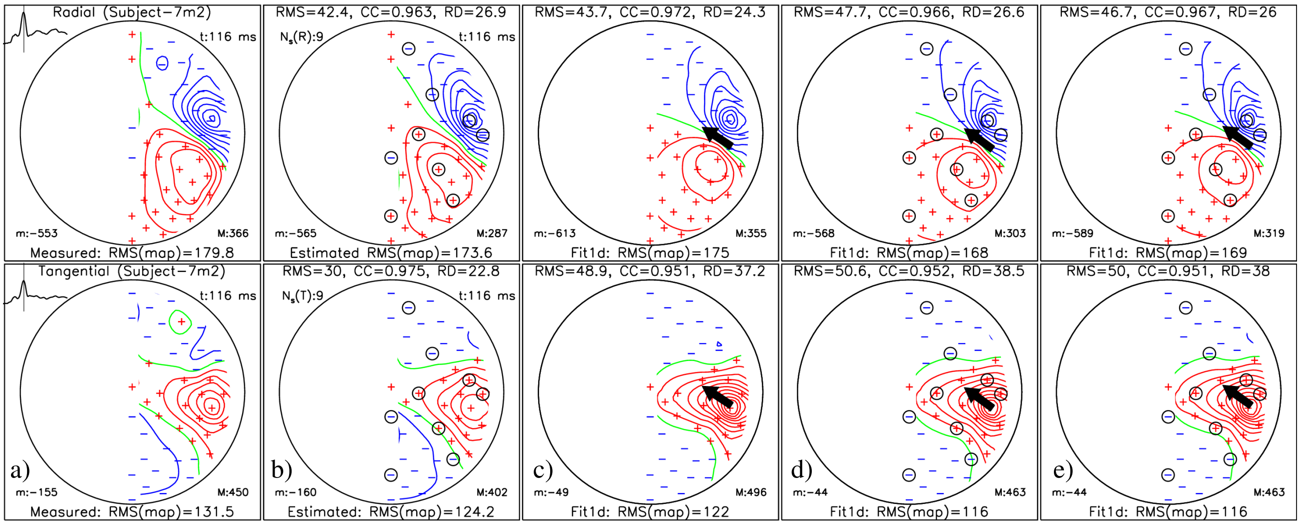

Figure S.38: Subject-7m2: 9 selected sites using **all-bases-sph**, fitting M100 with 1 dipole:
a) Measured data, b) Estimated data map,
c) measured map fit: $\vec{r}$=(56.2,-1.8,32), $\vec{p}$=(7.1,-6.1,-12.8),
d) estimated map fit – source parameters: $\vec{r}$=(53.8,-3.2,27.4), $\vec{p}$=(7.2,-7.1,-15),
reconstructed source errors: $\Delta\vec{r}$=(-2.4,-1.4,-4.5), Δr=5.3, $\Delta\vec{p}$=(0.1,-1.1,-2.2), Δp=2.4, $\Delta\varphi$=0.054,
e) selected chan. fit: $\vec{r}$=(54.6,-1.3,28.5), $\vec{p}$=(7.2,-6.9,-14.1),
reconstructed source errors: $\Delta\vec{r}$=(-1.5,0.4,-3.5), Δr=3.8, $\Delta\vec{p}$=(0.1,-0.9,-1.3), Δp=1.6, $\Delta\varphi$=0.038.

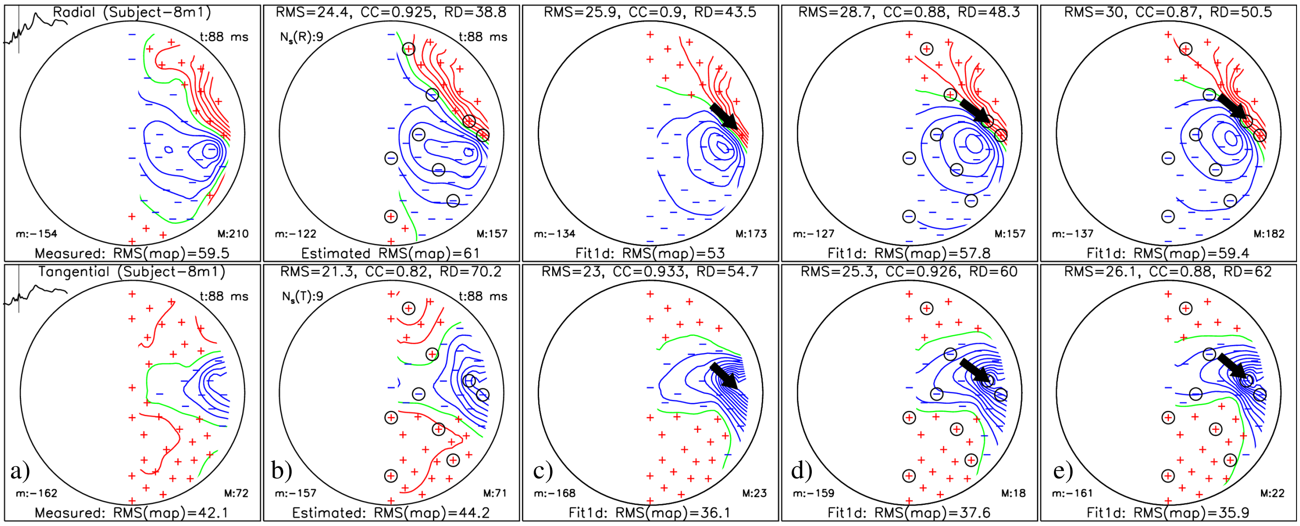

Figure S.39: Subject-8m1: 9 selected sites using **all-bases-sph**, fitting M100 with 1 dipole:
a) Measured data, b) Estimated data map,
c) measured map fit: $\vec{r}$=(57.2,13.2,21.1), $\vec{p}$=(-2.2,3.4,3.8),
d) estimated map fit – source parameters: $\vec{r}$=(49.8,16.8,25.9), $\vec{p}$=(-4,4.2,5),
reconstructed source errors: $\Delta\vec{r}$=(-7.4,3.6,4.8), Δr=9.5, $\Delta\vec{p}$=(-1.8,0.8,1.2), Δp=2.3, $\Delta\varphi$=0.148,
e) selected chan. fit: $\vec{r}$=(51.5,21.4,27.6), $\vec{p}$=(-3.9,4.5,3.7),
reconstructed source errors: $\Delta\vec{r}$=(-5.7,8.1,6.5), Δr=11.9, $\Delta\vec{p}$=(-1.7,1.1,-0.1), Δp=2, $\Delta\varphi$=0.226.

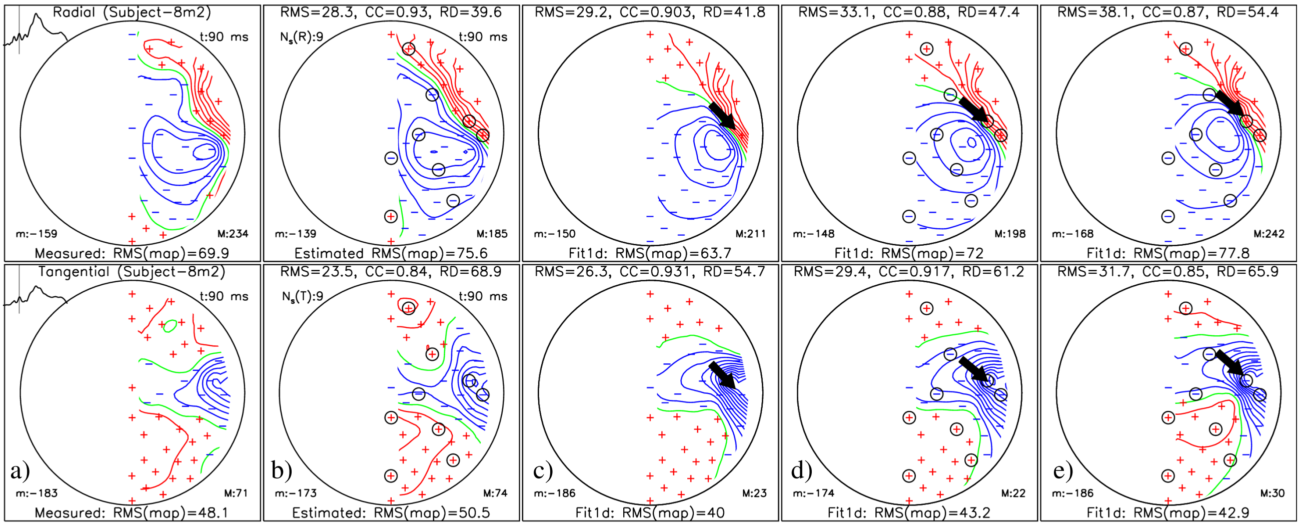

Figure S.40: Subject-8m2: 9 selected sites using **all-bases-sph**, fitting M100 with 1 dipole:
a) Measured data, b) Estimated data map,
c) measured map fit: $\vec{r}$=(54.8,13.4,21.9), $\vec{p}$=(-3.1,5,4.6),
d) estimated map fit – source parameters: $\vec{r}$=(46.8,17,25.5), $\vec{p}$=(-5.9,6.7,6.3),
reconstructed source errors: $\Delta\vec{r}$=(-8,3.6,3.5), Δr=9.4, $\Delta\vec{p}$=(-2.8,1.7,1.7), Δp=3.7, $\Delta\varphi$=0.144,
e) selected chan. fit: $\vec{r}$=(49.1,23.8,28.2), $\vec{p}$=(-5.7,6.8,4.3),
reconstructed source errors: $\Delta\vec{r}$=(-5.6,10.4,6.3), Δr=13.4, $\Delta\vec{p}$=(-2.7,1.8,-0.3), Δp=3.2, $\Delta\varphi$=0.252.

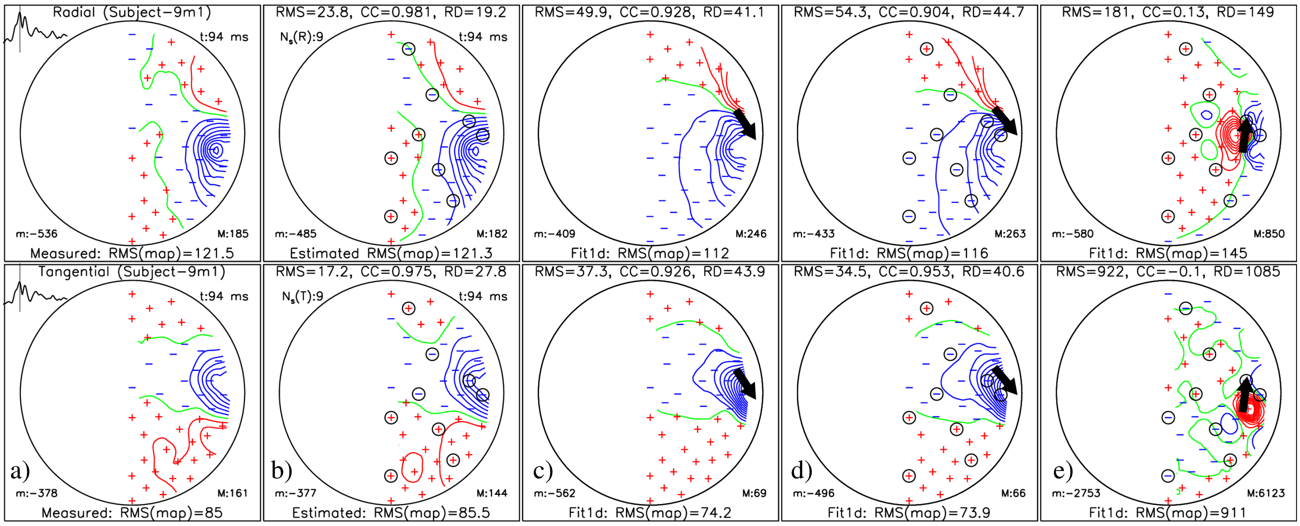

Figure S.41: Subject-9m1: 9 selected sites using **all-bases-sph**, fitting M100 with 1 dipole:
a) Measured data, b) Estimated data map,
c) measured map fit: $\vec{r}$=(64.9,6.8,-2.4), $\vec{p}$=(-0.5,7.1,7.2),
d) estimated map fit – source parameters: $\vec{r}$=(61,8.4,-3.8), $\vec{p}$=(-0.4,7.3,10),
reconstructed source errors: $\Delta\vec{r}$=(-3.9,1.6,-1.3), Δr=4.5, $\Delta\vec{p}$=(0.1,0.2,2.8), Δp=2.8, $\Delta\varphi$=0.147,
e) selected chan. fit: $\vec{r}$=(96.8,0,34.8), $\vec{p}$=(-0.1,-3.2,0.2),
reconstructed source errors: $\Delta\vec{r}$=(31.8,-6.8,37.2), Δr=49.4, $\Delta\vec{p}$=(0.4,-10.2,-7.1), Δp=12.4, $\Delta\varphi$=2.29.

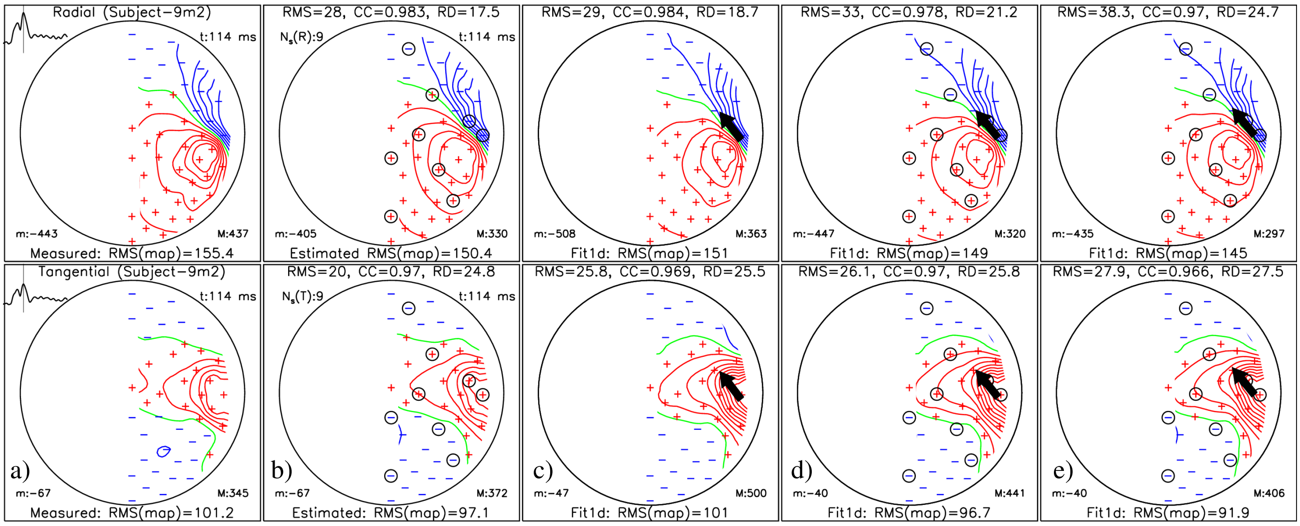

Figure S.42: Subject-9m2: 9 selected sites using **all-bases-sph**, fitting M100 with 1 dipole:
a) Measured data, b) Estimated data map,
c) measured map fit: $\vec{r}$=(54,5.9,15), $\vec{p}$=(5.6,-11.9,-15.5),
d) estimated map fit – source parameters: $\vec{r}$=(50.9,6.9,16.5), $\vec{p}$=(7.2,-13.3,-16.8),
reconstructed source errors: $\Delta\vec{r}$=(-3.1,1,1.5), Δr=3.6, $\Delta\vec{p}$=(1.6,-1.4,-1.3), Δp=2.5, $\Delta\varphi$=0.049,
e) selected chan. fit: $\vec{r}$=(50.4,8.7,18.6), $\vec{p}$=(8,-13.5,-15.3),
reconstructed source errors: $\Delta\vec{r}$=(-3.7,2.8,3.6), Δr=5.8, $\Delta\vec{p}$=(2.4,-1.6,0.2), Δp=2.9, $\Delta\varphi$=0.113.
